# Recovering Small Molecule Ligand—RNA Binding Pocket Binding Modes and Exploring Potential Binding Pathways Using Metadynamics

**DOI:** 10.3390/molecules31193428

**Published:** 2026-09-26

**Authors:** Zhixue Bai, Alan Chen

**Affiliations:** Department of Chemistry and the RNA Institute, University at Albany, State University of New York, Albany, NY 12222, USA; zhixuebai@fas.harvard.edu

**Keywords:** RNA binding pocket, small molecule ligand, metadynamics, free energy landscape, binding mode recovering, binding pathway, RNA aptamer, riboswitch aptamer domain

## Abstract

Understanding how an RNA recognizes small molecule ligands is crucial for pharmaceutical research. Molecular dynamics (MD) simulations can potentially reveal this process in atomistic detail. However, simulations frequently fail should energy barriers along the pathway prove to be insurmountably high on the simulation timescale (ns-µs). To address this limitation, we present a novel strategy combining well-tempered metadynamics utilizing X-ray crystallography or NMR-solved hydrogen-bonding interactions between an RNA and its ligand as collective variables (CVs). This approach can sample multiple transitions during the binding process and determine the most favorable binding modes through the reconstructed free energy landscape. Our computational recovery of binding modes for all five cases studied is in good agreement (RMSD below 2.0 Å) with the experimentally determined structures of the bound complexes. Our study demonstrates that metadynamics coupled with strategically chosen collective variables can effectively sample binding pathways and recover binding modes for RNA-small molecule complexes. This method therefore could be valuable for crystal structure interpretation, NMR solution structure refinement, and computer-aided design of RNA-targeted small molecule drugs.

## 1. Introduction

Understanding the mechanism of the molecular recognition process between small molecule ligands and their RNA-binding pocket is of great significance in drug discovery. There are three types of well-known small molecule ligand-RNA binding pocket recognition cases. The first type is high selectivity and high affinity (μM to nM) binding between RNA sequences identified by the techniques of SELEX (Systematic Evolution of Ligands by Exponential Enrichment) and their small-molecule target, including cofactors, antibiotics, and therapeutic targets [1,2,3]. The second type involves specific recognition between the high-affinity aptamer domain of a riboswitch located in the 5′ untranslated region of bacterial mRNAs and its small-molecule ligand of metabolic intermediate or antimicrobial agent, which can lead to active or attenuated gene expression by altering accessibility to mRNA signals necessary for transcription or translation [4,5,6,7,8]. The third type involves binding between a small-molecule splicing modulator, discovered as a treatment for human genetic disease, and single-stranded purine-rich RNA of a pre-mRNA exon at the 5′ splicing site, which can fix the weak base-complementary pairing between exon and sequence of spliceosome thereby increasing the production of mature mRNA for relevant protein expression [9,10,11,12,13,14]. However, due to inherent flexibility and complex dynamics of structured RNAs, crystal structures of small-molecule ligand-RNA complexes are extremely difficult to solve through X-ray crystallography. While NMR is able to probe the structure of RNAs in solution, structures with long RNA sequences are often difficult to obtain due to the inherent technical problem of spectral overlap [15,16]. In addition, neither X-ray crystallography nor NMR provides dynamic information related to RNA folding and molecular recognition.

The longstanding need to better understand RNA-ligand interactions has resulted in the development of many computational “docking” tools to recover ligand-RNA binding interactions [17,18,19]. However, these existing models focus on recovering the static conformation of the bound ligand-RNA complex by assuming a rigid RNA and implicit-solvent, coarse-grained energy functions to facilitate exhaustive exploration of potential interaction sites. Explicit solvent molecular dynamics simulations are a powerful method for elucidating not only the end-state tertiary structure of nucleic acid–small molecule ligand recognition at atomic resolution but also the path that molecules take to achieve the end-state [10,20,21,22,23], which thermodynamically simulates the interactions and determines the properties of the system by integrating Newton’s equations of motion for every heavy atom of your system under a specific force field for mimicking both van der Waals forces and Coulombic forces between any two heavy atoms [24]. There have been many new developments in force fields of nucleic acid systems in recent years, including the correction of erroneously strong base stacking, base pairing, ionic interactions, and parametrizations against experimental data [7,25,26,27,28,29,30], all of which entail explicit representations of the aqueous solvent. However, the associated long time scale for simulating the real binding process is difficult to access via conventional unbiased MD simulations due to the high energy barriers needed to be overcome in the binding pathway [31,32]. In response to this challenge, MD was used in conjunction with various biasing algorithms to flatten the free energy surface and accelerate sampling [33], including umbrella sampling [34,35,36,37], steered molecular dynamics [20,37,38], accelerated molecular dynamics [10,32,39], metadynamics [22,40,41,42], and more. Compared to other enhanced sampling methods, the metadynamics algorithm is more powerful, as it is able to probe free energy landscapes by directly filling free-energy basins with a Gaussian potential and accelerating conformational transitions along multiple reaction coordinates in three-dimensional space with selectable collective variables, unlike umbrella sampling, which requires running multiple thermodynamic windows and laborious post-processing to add a bias potential in just one dimension, which causes exponential growth in the number of windows for adding potential in three-dimensional coordinate space of XYZ. Metadynamics is biased from the history of experienced configurations by a sum of Gaussian contributions centered along the trajectory of selected CVs. This process of filling local minima with Gaussian potential discourages revisiting experienced states and encourages exploring new conformational space, which brings the system across the free energy barriers and realizes conformational transitions. Moreover, the well-tempered metadynamics limits the extent of exploration of the free energy landscape via a bias factor, which effectively prevents distorted sampling caused by depositing a constant Gaussian height over time in standard metadynamics [43]. These features of metadynamics are especially useful for determining the binding pathway of a small-molecule ligand to flexible receptors [7,42].

Here we provide a new sampling strategy for exploring possible ligand-binding pathways, while recovering the binding modes of a small-molecule ligand-RNA binding pocket system using well-tempered metadynamics coupled with upper_walls restraints. This method can incorporate hydrogen-bonding information between the small-molecule ligand and its RNA binding pocket from experimentally solved crystal structures or NMR solution structures to assess the free energy landscape of the whole binding process of the recognition between the small-molecule ligand and its RNA binding pocket, including barriers and intermediate minima, and accurately probe the most energy-favorable binding mode. To demonstrate the validity of the methodology, we studied five well structural-solved cases of: (i) flavin mononucleotide (FMN) bound with 35-nucleotide RNA aptamer identified through in vitro selection [3], (ii) preQ1 riboswitch aptamer in the metabolite-bound state [5], (iii) ligand-bound adenine riboswitch aptamer domain [6], (iv) RNA aptamer complexed with citrulline and (v) RNA aptamer complexed with arginine [44] (Figure 1) (Figure 2). In each case, our simulation-recovered binding mode had good agreement (RMSD below 2.0 Å) with experimental geometry solved by X-ray crystallography or NMR. Moreover, we presented the molecular dynamics binding pathway and binding mechanism for the first three cases, FMN/RNA aptamer, PreQ1/riboswitch aptamer, and adenine/riboswitch aptamer domain. The good agreement of our computational recovery with the experimentally solved structure and the molecular dynamics insights provided in our study announced an important advance in RNA-ligand binding investigations and demonstrated that metadynamics can effectively sample state transitions in the ligand-binding process. It should be noted that this is substantially more complex than a simple “redocking” calculation where the ligand-sized cavity persists in the structure after deletion of the bound ligand, as the inclusion of explicit solvent results in instantaneous collapse of the ligand-binding pocket to replace lost ligand-RNA interactions with non-native RNA-RNA interactions. This method could therefore be potentially valuable for exploring potential binding pathways that explain how RNA can selectively recognize small molecules despite being very dynamic and flexible.

## 2. Materials and Methods

### 2.1. System Setup

The starting coordinates of five small-molecule ligand-RNA aptamer complexes were derived from the Protein Data Bank (PDB IDs 1FMN, 3Q50, 5SWE, 1KOD, and 1KOC). The model 1 NMR solution structure of five conformers in the NMR ensemble was used for the first case of 1FMN. Each of the other four cases has only one structure in its PDB entry. Five small-molecule ligands, including FMN, preQ1, adenine, citrulline, and arginine, were parameterized by Q-Chem and ACPYPE. Q-Chem 5.2 [45] was used to calculate optimized molecular geometry and RESP charges with the Hartree–Fock method and the basis sets 6-31G(d) and cc-pVDZ (File S1). Topology files of small molecule ligands were produced by ACPYPE [46]. The GROMACS 2019.6 software package [31,47,48,49,50], compiled with PLUMED 2.7.0 [43,51,52,53,54], was used for metadynamics simulations of five systems of small molecule ligand-RNA aptamer complexes with water and ions in a box model. We chose the TIP4P explicit water model and a revised force field for RNA by Chen and Garcia et al. [55], which corrected the issue of hyperstacked structures of the Amber-99 force field caused by the erroneously strong base-stacking propensity; the Steinbrecher and Case bioorganic phosphate parameters were used to prevent formation of spurious phosphate-base hydrogen bonds [26]. For all the metadynamics simulations in this study, 150 mM excess KCl using the Cheatham-Young TIP4P-adapted ion parameters [56] was used for neutralizing negatively charged RNAs, and 300 K was chosen as the simulation temperature to optimize the chances of observing reversible RNA-ligand binding events [35]. All of the simulations were performed in a cubic box with periodic boundary conditions applied to the x, y, and z directions. The distance between the box edge and the complex of small-molecule ligand-RNA aptamer was set to 1.0 nm.

All systems were minimized for a maximum of 50,000 steps to remove unnatural contacts [57]. Subsequently, 1 ns of MD equilibration under NVT and NPT conditions at 300 K and 1 bar was performed for each system. The long-range electrostatic interactions were calculated by the Particle Mesh Ewald [58,59] method. The cutoff for nonbonded interactions was set to 1.2 nm, and the integration time step was set to 2 fs. The velocity-rescale algorithm [60] was employed for temperature coupling and the Parrinello-Rahman algorithm [61] for pressure coupling. After equilibration, well-tempered metadynamics simulations were carried out. VMD 1.9.4a43 [62] and PyMOL 2.5.2 were used for data analysis and visualization. RMSD calculations were performed by pymol align functionality with sequence alignment (BLOSUM62), followed by atom mapping and Kabsch rigid-body superposition. The number of refinement cycles was set to 0 to prevent iterative outlier rejection and ensure all-atom RMSD. RMSD statistical definition is the standard arithmetic definition of Euclidean coordinate distance.

### 2.2. Theory of Well-Tempered Metadynamics

We briefly review here the principles of the well-tempered metadynamics method. A complete theory can be found in [62,63]. Metadynamics is biased by an external history-dependent potential built as a sum of Gaussians centered along the trajectory, which is constructed in the space of the predefined collective variables [64]. The bias potential at time *t* is given by:

(1) $$V\left(\vec{S}\left(x\right),\;t\right)=W\left(k\tau \right)\underset{k\tau <t}{\sum }exp\left(-\frac{\vec{S}\left(x\right)-\vec{S}{\left(k\tau \right)}^{2}}{2\sigma^{2}}\right)$$

In Equation (1), σ is the width of the Gaussians, *W*(*k*τ) is the height of the Gaussians, and τ is the Gaussian deposition stride, which means each Gaussian is deposited every τ MD timesteps $\vec{S}$(*x*) denotes the value of CVs [65]. The bias potential of metadynamics can make the system escape local minima to accelerate conformational transitions along selected CVs. At long times, the value of the bias potential converges to the projection of the free energy onto the CVs:

(2) $$V\left(\vec{S}\left(x\right),\;t\to \infty \right)=-\Delta G\left(\vec{S}\right)+c$$

In direct metadynamics, the Gaussian height was set to a constant value. As a result, the value of the bias potential oscillates rather than converging [66]. However, in well-tempered metadynamics, convergence of the bias potential can be guaranteed by reducing the Gaussian height in areas that are already strongly biased [43]:

(3) $$W\left(k\tau \right)=W_{0}exp\left(-\frac{V\left(\vec{S}\left(k\tau \right),k\tau \right)}{k_{B}\Delta T}\right)$$

In Equation (3), *W*_0_ is the initial Gaussian height, *k_B_* is the Boltzmann constant, and Δ*T* is an additional hyperparameter with the dimension of temperature. With this rescaling of the Gaussian height, the bias potential smoothly converges to a fraction of free energy:

(4) $$V\left(\vec{S}\left(x\right),\;t\to \infty \right)=-\frac{\Delta T}{T+\Delta T}\Delta G\left(\vec{S}\right)+c$$

In Equation (4), *T* is the temperature of the system. (*T* + Δ*T*) is the effective temperature experienced along the CVs, which is higher than the system temperature *T*. Therefore, the parameter Δ*T*, or “bias factor” ($\frac{T+\Delta T}{T}$) controls the extent of free energy exploration and strength of tempering: Δ*T* = 0 corresponds to unbiased sampling. Conversely, Δ*T*→∞ corresponds to conventional metadynamics with constant Gaussian height.

In general, the bias of metadynamics constructed from the history of experienced configurations flattens the free energy landscape by filling up all the free-energy basins on it, which makes deep free-energy basin regions accessible. Meanwhile, well-tempered metadynamics can limit the extent of exploration of the free energy landscape via bias factor [67]. The bias factor decreases the Gaussian height over time based on ΔT, which makes the bias potential naturally self-limit and converge smoothly to a fraction of the actual free energy surface instead of distorting the underlying probability distribution by depositing constant Gaussian height over time as in conventional metadynamics. In this way, the deposition rate slows down automatically whenever the system meets energy-favorable states, which ensures the system explores the phase space accurately in a single, well-behaved run. In metadynamics, to map the free energy landscape of a simulation, free energy is calculated from the sum of the Gaussians. Thus, the energy of certain conformations with relevant values of CVs can be obtained, which provides a quantitative method for structural stability comparison. Moreover, since metadynamics can efficiently sample conformations of the transition state along the trajectory, it is also possible to discover the reaction pathways of metastable states via metadynamics [57].

### 2.3. Method Design and Workflow

#### 2.3.1. Choice of Collective Variables

The selection of collective variables influences the effectiveness of metadynamics significantly. The ideal collective variable should be able to push the system to explore the high free energy states of interest by taking different values in all relevant metastable states and the transition states between them, which are not accessible when the system is trapped in a local minimum [63,66].

In this study, we aim to simulate the whole induced-fit binding pathway of the recognition between a small-molecule ligand and its RNA aptamer binding pocket and expect that the binding mode of the small-molecule ligand in the free-energy basin of the free energy surface (FES) recovered by metadynamics should be in good agreement with the complexed structure solved by X-ray crystallography or NMR. In unbiased MD simulations, the binding/unbinding events of a small-molecule ligand-RNA system are not accessible on the timescale of hundreds of nanoseconds due to the high energy barriers in the binding/unbinding pathway. Therefore, to fill all of the free-energy basins in the binding/unbinding pathway with an extra Gaussian potential and overcome the energy barriers of ligand binding events, the center-of-mass (COM) distance between the small-molecule ligand and its RNA binding pocket was selected as the first CV (d of CVs), the value changing of which exactly mimics the binding/unbinding events of the ligand. To enhance the motion and rotation of the groups of the small-molecule ligand within the RNA binding pocket and test the importance of hydrogen bonds captured by X-ray crystallography or NMR, two of the hydrogen bond donor-to-acceptor distances were selected as the second and third CVs (d1, d2). Different value combinations of (d, d1, d2) correspond to different states in the binding/unbinding pathway or different binding modes of a small-molecule ligand within the RNA binding pocket. The two-dimensional FES on (d1, d2) indicates whether the two hydrogen bonds represented by d1 and d2 are necessary for the free-energy basin binding modes. For the definition of the binding pocket, we chose all the nucleotides that interacted with the small-molecule ligand via hydrogen binding or base stacking captured by X-ray crystallography or NMR.

#### 2.3.2. Optimization of Metadynamics Parameters

The implementation of metadynamics simulation combined with restraints is widely applied to improve the efficiency of sampling [68]. To prevent sampling from the uninformative regions outside of the RNA aptamer binding pocket, the harmonic wall restraint, upper_walls, was used on “d” of each CV. The harmonic potential at value *x* of d is given by:

(5) $$V\left(x\right)=\left\{\begin{array}{cc} k{\left(\left(x-r\right)/s\right)}^{2} & ,\;if\;x>r\\ 0 & ,if\;x<r \end{array}\right.$$

where *k* is the force constant, *r* is the upper wall threshold, and *s* is a rescaling factor [69]. The upper_walls restraints placed on the COM distance between the small-molecule ligand and its RNA binding pocket can limit the range of motion of the ligand to a spherical area with the COM of the RNA binding pocket as the center. To prevent the ligand from moving too far from the COM of the RNA binding pocket and ensure effective interaction between the ligand and the RNA binding pocket along the trajectory of the simulation, the radius r of the spherical region of upper_walls restraints is determined by the size of the RNA binding pocket, depending on the different ligand-RNA system. There is no bias of upper_walls restraints within the spherical area, which only acts on d when the ligand moves out of the spherical area representing the RNA binding pocket. 

Since the fluctuation of CVs was limited by upper_walls restraints, the width of Gaussians (σ) was determined by 1/3 of the radius of upper_walls restraints. The combination of initial Gaussian height (*W*_0_) and bias factor (γ) should ensure that the ligand has sufficient potential to move and rotate frequently within the RNA binding pocket and the metadynamics simulation can converge in the end with the Gaussian height dropping to near zero. Direct metadynamics simulations with different values of Gaussian height in parallel were run to optimize *W*_0_, after which well-tempered metadynamics simulations with different values of the bias factor in parallel were run to optimize γ. Gaussians were deposited every picosecond (500 steps). The optimized metadynamics parameters for each system are summarized in Table 1.

To evaluate the convergence of the metadynamics simulations and establish the unbiased reference free energy, all simulations were reweighted to remove the effect of metadynamics bias on the free energy surface. The unbiased distribution of each CV was recovered using the reweighting algorithm introduced by Branduardi et al. [70].

## 3. Results

This study simulated the molecular recognition process of five systems of small-molecule ligand-RNA binding pocket complexes via metadynamics. The binding modes at the free-energy basin minima recovered by metadynamics are in good agreement with the solved structures in the Protein Data Bank. The five systems studied are: the NMR solution structure (Model 1) of the complex of flavin mononucleotide bound to a 35-nucleotide RNA aptamer identified through in vitro selection, crystal structure of preQ1 riboswitch aptamer in the metabolite-bound state, crystal structure of ligand-bound adenine riboswitch aptamer domain, NMR solution structure of RNA aptamer complexed with citrulline, and NMR solution structure of RNA aptamer complexed with arginine.

### 3.1. Fmn/RNA Aptamer

The first small molecule ligand-RNA binding pocket system studied is the FMN/RNA aptamer complex.

In the solved NMR solution structure (Model 1) [3] in the Protein Data Bank (PDB ID: 1FMN), the uracil-like edge of the isoalloxazine ring of FMN formed an A·U Reverse Hoogsteen pairing with the Hoogsteen edge of A26 via two hydrogen bonds of A26:N6-H…FMN:O2 and FMN:N3-H…A26:N7. The isoalloxazine ring of the FMN base is stacked between the G·U·A triplet and the G·G mismatch above and below (Figure 5B). Therefore, to simulate the whole binding process of FMN using metadynamics, we chose the center-of-mass distance between FMN and the binding pocket as the first collective variable (d of CVs). The initial structure of the simulation is the NMR solution structure with FMN moved 3.3 nm away from the center-of-mass of the binding pocket. The binding pocket is defined by the G10·U12·A25 triplet and the G9·G27 mismatch above and below FMN binding nucleotide, A26. To test the importance of these two hydrogen bonds for binding, we chose the hydrogen bond donor-to-acceptor distances of these two hydrogen bonds as the second and third CVs (d1 and d2) (Figure 5A).

In the simulation, FMN was pushed towards the major groove of the binding pocket by upper_walls restraints instantly, as shown in Figure 3A and Movie S1; the center-of-mass distance between FMN and the binding pocket dropped to 0.8 nm from 3.3 nm within the first couple of frames. Afterwards, the range of motion of FMN was limited by the upper_walls restraints to a spherical area with the COM of the binding pocket as the center and a radius of 0.8 nm. There are no restraints inside this spherical area, so FMN has the freedom of trying various binding poses in this range. Under the effect of a Gaussian potential of 0.3 kJ/mol on three CVs (Figure 3B), by filling up free-energy basins in the binding pathway with Gaussians, FMN obtained adequate energy for overcoming the energy barriers of state transition so that it achieved movement and rotation as much as possible, which means the achievement of different binding modes of FMN in the RNA binding pocket as many as possible. Under an optimized value of bias factor of 4.0, which means four times hotter on three CVs in total, the height of the Gaussian decreased whenever the FMN ligand reached energy-favorable binding modes to keep FMN staying in this state. On the contrary, when FMN met an energy unfavorable pose, Gaussian height was strengthened to force FMN leaving this state so that it will continue exploring more energy favorable binding modes. After repeating this process, when the most energy-favorable binding mode was found, the Gaussian height dropped to near zero without rebounding at the end of the simulation, as shown in Figure 3B, which is a sign of good convergence of the metadynamics simulation.

Compared with unbiased molecular dynamics simulation, the movement intensity of the FMN ligand was significantly increased. The extra energy comes from the Gaussian potential being added to the three collective variables (CVs). The COM distance between FMN and the binding pocket (d of CVs), the distance between hydrogen bond donor A26:N6 and hydrogen bond acceptor FMN:O2 (d1 of CVs), and the distance between hydrogen bond donor FMN:N3 and hydrogen bond acceptor A26:N7 (d2 of CVs). Adding a Gaussian potential to these three CVs forced their value to change constantly so that the FMN ligand can realize the high-intensity motion of mimicking the FMN binding process, which is unrealizable in unbiased MD simulation. Meanwhile, each value combination of these three CVs represents a specific binding mode. As shown in the final result of the free energy surface calculation in Figure 4, the deepest free-energy basin is located at d = 0.14 nm, d1 = 0.29 nm, and d2 = 0.30 nm in both biased and unbiased (reweighted) FES that were shape-overlapped (Figure 4A–C). The shape-overlapping effect of biased and unbiased FES is another sign of good convergence of the metadynamics simulation. The value of d1 lower than or equal to 0.35 nm means the hydrogen bond formation between FMN:O2 and A26:N6, and the value of d2 lower than or equal to 0.35 nm means the hydrogen bond formation between FMN:N3 and A26:N7. Thus, this free-energy basin conformation (Figure 4D,E) refers to the binding mode of FMN at the very end of this 30 ns-long metadynamics simulation (Movie S1) when the two hydrogen bonds of d1 and d2 formed at the same time (Figure 5A). As shown in Figure 5, the binding mode of FMN at the deepest free-energy basin of the simulation (Figure 5A) is very close to the NMR solution structure (Figure 5B) (RMSD = 1.98 Å), with the same two hydrogen bonds between the uracil-like edge of the isoalloxazine ring of FMN and the Hoogsteen edge of the A26, G10·U12·A25 triplet and the G9·G27 mismatch above and below the binding site.

Moreover, the second deepest free-energy basin was located at d1 = 1.16 nm, d2 = 0.49 nm, and d = 0.44 nm, which corresponds to the binding mode as shown in Figure 6. In this binding mode, instead of the two hydrogen bonds of d1 and d2, there are two other essential hydrogen bonds captured: G10:N2-H…FMN:O4, G10:N2-H…FMN:N5. The third deepest free-energy basin was located at d1 = 1.56 nm, d2 = 1.49 nm, and d = 0.77 nm, which referred to the binding mode with the isoalloxazine ring of the FMN base stacking between G10·U12·A25 triplet and G27 (Figure 7). Therefore, we can see that this ‘docking-like’ metadynamics simulation strategy can accurately recover the most stable binding mode of the FMN/RNA aptamer binding pocket system, meanwhile, provide other possible existing binding poses that are energetically favorable via different essential interactions with the same RNA binding pocket.

The metadynamics simulation also provided a potential dynamic pathway and mechanism of binding for the FMN/RNA aptamer complex. As shown in (Figure 4F) and (Movie S1), before the isoalloxazine ring of FMN intercalated between the G10·U12·A25 triplet and the G9·G27 mismatch, the FMN-recognizing nucleotide A26 formed A26·G9 mismatch with G9, G10 partly stacked in between U12·A25 Hoogsteen base-pairing and A26·G9 mismatch, and G27 in bulge state (Figure 4F Transition state B) (Figure 6). The intercalation of FMN pushed G10 to the G10·U12·A25 triplet state. Afterwards, A26 recognized the uracil-like edge of FMN, and G27 moved upwards with A26 and formed a G27·G9 mismatch with G9. Our molecular dynamics binding pathway with transition states is, to our knowledge, the first thermodynamic binding pathway at atomic resolution for this system. The previous study of the FMN binding mechanism of this system was only about the reduction potential shift on FMN caused by π-π and donor atom-π interactions of base stacking between the isoalloxazine ring of FMN and the G10·U12·A25 triplet above, G9·G27 mismatch below after binding to this RNA binding pocket [71].

**Figure 5:**
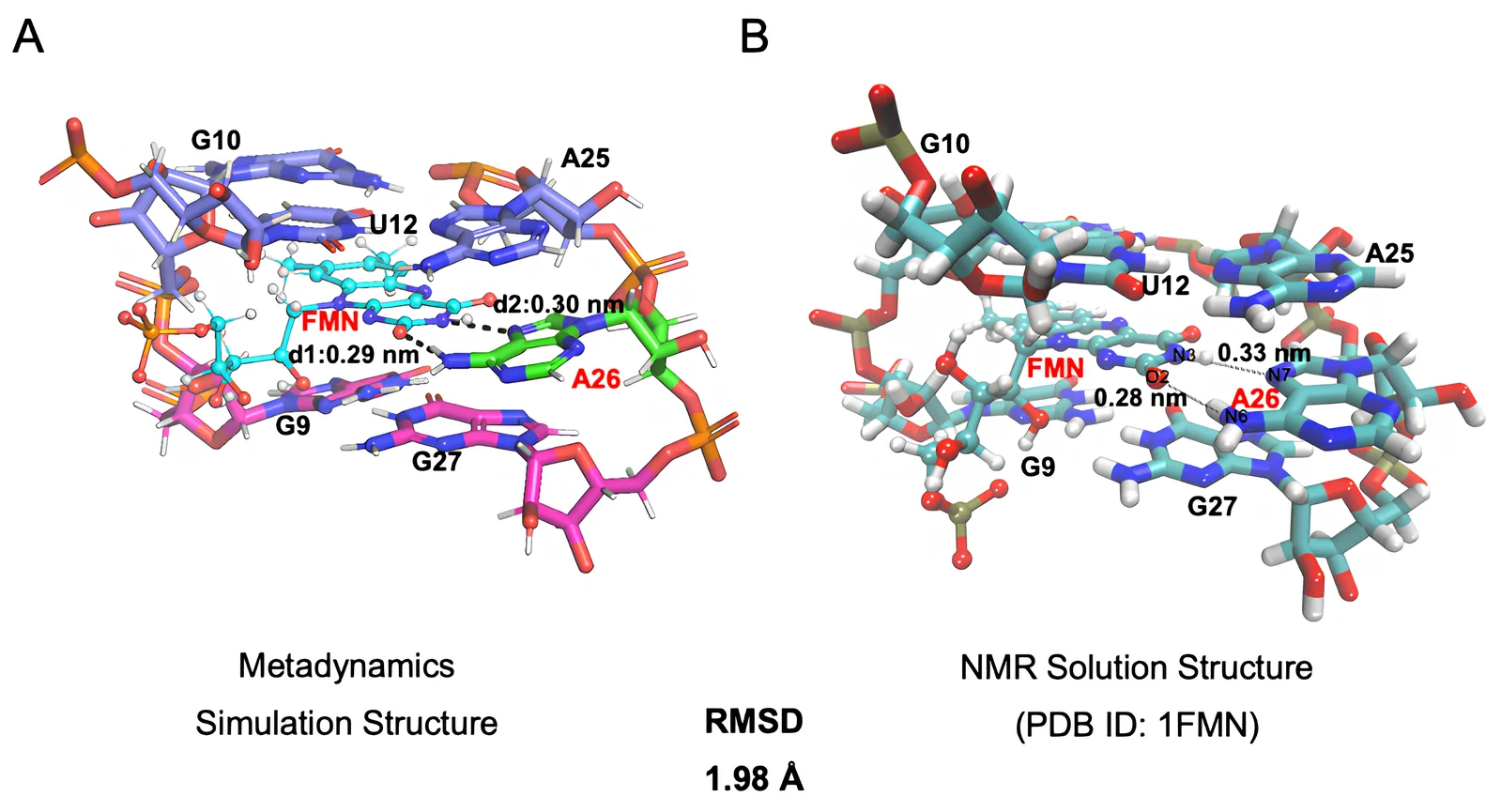
Comparison between metadynamics-recovering and NMR solution structure. (**A**) Snapshot of binding mode at the free-energy basin of the 2D free energy landscape onto d1 and d2 from metadynamics simulation; (**B**) Model 1 of NMR solution structures of FMN-FMN aptamer complex (PDB ID: 1FMN).

**Figure 6:**
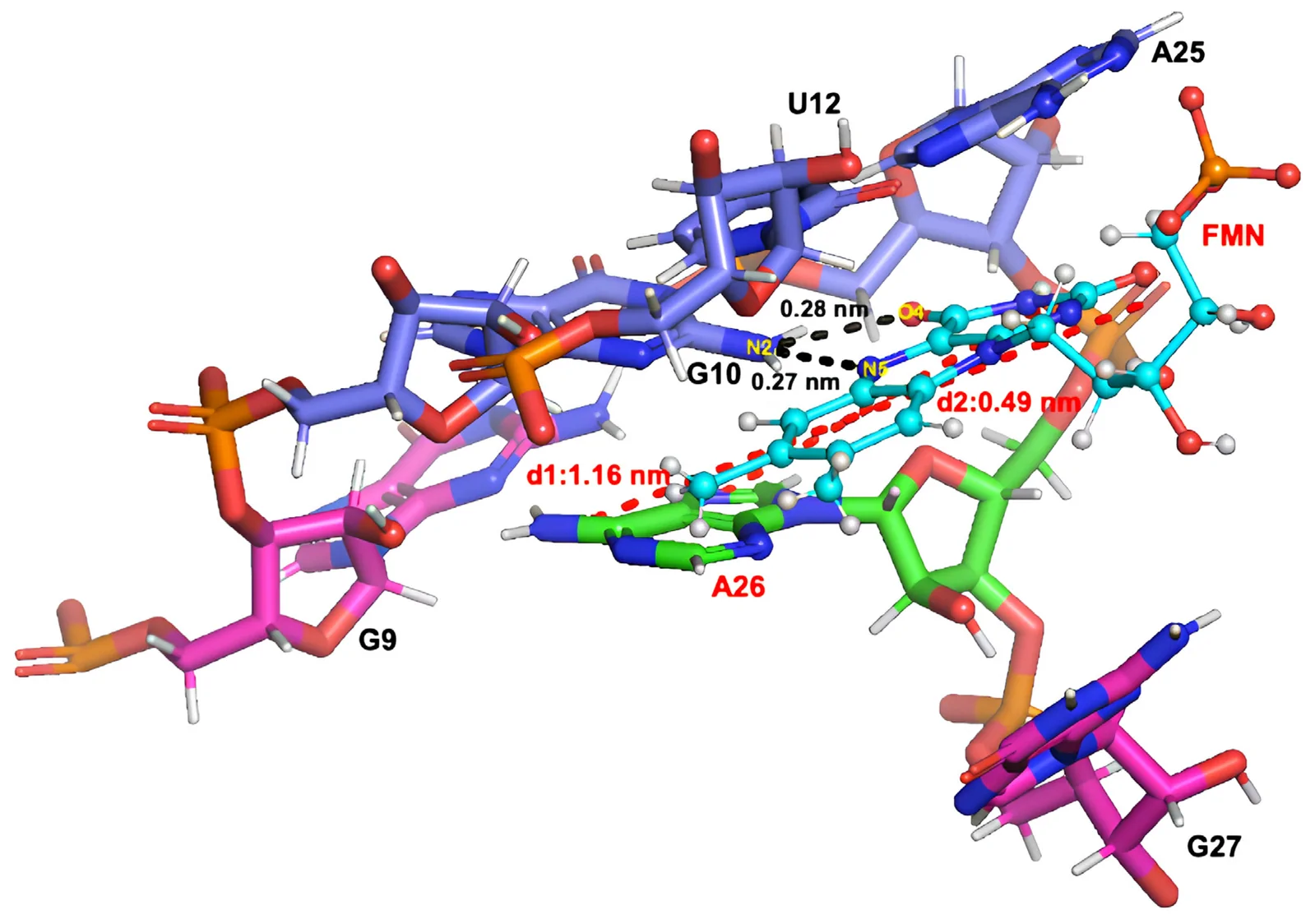
Snapshot of the binding mode at the second free-energy basin of the 2D free-energy landscape onto d1 and d2 from the metadynamics simulation.

**Figure 7:**
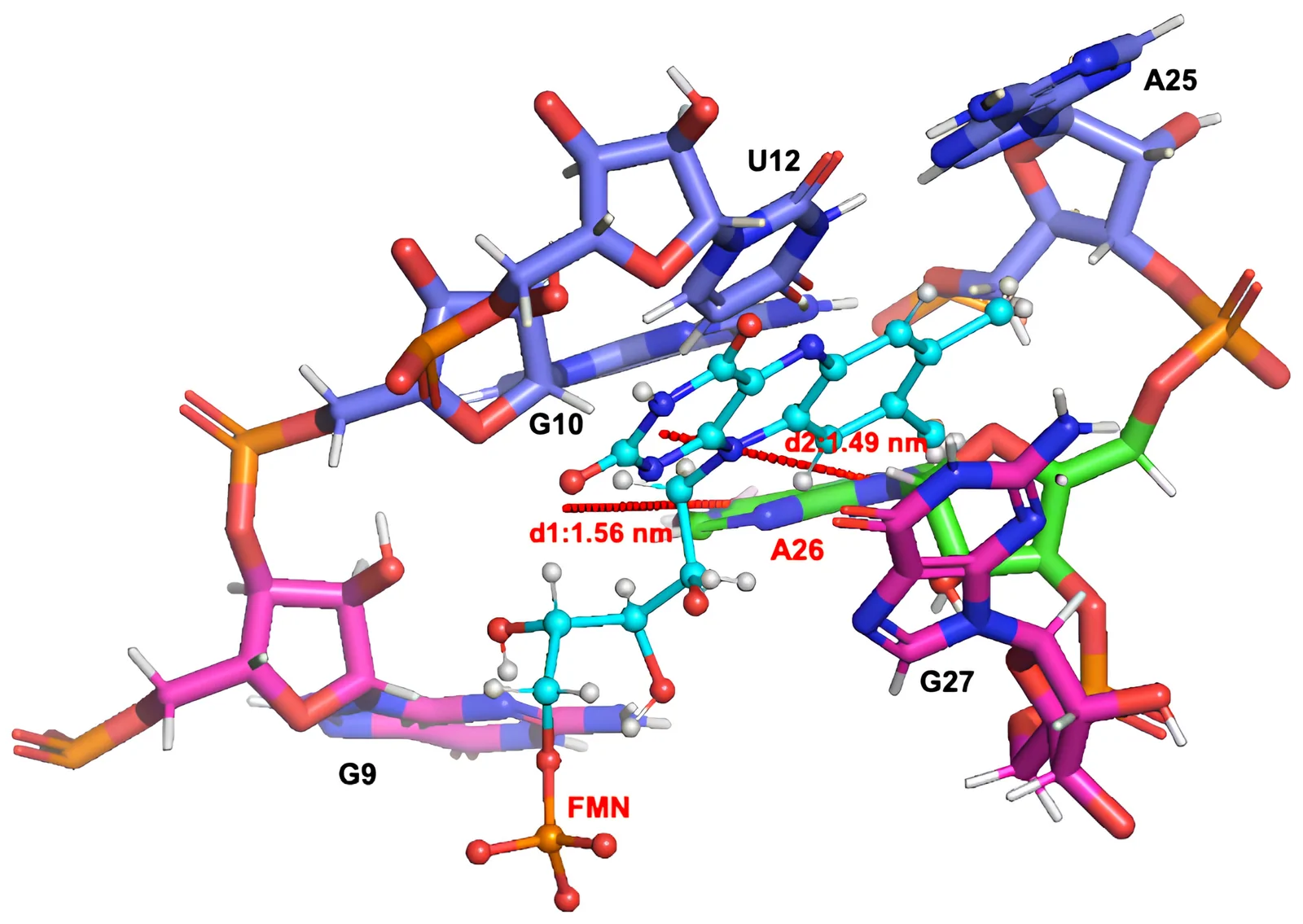
Snapshot of the binding mode at the third free-energy basin of the 2D free-energy landscape onto d1 and d2 from the metadynamics simulation.

### 3.2. Preq1 Riboswitch Aptamer

To test if this metadynamics simulation method can reproduce accurate binding mode recovery of a small-molecule ligand/RNA binding pocket complex, we chose to simulate the ligand of preQ1 binding to its riboswitch aptamer. In the solved crystal structure [5] of the preQ1 riboswitch aptamer in the metabolite-bound state (PDB ID: 3Q50), the Watson–Crick face of preQ1 formed G·C-like pairing with C15 by three hydrogen bonds of C15:N4-H…preQ1:O6, preQ1:N1-H…C15:N3, and preQ1:N2-H…C15:O2. The binding site of preQ1·C15 was stacked between the G5·C16 base pair and A14·G11·C7 triplet, which have been referred to as the “floor” and “ceiling” of the binding pocket (Figure 10B). Based on the same strategy of metadynamics we applied on FMN/RNA aptamer system, the COM distance between preQ1 ligand and riboswitch binding pocket was chosen as the first CVs (d of CVs), the distance from C15:N4 to preQ1:O6 was chosen as the second CVs (d1 of CVs) to represent the first core hydrogen bond of preQ1·C15 pairing, the distance from preQ1:N1 to C15:N3 was chosen as the third CVs to represent the second core hydrogen bond of preQ1·C15 pairing. The binding pocket is defined by the A14·G11·C7 triplet and G5·C16 base pair above and below the binding site.

As shown in Figure 8A and Movie S2, preQ1 started 1.8 nm away from the center of mass of the binding pocket, and the guanine base of preQ1 intercalated between the G5·C16 base pair and G11·C7 mismatch from the major groove direction, mainly driven by upper_walls restraints on d of CVs with 0.8 nm as the radius of the sphere. The preQ1·C15 pairing stabilized the ‘celling’ of the A14·G11·C7 triplet via base stacking force and stayed in this state for the remaining 32 ns without obvious changes in its center-of-mass location (Figure 8A). This dynamic process can explain why the Gaussian height pumped up and down between 0.3 and 0 kJ/mol in the first 8 ns and dropped to near zero afterwards without rebounding in the last 32 ns (Figure 8B). PreQ1 was powered by a Gaussian potential constructed on the three CVs to find an energy-favorable binding mode within the binding pocket in the first 8 ns. When preQ1 formed G·C-like pairing with C15, the Gaussian height decreased to near zero to keep preQ1 staying in this energy-favorable state.

The free energy surface of the metadynamics simulation shows a single free-energy basin at d1 = 0.29 nm, d2 = 0.29 nm, and d = 0.10 nm from both biased and reweighted calculations (Figure 9). The binding mode at the free-energy basin located by d1 = 0.29 nm, d2 = 0.29 nm has an RMSD of 0.87 Å from the crystal structure (resolution 2.75 Å), with core hydrogen bonds of d1 and d2 for preQ1·C15 pairing, A14·G11·C7 as the ceiling of the binding pocket, and C16·G5 as the binding pocket floor (Figure 10). The FES result once again proved that this metadynamics simulation strategy can recover binding modes of small-molecule ligand/RNA binding pocket complexes with high accuracy.

Moreover, the crystal structure of the preQ1 riboswitch aptamer in metabolite-free states [5] shows that C15 swinging out of the major groove and A14 shifting into the preQ1 binding site result in ‘close’ access to the preQ1 ligand. Our metadynamics simulation also captured the swinging of C15 during preQ1 recognition (Figure 9F) (Movie S2). The binding pathway and mechanism of the preQ1 ligand captured by our metadynamics simulation show that the minor groove side of preQ1 was recognized by bases of U6 and A29 first (Figure 9F Transition State), and preQ1 rotated in the plane of preQ1·C15 to adjust its angle toward the ligand-recognizing nucleotide C15. Meanwhile, C15 kept swinging in and out of the major groove, switching between C15 bulge state and base stacking state until preQ1 ‘read out’ the Watson-Crick edge of C15 at the 8th ns (Movie S2). The previous MD simulation study of this preQ1 riboswitch system by Hu and Zhou et al. [72] and Banáš et al. [73] correlated exceptionally well with our metadynamics binding pathway, which also captured hydrogen bonds between preQ1 and U6, A29. However, our metadynamics binding pathway is the first simulation movie showed that the amino group of preQ1 hydrogen-bonded with carbonyl oxygen of U6 base and amino nitrogen of A29 base firstly with C15 in bulge state as a transition state (Figure 9F) and read the final state of C15 pairing with Watson-Crick face of preQ1 as the deepest free-energy basin binding mode in the binding pathway two-dimensional free energy landscape projected onto d1 and d2, which shows the potential of metadynamics simulation for describing dynamic mechanism of molecular recognition.

**Figure 10:**
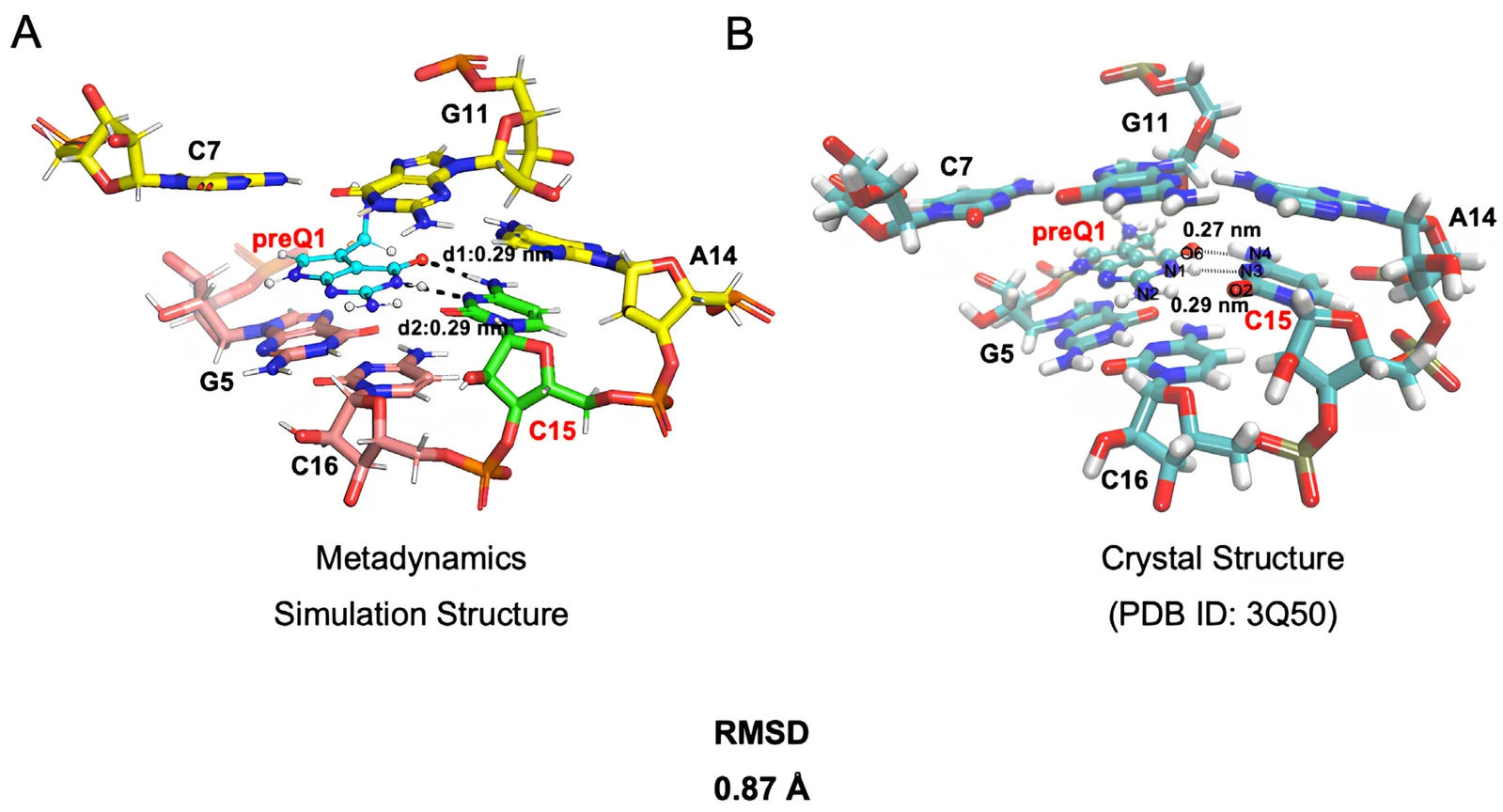
Comparison between metadynamics recovering and crystal structure. (**A**) Snapshot of binding mode at free-energy basin of 2D free energy landscape onto d1 and d2 from metadynamics simulation; (**B**) Crystal structure (resolution 2.75 Å) of preQ1 riboswitch aptamer complex (PDB ID: 3Q50).

### 3.3. Adenine Riboswitch Aptamer Domain

Another riboswitch studied was the adenine riboswitch aptamer domain, which is a 71-nucleotide RNA (rA71) of the translational control riboswitch from Vibrio vulnificus. The crystallography study showed that, in the final ligand-bound conformation, U74, as the ligand-recognition residue, formed Watson–Crick base pairing with the adenine ligand via two hydrogen bonds of ADE:N6-H…U74:O4 and U74:N3-H…ADE:N1. The binding site of ADE·U74 was base-stacked between two base-triples (Figure 13B) (PDB ID:5SWE) [6]. Therefore, the binding pocket of our metadynamics simulation was defined by these two triplets of C50·U75·A21 and A73·A52·U22 above and below the binding site. Based on the same collective variable setting strategy, there are still three CVs: the COM distance between the adenine ligand and the binding pocket (d of CVs), the distance between the hydrogen bond donor of ADE:N6 and the hydrogen bond acceptor of U74:O4 (d1 of CVs), and the distance between the hydrogen bond donor of U74:N3 and the hydrogen bond acceptor of ADE:N1 (d2 of CVs).

The radius of the sphere of upper_walls restraints acting on d of CV is 0.8 nm, which pushed adenine towards the major groove of the binding pocket from 1.9 nm away (Figure 11A). Due to the planar shape of the adenine ligand, it intercalated between C50·U75·A21 and A73·A52·U22 easily. Afterwards, within the range of d less than 0.8 nm, adenine occupied different positions relative to U74, which was powered by a Gaussian potential on the three CVs. Under the effect of a Gaussian height of 0.8 kJ/mol with a bias factor of 4, adenine rotated and moved in the plane of adenine·U74 until forming A·U-like pairing with U74 in the 10th ns; meanwhile, U74 switched between bulge state and base stacking state (Figure 12F) and (Movie S3). Afterwards, the Gaussian height gradually decreased to near zero (Figure 11B).

The recognition between the adenine ligand and U74 stabilized the whole binding pocket and was read as the most energy-favorable conformation in the free energy surface. As shown in Figure 12, the main free-energy basin is located at d1 = 0.29 nm, d2 = 0.30 nm, and d = 0.23 nm in both biased and unbiased (reweighted) FES, which points to the state of adenine·U74 pairing as shown in Figure 13A. This binding mode of adenine recovered by metadynamics has an RMSD of 0.87Å from the crystal structure (resolution 3.00 Å) (Figure 13B), which proves the accuracy of the metadynamics method again. A previous isothermal titration calorimetry (ITC) study by Gilbert et al. [74] demonstrated clearly that the pyrimidine at position 74 is the sole determinant of purine riboswitch specificity, which is also in agreement with our metadynamics-FES recovery.

Moreover, the binding pathway of the adenine ligand in our metadynamics simulation movie showed that the intercalation of the adenine ligand holds U74 back to the base-stacking state from the U74 bulge state firstly, which further stabilized the two triplets of C50·U75·A21 and A73·A52·U22 above and below the binding site (Movie S3). In the X-ray crystallography study of this system, the binding pocket with adenine·U74 pairing in the middle is at the three-way junction of the adenine riboswitch, and the binding of the adenine ligand locked P1 and J2/3 together by the formation of the two triplets of C50·U75·A21 and A73·A52·U22 above and below, which were absent in the riboswitch apo structure [6]. A previous NMR study by Noeske et al. [75] and an MD simulation study by Sharma et al. [76] also clarified the absence of two triplets of C50·U75·A21 and A73·A52·U22 in the apo RNA structure, which two could be held together by adenine·U74 pairing in the holo state. However, our metadynamics simulation is the first study to capture the swinging out of U74 to the minor groove as a transition state (Figure 12F Transition State) and elucidate this adenine binding mechanism by thermodynamically simulating the whole binding process with all binding details (Movie S3) instead of only comparing apo and holo states, which further proved the enhanced power of metadynamics compared with plain MD.

**Figure 13:**
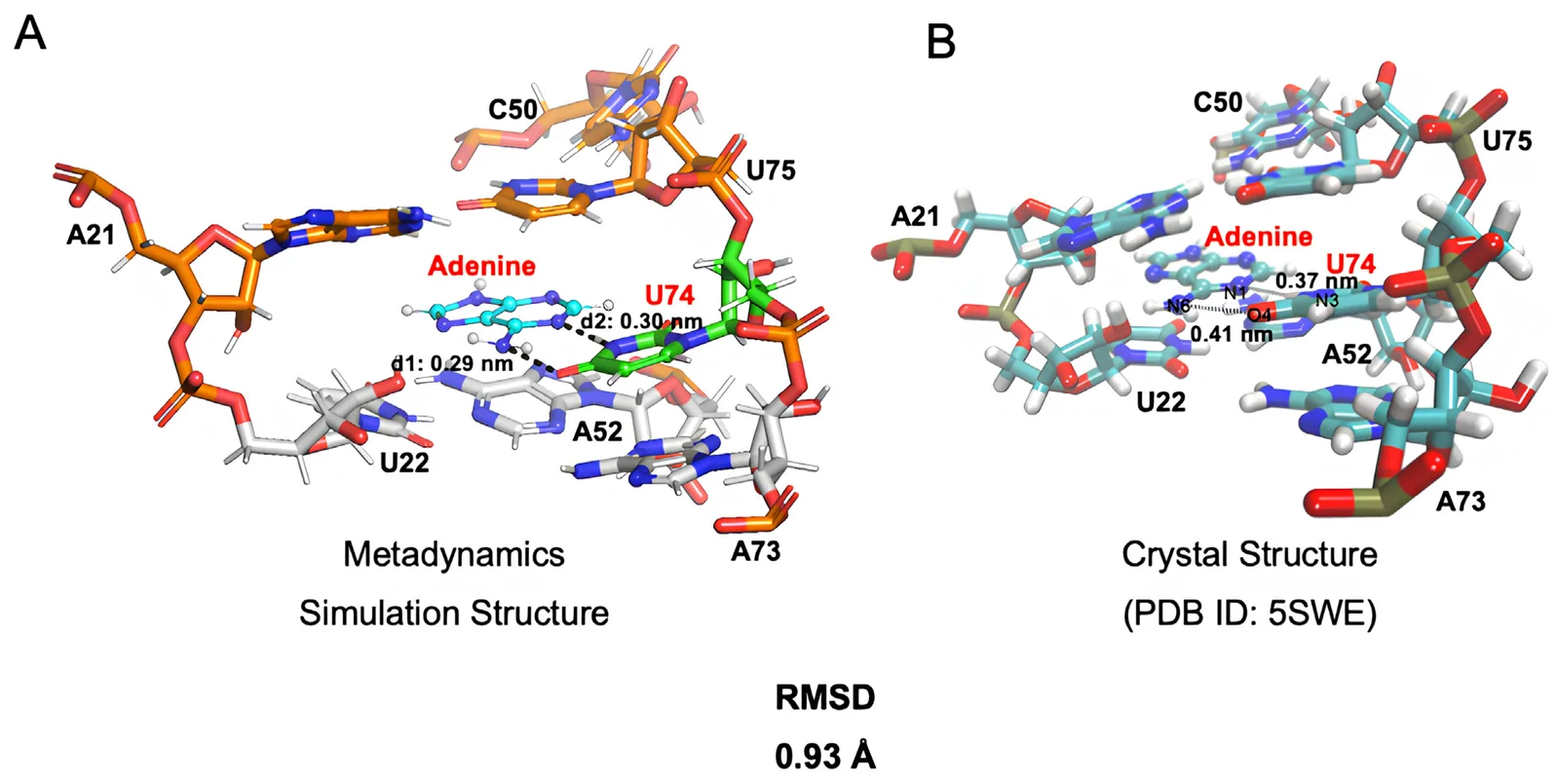
Comparison between metadynamics recovery and crystal structure. (**A**) Snapshot of the binding mode at the free-energy basin of the 2D free-energy landscape onto d1 and d2 from metadynamics simulation; (**B**) Crystal structure (resolution 3.00 Å) of the adenine riboswitch aptamer domain complex (PDB ID: 5SWE).

### 3.4. Citrulline/RNA Aptamer

The fourth small molecule ligand-RNA system studied is the citrulline/RNA aptamer complex obtained from in vitro selection. The initial structure of metadynamics is from an NMR solution structure (PDB ID:1KOD) [44]. The binding pocket of metadynamics is defined the same as in the previous NMR spectroscopy study by G3-G4-U5-A7-G9-G25-G28-U29, G9-G28-G25, which form an uneven plane at the bottom, and G4-U5 nearly perpendicular to that surface (Figure 16B). The solved NMR solution structure showed that sequence-specific interactions occurred between the urea group of the citrulline ligand and the Watson–Crick edge of U29 and the Hoogsteen edge of G4 (Figure 16B). Therefore, we picked two core hydrogen bond distances of U29:N3-H…CIR:O7 and CIR:N8-H…G4:N7 as d1 of CVs and d2 of CVs, respectively. d of CVs is still the COM distance between the citrulline ligand and the RNA binding pocket, which functioned as the main driving force of binding/unbinding. In this case, a rigid structure of the RNA binding pocket was needed for the recognition of the citrulline ligand. Therefore, moderate-intensity position restraints (500 kJ mol^−1^ nm^−2^) on the RNA binding pocket were added for the simulation. The original NMR study demonstrated that small RNAs are able to fold into compact structures, thereby bringing various functional groups of nucleotides scattered along the structure of the small-molecule ligand together, and revealed that the variability in specificity of an in vitro-selected RNA aptamer is achieved by mutations of a subset of nucleotides and leaving invariant those that mold the recognition scaffold as in protein enzymes [44], which also supported that the binding of citrulline to its RNA aptamer requires a specific conformation of the RNA binding pocket.

As shown in (Figure 14A) and (Movie S4), within the range of d less than 0.9 nm limited by upper_walls restraints, citrulline bound to its RNA binding pocket from the minor groove direction and experienced plenty of binding modes inside the RNA binding pocket in a 100 ns-long well-tempered docking metadynamics simulation. In this case, after optimization of the bias factor, we found that under a Gaussian height of 0.6 kJ/mol, a higher bias factor of 100 was needed to achieve convergence of the metadynamics simulation, which means 100 times the system temperature is needed to cross relevant free-energy barriers and fill up relevant free-energy basins efficiently in this simulation. Under the action of a Gaussian height of 0.6 kJ/mol with a bias factor of 100, citrulline realized relatively high-frequency state transitions, which effectively pushed citrulline away from energy-unfavorable binding modes until the free-energy basin conformation was met (Figure 14B).

In this case, a relatively wide free-energy basin was found in the free energy surface (Figure 15D,E). The most energy-favorable binding mode of citrulline is located at d1 = 0.26 nm, d2 = 0.27 nm, and d = 0.26 nm in both biased and reweighted FES (Figure 15A–C), which proved the importance of hydrogen bonding interactions between the urea group of citrulline and the Watson–Crick edge of U29 and the Hoogsteen edge of G4 for the recognition between citrulline and its RNA binding pocket. As shown in Figure 16, the binding mode of citrulline at the free-energy basin of metadynamics has an RMSD of 0.86 Å from the NMR solution structure, which is another successful case of small molecule-RNA binding mode recovery by our metadynamics method.

**Figure 16:**
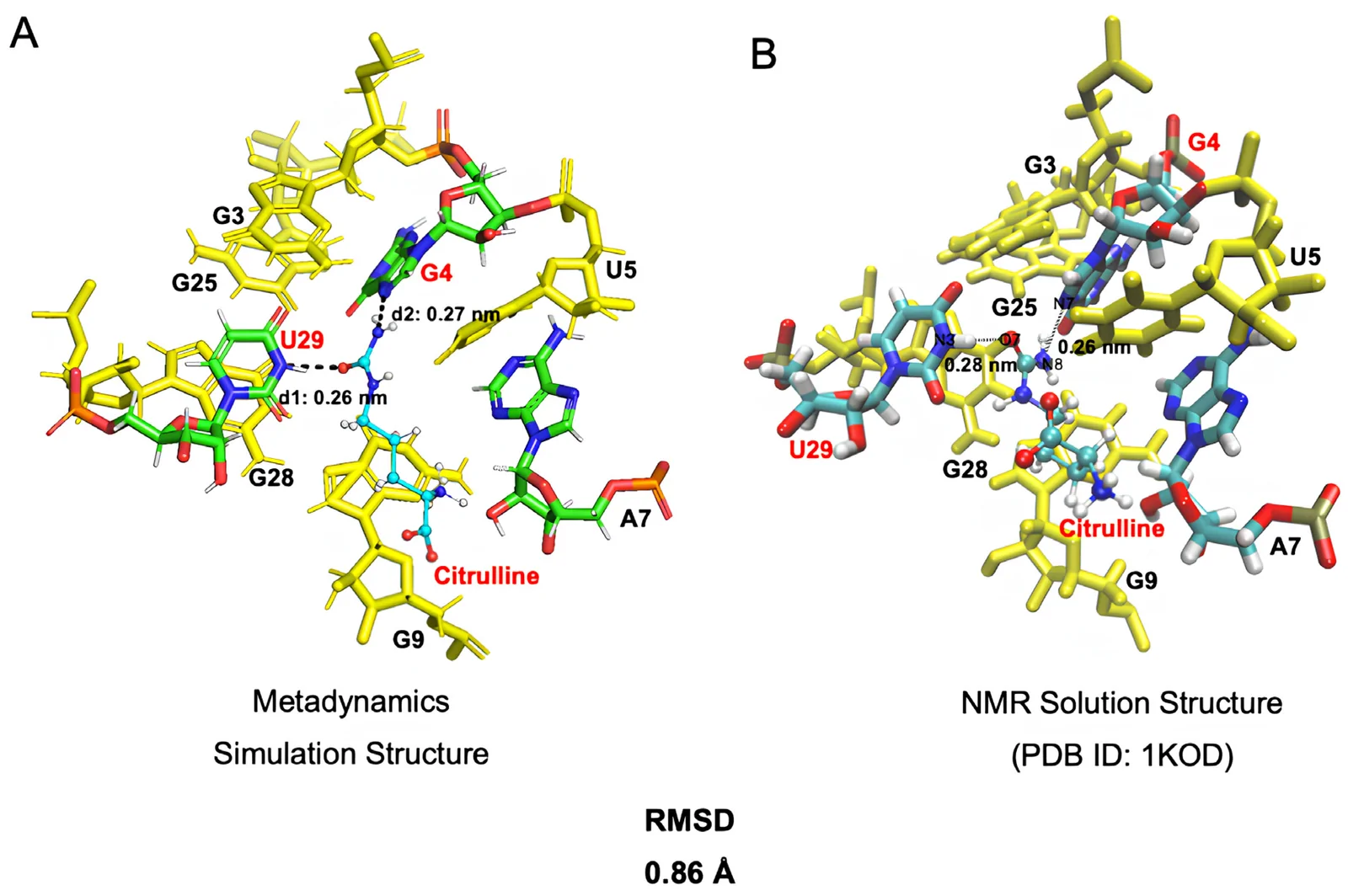
Comparison between metadynamics-recovered and NMR solution structure. (**A**) Snapshot of binding mode at the free-energy basin of the 2D free energy landscape onto d1 and d2 from the metadynamics simulation; (**B**) NMR solution structure of citrulline RNA aptamer complex (PDB ID: 1KOD).

### 3.5. Arginine/RNA Aptamer

The last small molecule ligand-RNA system studied is the arginine/RNA aptamer complex (PDB ID: 1KOC), which was evolved from the citrulline/RNA aptamer illustrated above. The arginine RNA aptamer differs from the citrulline aptamer at only three nucleotides out of 44, at A3, G5, and C29. NMR solution structure showed that the conformation of the arginine-binding pocket is very similar to the citrulline RNA-binding pocket, with an uneven bottom plane of G9-G28-G25 and G4-G5 perpendicular to this surface. Therefore, we defined the arginine-binding pocket of the metadynamics simulation by the same positions of A3-G4-G5-A7-G9-G25-G28-C29. For the cognate amino acid arginine, the hydrogen-bonding interactions occurred between the guanidino group of the arginine ligand and the Watson–Crick edge and the C29 Hoogsteen edge of G4 (Figure 19B). Thus, we chose the hydrogen bond donor-to-acceptor distance of ARG:NH2-H…C29:N3 as d1 of CVs, ARG:NH2-H…G4:O6 as d2 of CVs, and the COM distance between arginine and the RNA binding pocket as d of CVs. Same as the previous case of the citrulline/RNA aptamer system, position restraints (500 kJ mol^−1^ nm^−2^) on RNA were added to keep the conformation of the RNA binding pocket.

In the metadynamics simulation, upper_walls restrain forced arginine towards the minor groove of the binding pocket from 2.5 nm away (Figure 17A). Within the range of d less than 0.7 nm, arginine has the freedom to move and rotate without being limited by restraints. The Gaussian height of 0.6 kJ/mol with the bias factor of 6.0 on the three CVs gave arginine sufficient energy for overcoming energy barriers of state transition and filling up free-energy basins of binding modes so that arginine experienced as many binding modes as possible within 60 ns (Movie S5). Whenever arginine approaches an energy-favorable binding mode, the height of the Gaussian decreased to keep arginine staying in this state for longer. Whenever arginine approaches an energy-unfavorable binding mode, the height of the Gaussian increased to push arginine out of this state. Until the most energy-favorable conformation was found, the Gaussian height dropped to near zero without rebounding, as shown in Figure 17B.

According to the free energy surface of metadynamics (Figure 18), the deepest free-energy basin was located at d1 = 0.33 nm, d2 = 0.31 nm, and d = 6 nm from both biased and reweighted calculations, which referred to the conformation of arginine at the end of the simulation when the two essential hydrogen bonds of d1 and d2 formed at the same time (Figure 19A). As shown in Figure 19, the most energy-favorable binding mode of arginine has an RMSD of 0.64 Å from the NMR solution structure, which shows that the metadynamics simulation recovery result is still consistent with the experimental result. Moreover, the second energy-favorable binding mode was found at d1 = 0.97 nm, d2 = 0.87 nm. In this binding mode (Figure 20), instead of the two hydrogen bonds of d1 and d2, we captured hydrogen bonding interactions between the guanidino group of arginine and the phosphodiester backbone of RNA, and the amino group of arginine and the Watson–Crick edge of C29.

**Figure 18:**
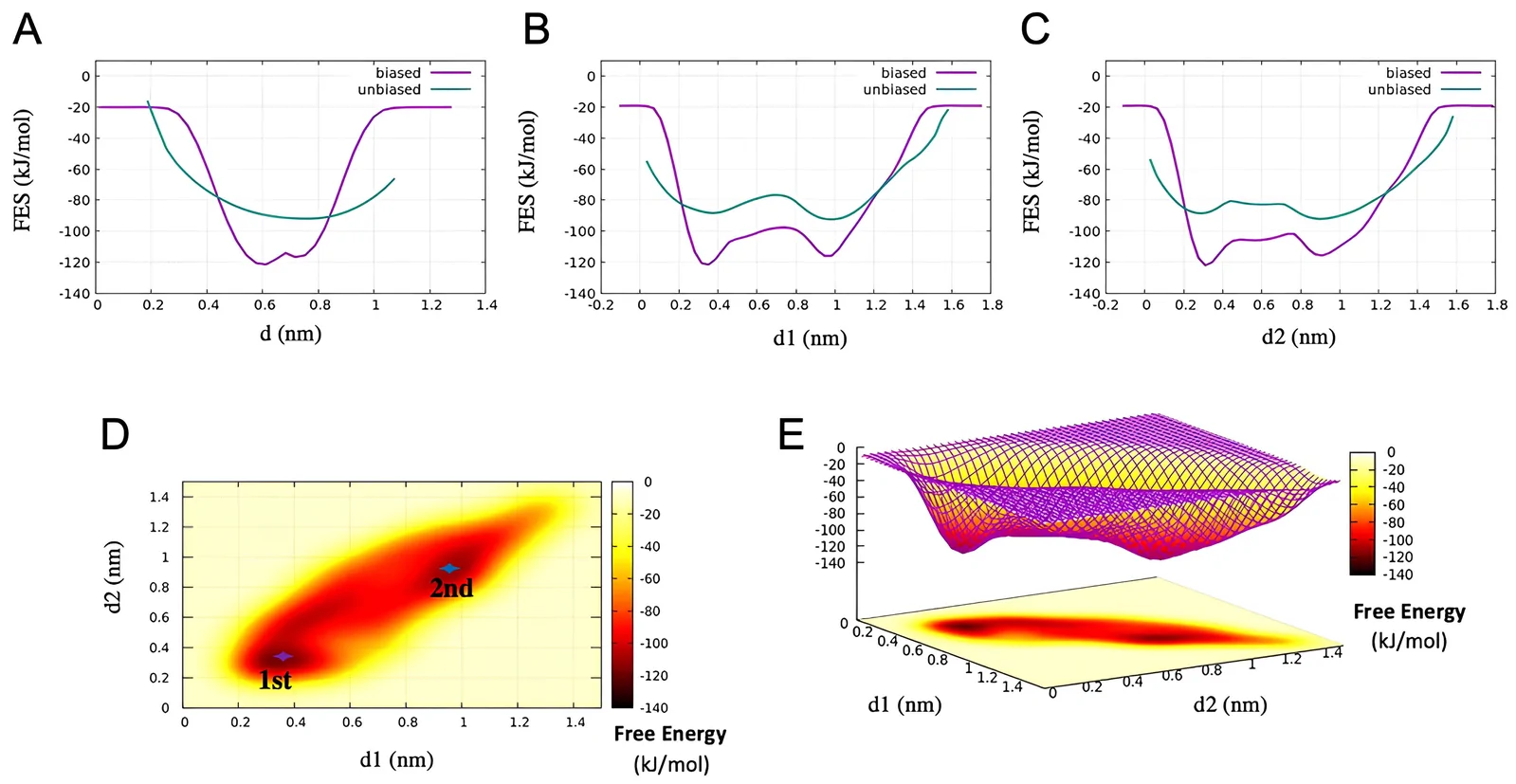
Comparison of the binding FES calculated from the metadynamics bias potential between biased (in purple) and unbiased (by reweighting, in green) as a function of (**A**) d of CVs (COM distance between arginine and binding pocket); (**B**) d1 of CVs (the distance between ARG:NH2 and C29:N3); (**C**) d2 of CVs (the distance between ARG:NH2 and G4:O6). Two-dimensional free energy landscape onto d1 and d2 (**D**) presented in 2D; (**E**) presented in 3D.

**Figure 19:**
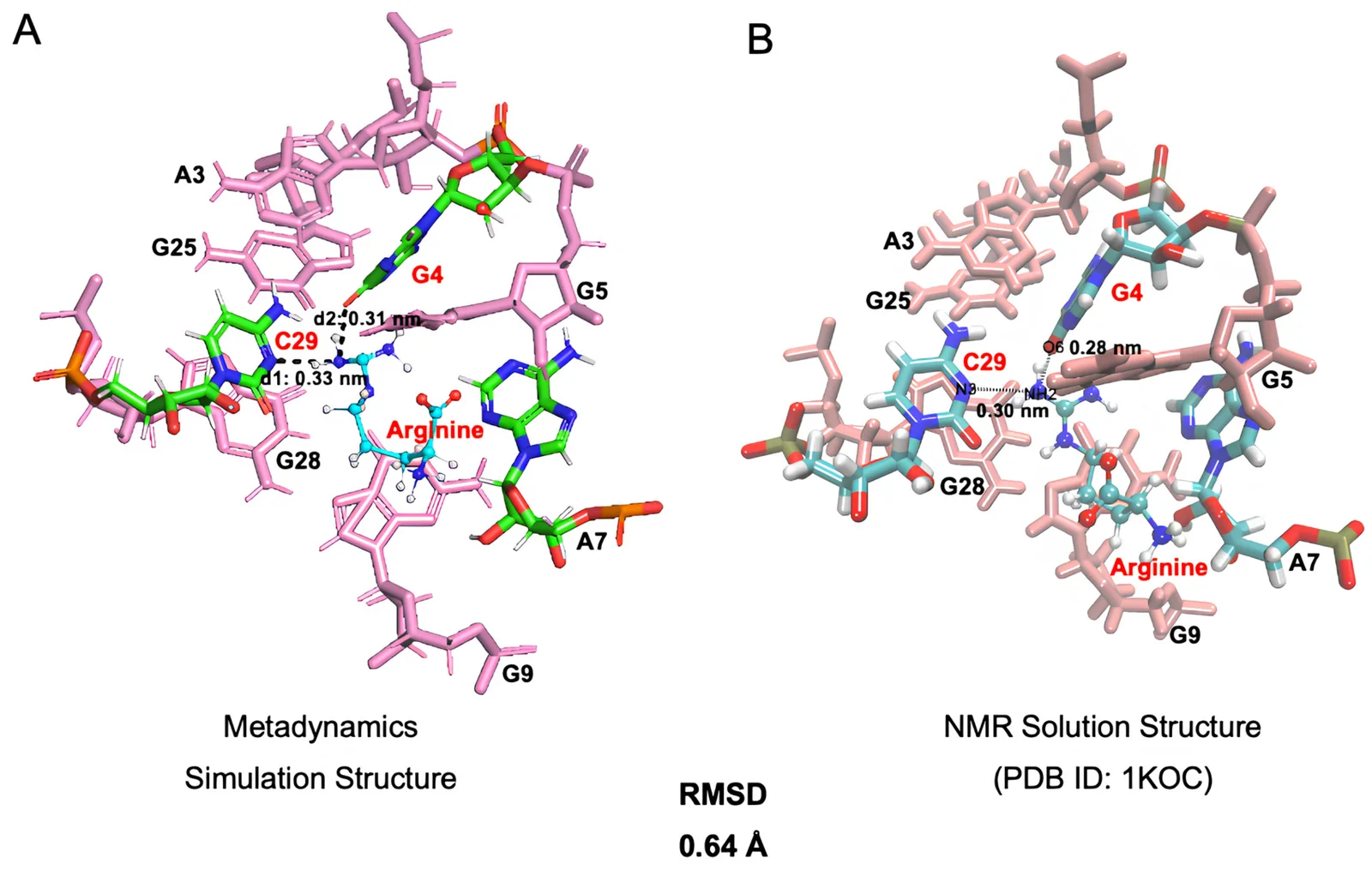
Comparison between metadynamics-recovered and NMR solution structure. (**A**) Snapshot of the binding mode at the free-energy basin of the 2D free-energy landscape onto d1 and d2 from the metadynamics simulation; (**B**) NMR solution structure of the arginine-RNA aptamer complex (PDB ID: 1KOC).

**Figure 20:**
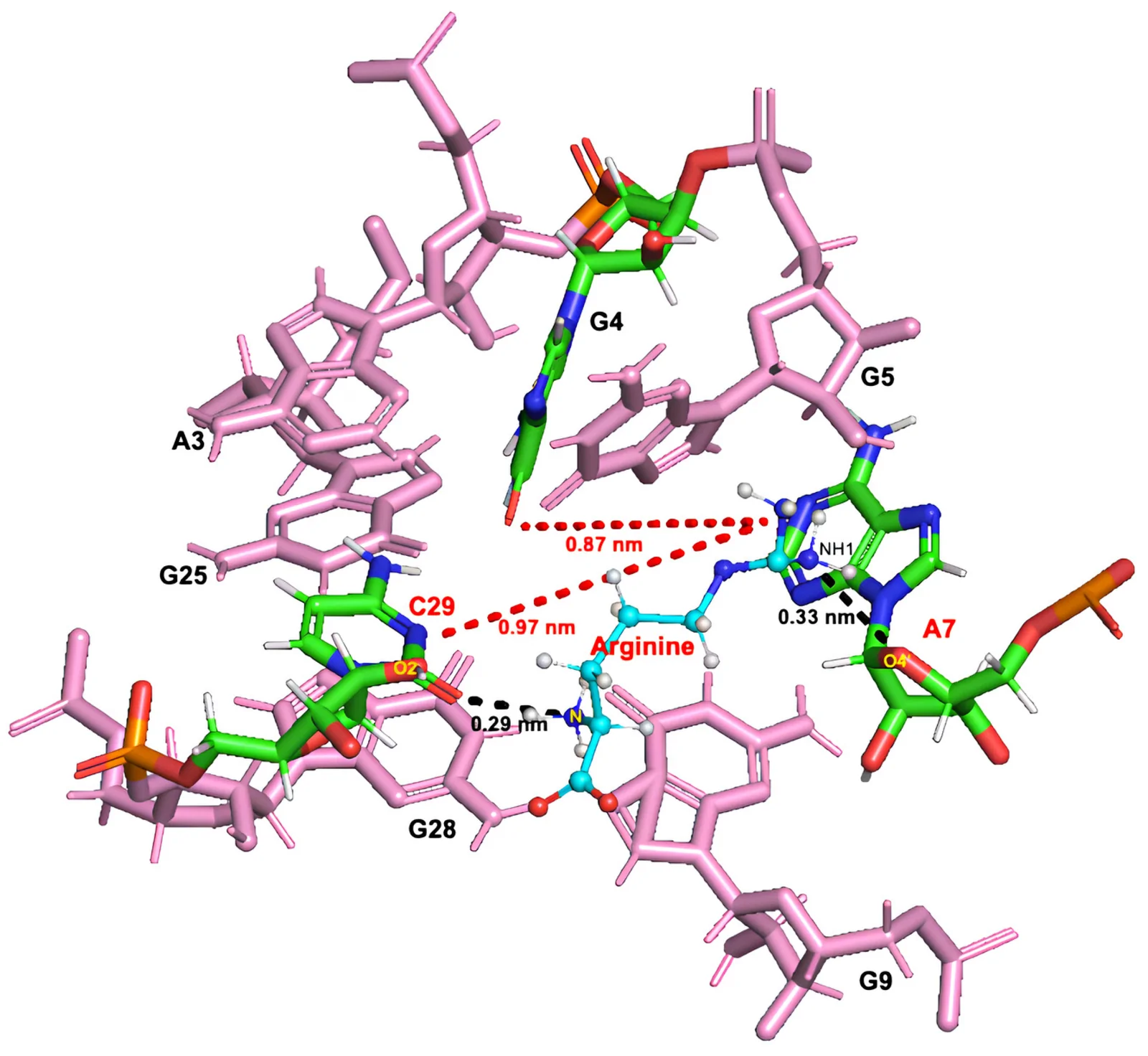
Snapshot of the binding mode at the second free-energy basin of the 2D free-energy landscape onto d1 and d2 from the metadynamics simulation.

## 4. Conclusions

Elucidating the mechanism of the molecular recognition process between small-molecule ligands and their RNA binding pocket is always a key concern in drug discovery. MD simulation has been an essential tool in interpreting structural dynamics of RNA and how they bind with small-molecule ligands at atomic resolution. However, conventional unbiased MD simulations may miss many states with nonergodic dynamics due to the insurmountable high energy barriers in the binding pathway. To address this limitation, we applied well-tempered metadynamics coupled with upper_walls restraints in this work, thus introducing a novel sampling approach for recovering binding modes of small-molecule ligands recognizing the RNA binding pocket. The two-dimensional free energy landscape of the whole binding process can be reconstructed in this method by incorporating a couple of key hydrogen-bonding interactions between the small-molecule ligand and its RNA binding pocket, which can sample multiple transition states during the binding process and finally probe the most energy-favorable binding modes accurately.

We calculated the free energy landscape as a function of three collective variables: the COM distance between the small-molecule ligand and its RNA binding pocket, the hydrogen bond donor-to-acceptor distances of two hydrogen bonds between the small-molecule ligand and its RNA binding pocket, which comprise the three coordinates of the resulting free energy surface. By filling up free-energy basins of different binding modes in the binding pathway with Gaussian potentials, the Gaussians on the three CVs gave the system enough potential to mimic all possible dynamic motion and rotation of the small-molecule ligand during the binding process that cannot be reached by unbiased MD. The upper_walls bias effectively improved the ergodicity of metadynamics docking and the convergence of the free energy surface, avoiding invalid sampling. We studied five cases to exemplify the validity of the methodology: flavin mononucleotide (FMN) binding with 35-nucleotide RNA aptamer identified through in vitro selection, preQ1 riboswitch aptamer recognized by its metabolite, ligand binding to adenine riboswitch aptamer domain, RNA aptamer complexed with citrulline, and RNA aptamer complexed with arginine. Our simulation recovery of the binding mode at the deepest free-energy basin in each case is in good agreement (RMSD below 2.0 Å) with structures solved by X-ray crystallography or NMR. More importantly, we presented a possible molecular dynamics binding pathway and binding mechanism for each of the first three systems, FMN/RNA aptamer, preQ1/riboswitch aptamer, and adenine/riboswitch aptamer domain, the whole induced-fit binding process of which was captured in this metadynamics study with multiple transition states probed by multiple free-energy basins. For the last two cases, citrulline/RNA aptamer and arginine/RNA aptamer, moderate-intensity position restraint on the RNA binding pocket was needed in the metadynamics simulation to recover the best binding mode, which made the modeling a docking process (instead of a binding pathway) with ligand switching binding modes in a relatively rigid RNA binding pocket powered by a Gaussian potential added to the three CVs.

The good agreement of our computational recovery with experimentally solved geometry and the molecular dynamics insights presented in this work demonstrates that metadynamics can be applied to effectively sample state transitions in the ligand-binding process and accurately recover binding modes via suitable selection of collective variables, which we have benchmarked with structures solved by X-ray crystallography or NMR. By coupling with upper_walls restraints, we have enabled fast free energy profile calculation of the small-molecule binding process. In general, our simulation strategy will enable a faster and more detailed RNA-ligand binding investigation, facilitating a direct comparison of molecular modeling to experimental measurements. Moreover, for unknown binding modes of any small-molecule ligand-RNA systems, our approach has the potential to be further optimized for simulating the whole binding path dynamically and accurately predicting the tertiary structure of the binding complex by only coupling hydrogen-bond information of binding via NMR or chemical probing, which will offer a valuable design and characterization tool for the growing community interested in applying in silico prediction to drug development of RNA-targeted small-molecule compounds.

## Figures and Tables

**Figure 1:**
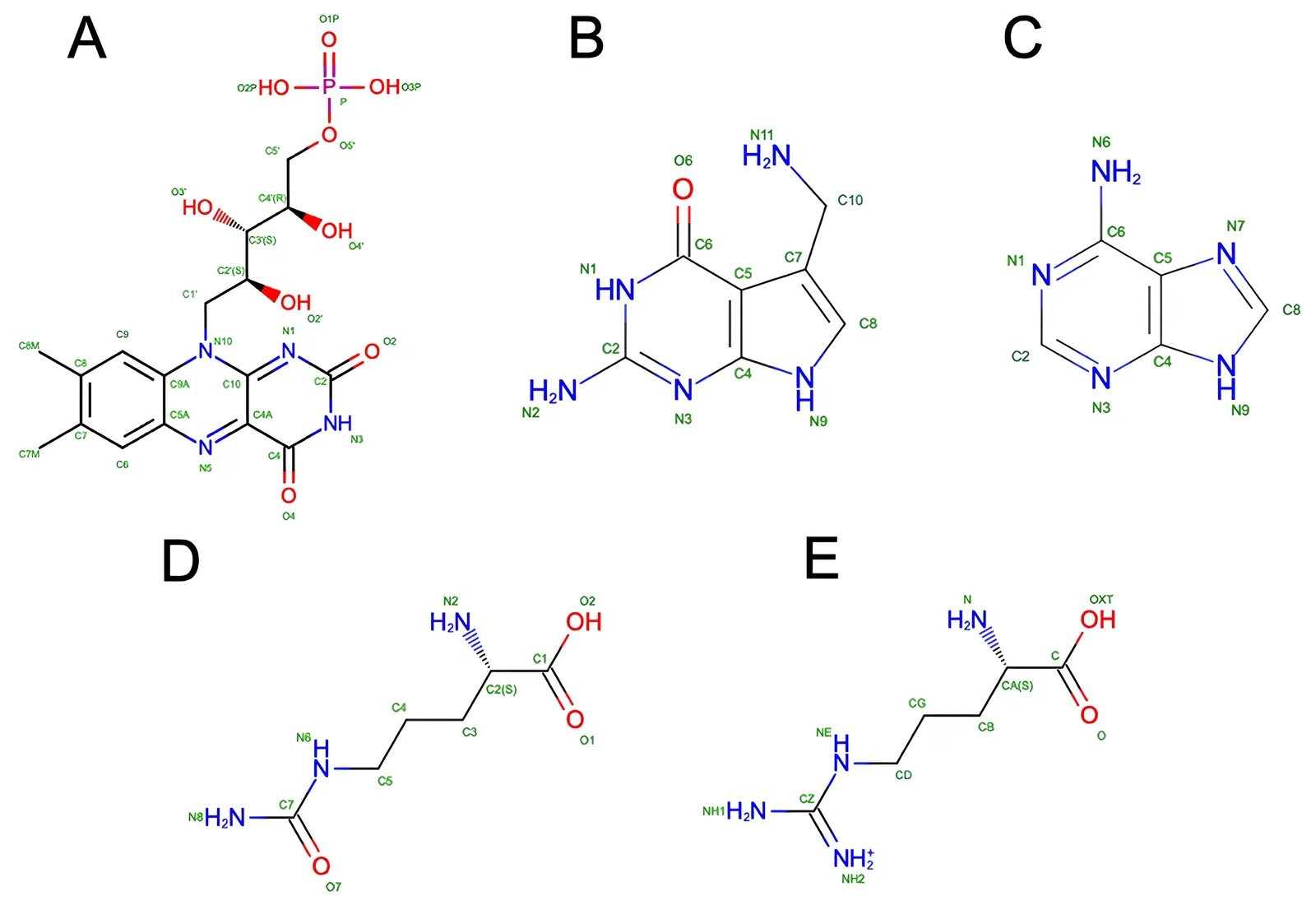
Small molecule ligand chemical structure of (**A**) flavin mononucleotide (FMN); (**B**) PreQ1; (**C**) adenine; (**D**) citrulline and (**E**) arginine.

**Figure 2:**
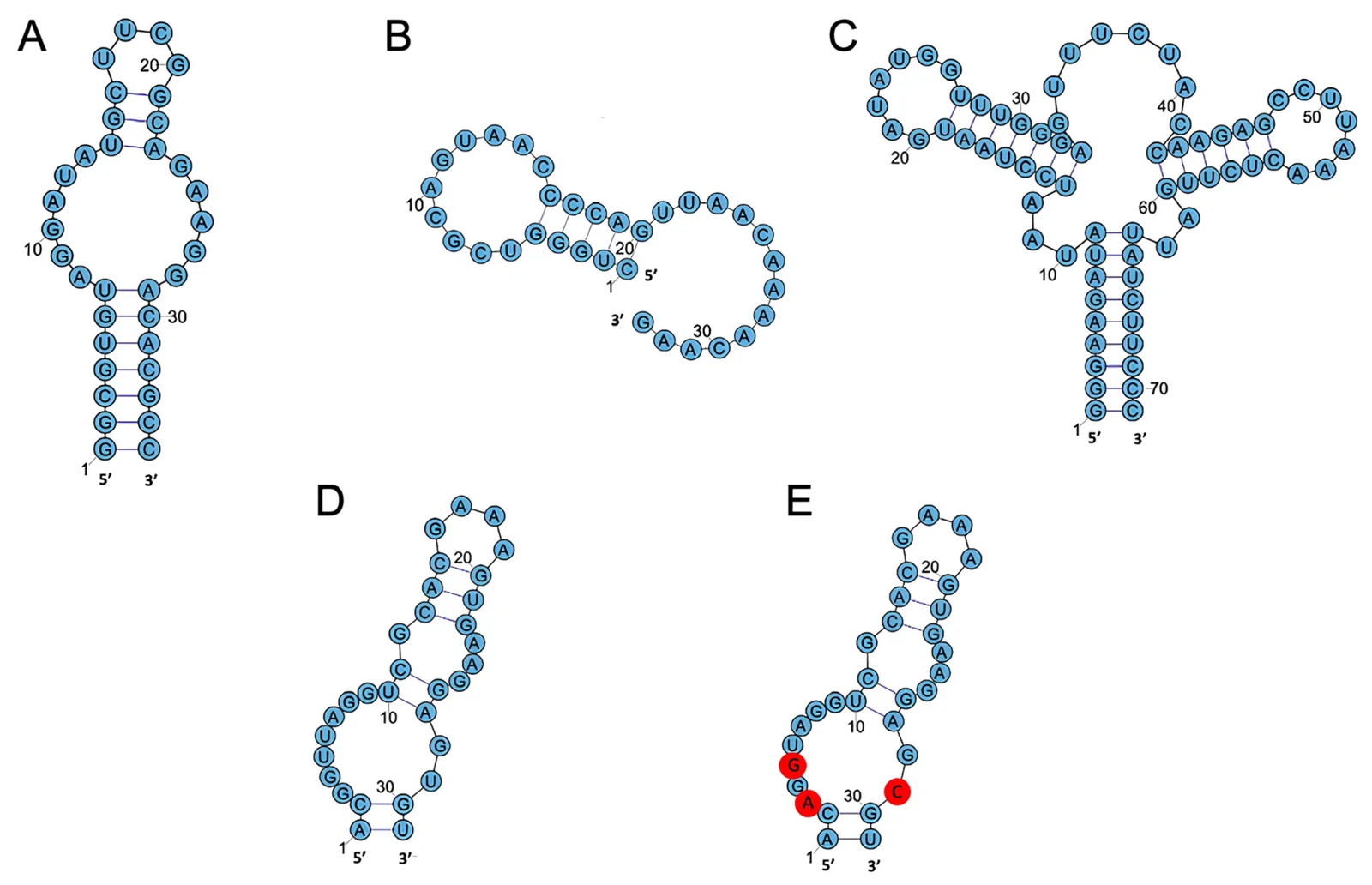
RNA secondary structure of (**A**) FMN aptamer; (**B**) preQ1 riboswitch aptamer; (**C**) adenine riboswitch aptamer; (**D**) citrulline aptamer; and (**E**) arginine aptamer that evolved from (**D**) citrulline aptamer by three nucleotide mutations at G3, U5, and U29 to A3, G5, and C29 as colored in red.

**Figure 3:**
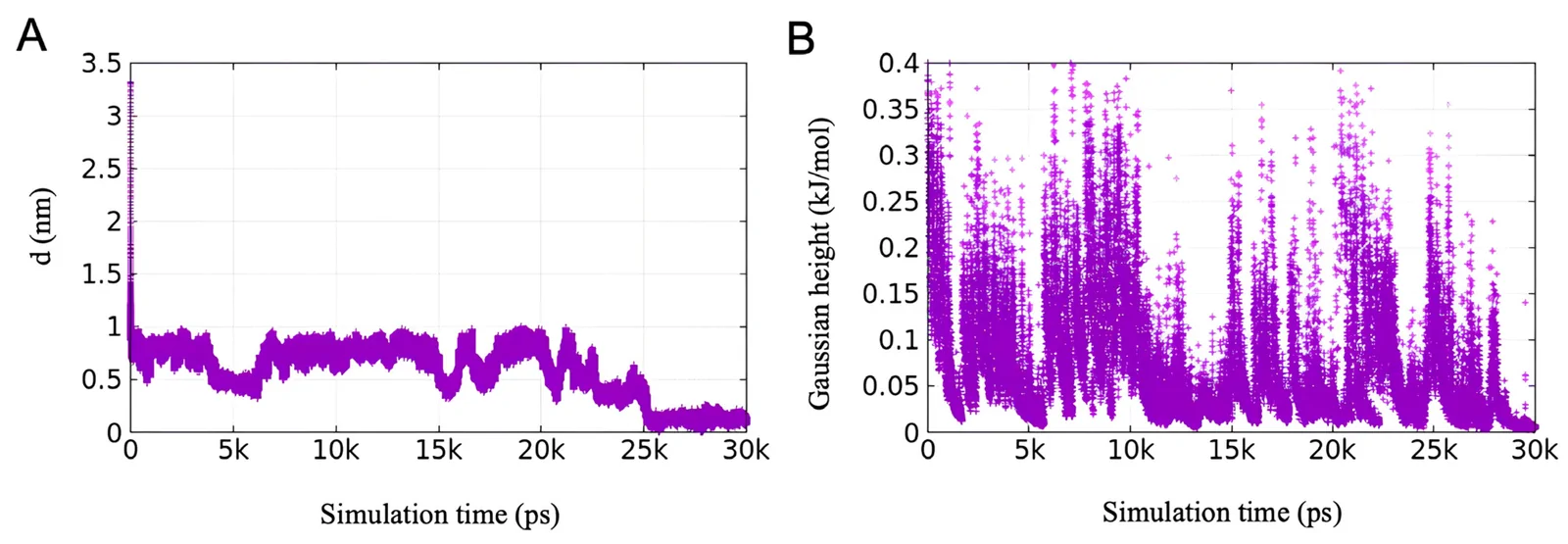
Time evolution of (**A**) d of CVs (COM distance between FMN and RNA binding pocket defined by G10·U12·A25 triplet and G9·G27 mismatch above and below FMN binding site) and (**B**) Gaussian height in a 30ns-long well-tempered metadynamics simulation of the FMN/RNA aptamer system.

**Figure 4:**
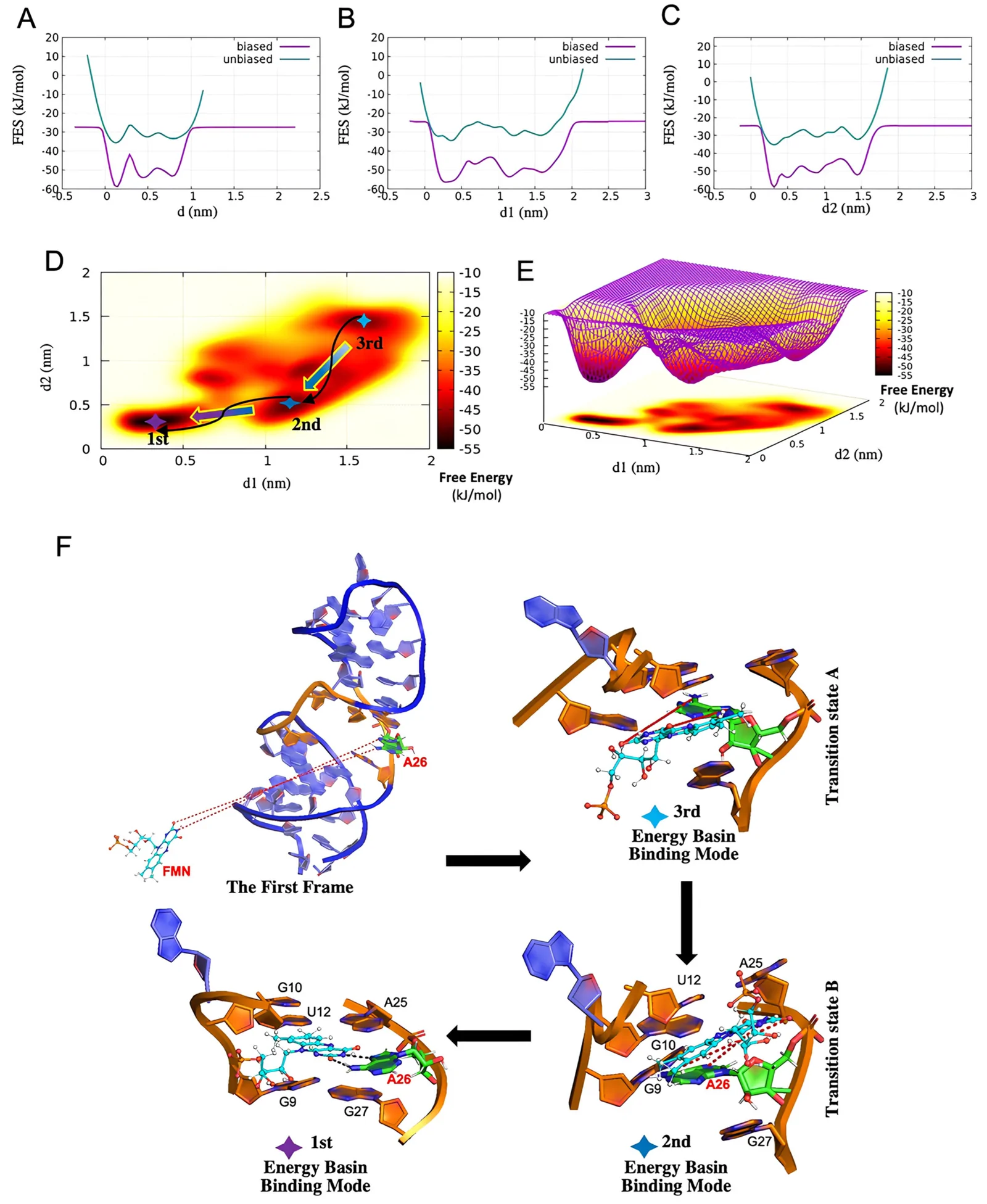
Comparison of the binding FES calculated from the metadynamics bias potential between biased (in purple) and unbiased (by reweighting, in green) as a function of (**A**) d of CVs (COM distance between FMN and binding pocket); (**B**) d1 of CVs (the distance between hydrogen bond acceptor FMN:O2 and hydrogen bond donor A26:N6); (**C**) d2 of CVs (the distance between hydrogen-bound donor FMN:N3 and hydrogen-bound acceptor A26:N7). Two-dimensional free-energy landscape projected onto d1 and d2 (**D**) presented in 2D; (**E**) presented in 3D; (**F**) Binding pathway and mechanism. In transition state B, FMN binding nucleotide A26 formed an A26·G9 mismatch with G9; G10 partly stacked between U12·A25 and the A26·G9 mismatch; G27 was in a bulge state.

**Figure 8:**
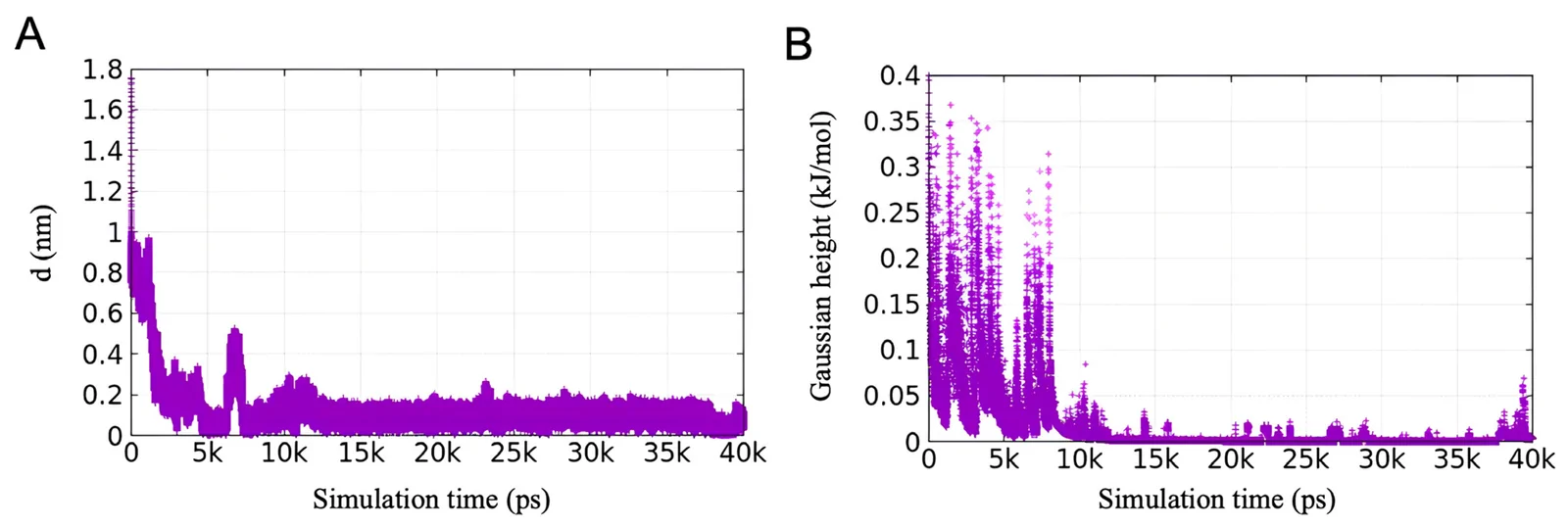
Time evolution of (**A**) d of CVs (COM distance between preQ1 and RNA binding pocket defined by A14·G11·C7 triplet and C16·G5 base pair above and below preQ1 binding site) and (**B**) Gaussian height in a 40 ns-long well-tempered metadynamics simulation of preQ1 riboswitch aptamer system.

**Figure 9:**
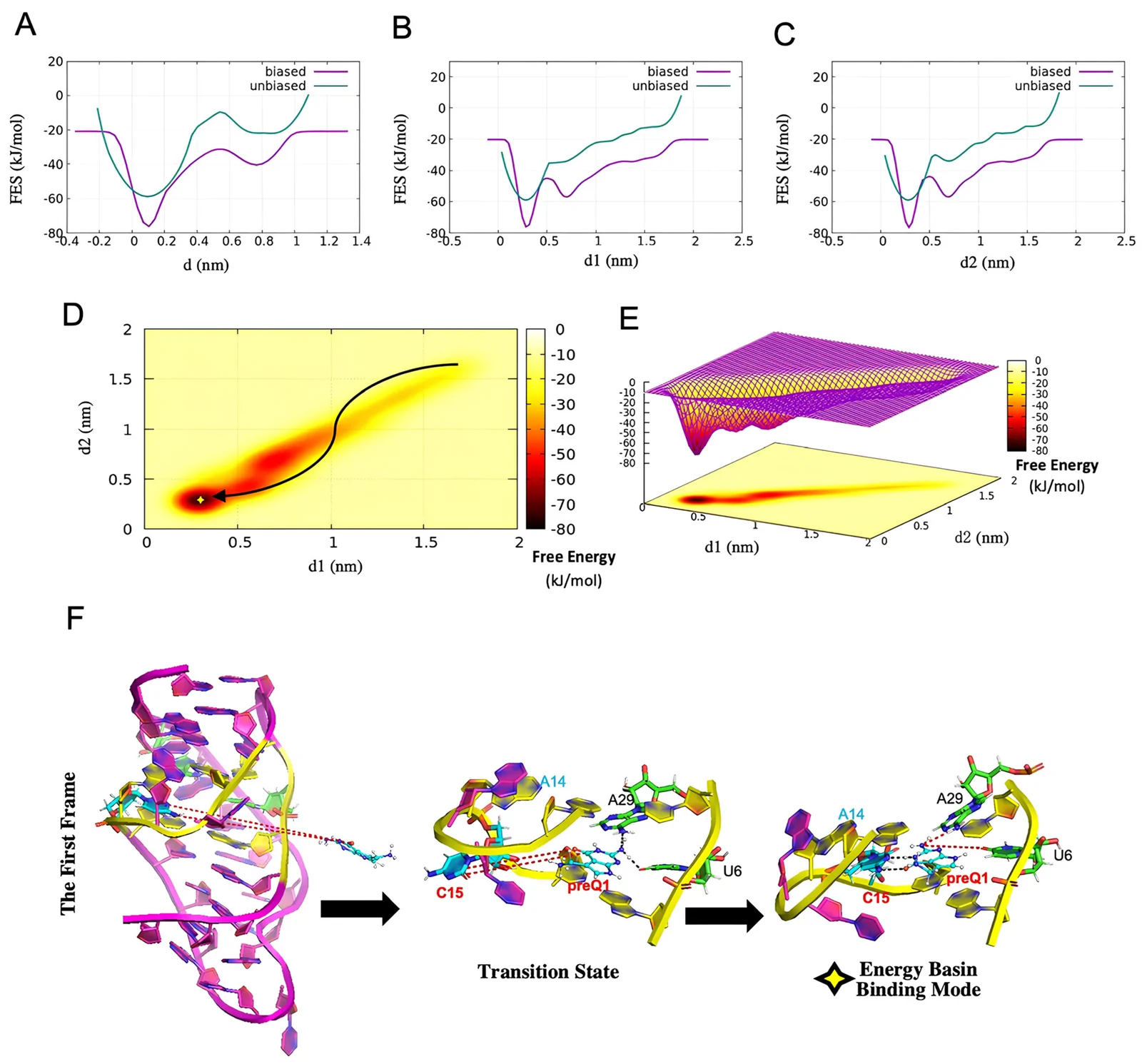
Comparison of the binding FES calculated from the metadynamics bias potential between biased (in purple) and unbiased (by reweighting, in green) as a function of (**A**) d of CVs (COM distance between preQ1 and binding pocket); (**B**) d1 of CVs (the distance between preQ1:O6 and C15:N4); (**C**) d2 of CVs (the distance between preQ1:N1 and C15:N3). Two-dimensional free energy landscape projected onto d1 and d2 (**D**) presented in 2D; (**E**) presented in 3D; (**F**) Binding pathway and mechanism. In the transition state, the amino group of preQ1 hydrogen bonded with carbonyl oxygen of the U6 base and the amino nitrogen of the A29 base, with C15 in the bulge state.

**Figure 11:**
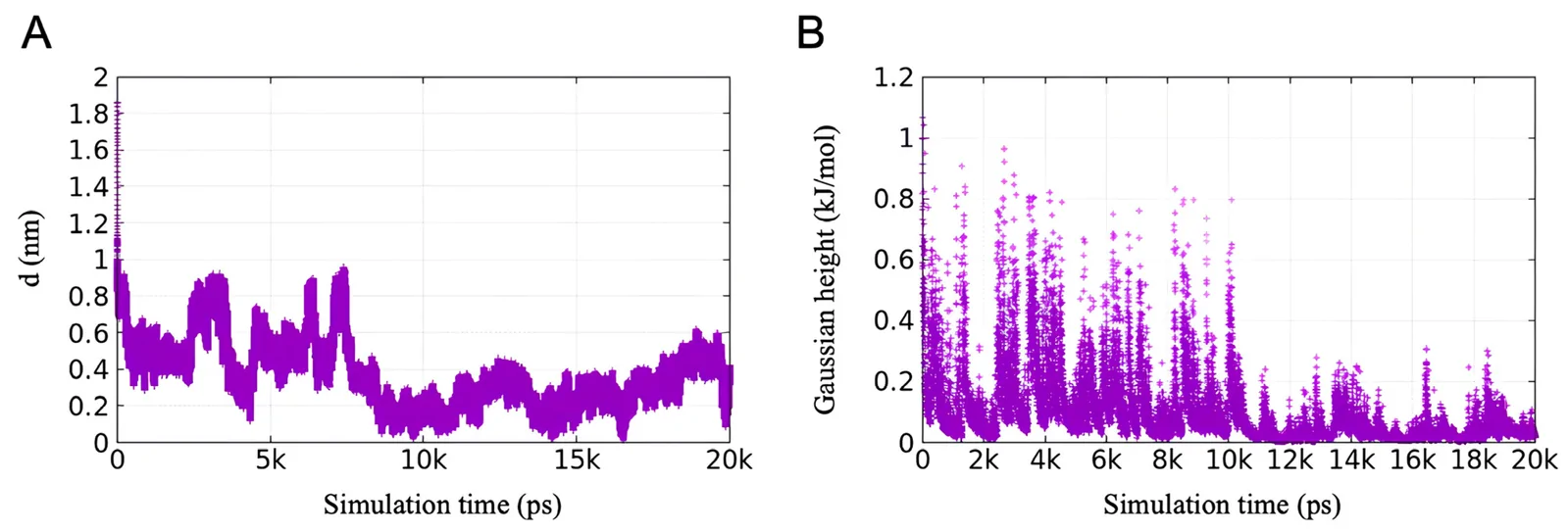
Time evolution of (**A**) d of CVs (COM distance between adenine and RNA binding pocket defined by C50·U75·A21 and A73·A52·U22 triplet above and below adenine binding site) and (**B**) Gaussian height in a 20 ns-long well-tempered metadynamics simulation of adenine riboswitch aptamer domain system.

**Figure 12:**
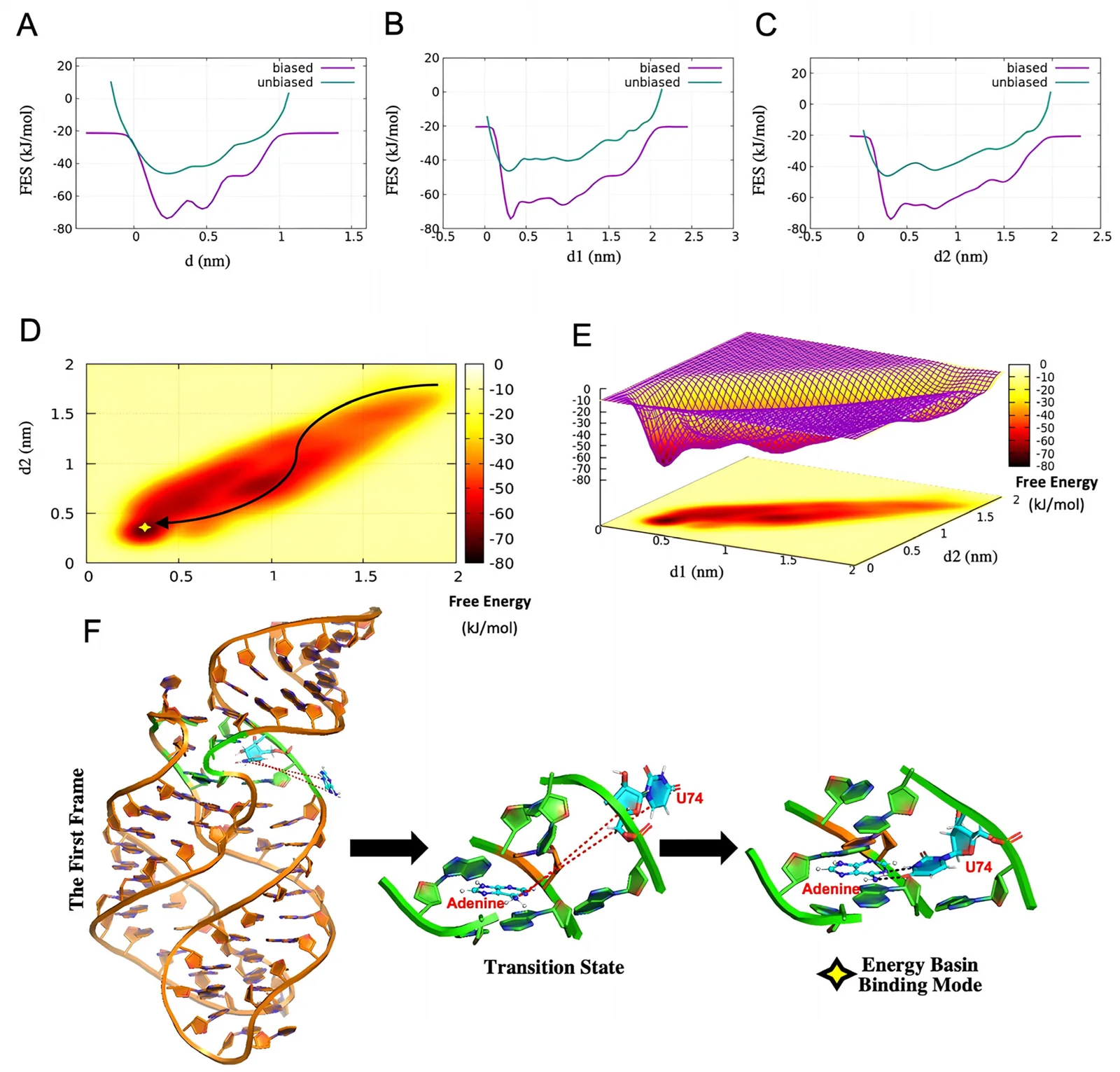
Comparison of the binding FES calculated from the metadynamics bias potential between biased (in purple) and unbiased (by reweighting, in green) as a function of (**A**) d of CVs (COM distance between adenine and binding pocket); (**B**) d1 of CVs (the distance between ADE:N6 and U74:O4); (**C**) d2 of CVs (the distance between ADE:N1 and U74:N3). Two-dimensional free energy landscape projected onto d1 and d2 (**D**) presented in 2D; (**E**) presented in 3D; (**F**) Binding pathway and mechanism. U74 swung back to the major groove from the bulge state in transition and paired with adenine, which stabilized the triplets above and below.

**Figure 14:**
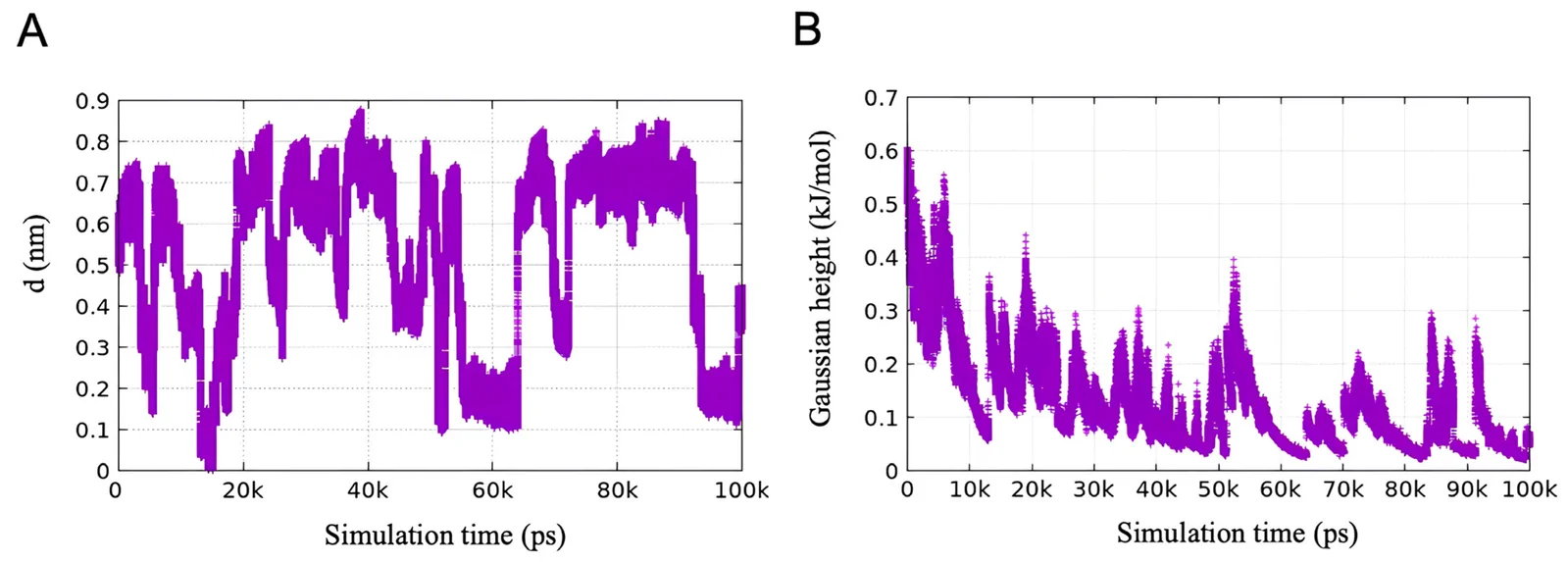
Time evolution of (**A**) d of CVs (COM distance between citrulline and RNA binding pocket defined by G3-G4-U5-A7-G9-G25-G28-U29) and (**B**) Gaussian height in a 100 ns-long well-tempered docking metadynamics simulation of the citrulline-RNA aptamer system.

**Figure 15:**
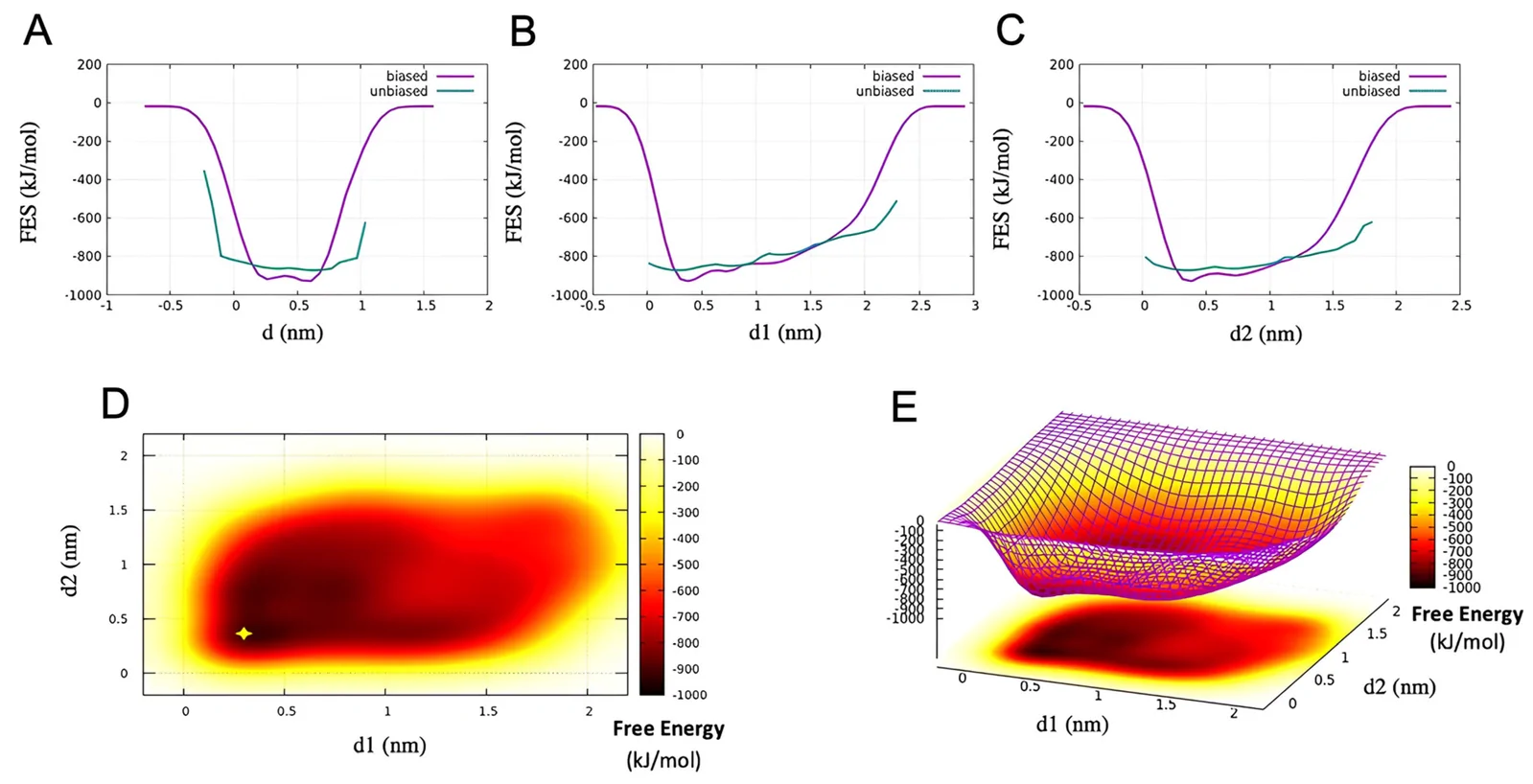
Comparison of the binding FES calculated from the metadynamics bias potential between biased (in purple) and unbiased (by reweighting, in green) as a function of (**A**) d of CVs (COM distance between citrulline and RNA binding pocket); (**B**) d1 of CVs (the distance between CIR:O7 and U29:N3); (**C**) d2 of CVs (the distance between CIR:N8 and G4:N7). Two-dimensional free energy landscape onto d1 and d2 (**D**) presented in 2D; (**E**) presented in 3D.

**Figure 17:**
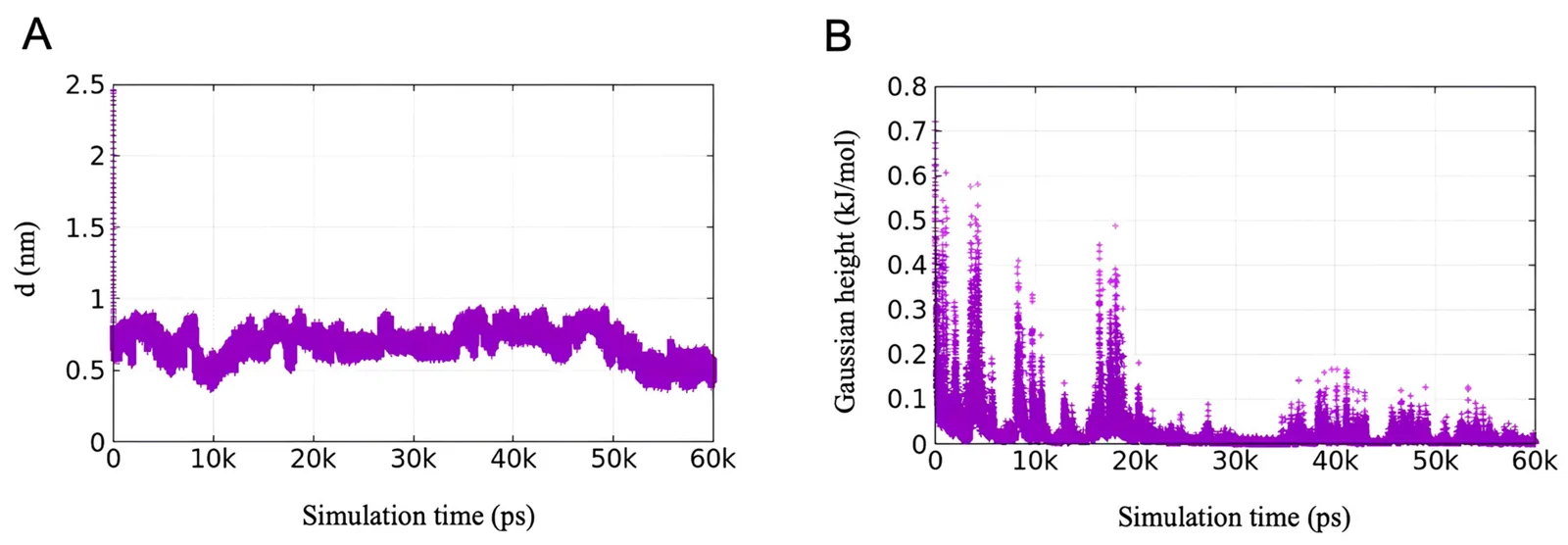
Time evolution of (**A**) d of CVs (COM distance between arginine and RNA binding pocket defined by A3-G4-G5-A7-G9-G25-G28-C29) and (**B**) Gaussian height in a 60 ns-long well-tempered docking metadynamics simulation of the arginine/RNA aptamer system.

**Table 1:** Parameters for metadynamics simulations.

System	Initial Gaussian Height *W*_0_ (kJ mol^−1^)	Bias Factor γ	Gaussian Width σ (nm)	Gaussian Deposition Stride τ (steps)	Simulation Time *t* (ns)	UPPER_ WALLS Restraints Force Constant *k* (kJ mol^−1^ nm^−2^)	UPPER_ WALLS Restrain Threshold *r* (nm)	Position Restraints on RNA Force Constant *k* (kJ mol^−1^ nm^−2^)
1FMN	0.3	4	0.27	500	30	5000	0.8	-
3Q50	0.3	4	0.27	500	40	5000	0.8	-
5SWE	0.8	4	0.27	500	20	5000	0.8	-
1KOD	0.6	100	0.20	500	100	5000	0.6	500
1KOC	0.6	6	0.23	500	60	5000	0.7	500

## Data Availability

The data presented in this study (all atomic structure, topology, parameter (for system and force field description), trajectory, and script files for reproducing and analyzing these five metadynamics simulations) are openly available in Zenodo at https://zenodo.org/records/17081250 accessed on 15 September 2025.

## Supplementary Materials

The following supporting information can be downloaded at: https://www.mdpi.com/article/10.3390/molecules31193428/s1, FMN_RNA aptamer complex (Movie S1); PreQ1 riboswitch aptamer complex (Movie S2); Adenine riboswitch aptamer complex (Movie S3); Citrulline_RNA aptamer complex (Movie S4); Arginine_RNA aptamer complex (Movie S5); Parameterization of five small molecule ligands (File S1).
