# Supplementary material for: Recovering Small Molecule Ligand—RNA Binding Pocket Binding Modes and Exploring Potential Binding Pathways Using Metadynamics
Source: Molecules. 2026 Sep 26;31(19):3428. doi: 10.3390/molecules31193428 (PMC13665804; doi:10.3390/molecules31193428)
Supplement: Supplementary file 1 [file molecules-31-03428-s001.zip › File S1_Parameterization of five small molecule ligands.pdf]

SUPPORTING INFORMATION

Predicting Small Molecule Ligand – RNA Binding Pocket Binding Modes Using Metadynamics

Zhixue Bai[1] and Alan Chen[1,*]

[1]Department of Chemistry and the RNA Institute, University at Albany, State University of New York, Albany, NY, 12222, USA

*Corresponding authors: achen6@albany.edu *(A.C.)*

PARAMETERIZATION OF FIVE SMALL MOLECULE LIGANDS

FMN Parameterization

```
[ atomtypes ]
;name   bond_type     mass     charge   ptype   sigma         epsilon       Amb
 nc       nc          0.00000  0.00000   A     3.08750e-01   5.69031e-01 ; 1.82  0.1700
 c        c           0.00000  0.00000   A     3.22968e-01   2.87859e-01 ; 1.91  0.0860
 o        o           0.00000  0.00000   A     2.95992e-01   8.78640e-01 ; 1.66  0.2100
 or       o           0.00000  0.00000   A     2.91192e-01   7.02912e-01 ; 1.66  0.2100
 n        n           0.00000  0.00000   A     3.08750e-01   5.69031e-01 ; 1.82  0.1700
 hn       hn          0.00000  0.00000   A     1.06908e-01   6.56888e-02 ; 0.60  0.0157
 cd       cd          0.00000  0.00000   A     3.22968e-01   2.87859e-01 ; 1.91  0.0860
 ca       ca          0.00000  0.00000   A     3.22968e-01   2.87859e-01 ; 1.91  0.0860
 ha       ha          0.00000  0.00000   A     2.59964e-01   6.27600e-02 ; 1.46  0.0150
 c3       c3          0.00000  0.00000   A     3.39967e-01   4.57730e-01 ; 1.91  0.1094
 hc       hc          0.00000  0.00000   A     2.64953e-01   6.56888e-02 ; 1.49  0.0157
 na       na          0.00000  0.00000   A     3.08750e-01   5.69031e-01 ; 1.82  0.1700
 h1       h1          0.00000  0.00000   A     2.47135e-01   6.56888e-02 ; 1.39  0.0157
 oh       oh          0.00000  0.00000   A     3.06647e-01   8.80314e-01 ; 1.72  0.2104
 ho       ho          0.00000  0.00000   A     0.00000e+00   0.00000e+00 ; 0.00  0.0000
 os       os          0.00000  0.00000   A     3.00001e-01   7.11280e-01 ; 1.68  0.1700
 p5       p5          0.00000  0.00000   A     3.74177e-01   8.36800e-01 ; 2.10  0.2000

[ moleculetype ]
;name            nrexcl
 FMN              3

[ atoms ]
;   nr  type  resi  res  atom  cgnr     charge      mass       ; qtot   bond_type
     1   nc     1   FMN    N1    1    -0.629401     14.01000 ; qtot -0.629
     2    c     1   FMN    C2    2     0.728601     12.01000 ; qtot 0.099
     3   or     1   FMN    O2    3    -0.628901     16.00000 ; qtot -0.530
     4    n     1   FMN    N3    4    -0.446500     14.01000 ; qtot -0.976
     5   hn     1   FMN   HN3    5     0.302200      1.00800 ; qtot -0.674
     6    c     1   FMN    C4    6     0.455900     12.01000 ; qtot -0.218
     7   or     1   FMN    O4    7    -0.566301     16.00000 ; qtot -0.784
     8   cd     1   FMN   C4A    8     0.339500     12.01000 ; qtot -0.445
     9   nc     1   FMN    N5    9    -0.497400     14.01000 ; qtot -0.942
    10   ca     1   FMN   C5A   10     0.266000     12.01000 ; qtot -0.676
    11   ca     1   FMN    C6   11    -0.238800     12.01000 ; qtot -0.915
    12   ha     1   FMN    H6   12     0.148000      1.00800 ; qtot -0.767
    13   ca     1   FMN    C7   13     0.046000     12.01000 ; qtot -0.721
    14   c3     1   FMN   C7M   14    -0.203000     12.01000 ; qtot -0.924
    15   hc     1   FMN  HM71   15     0.055100      1.00800 ; qtot -0.869
    16   hc     1   FMN  HM72   16     0.055100      1.00800 ; qtot -0.814
    17   hc     1   FMN  HM73   17     0.055100      1.00800 ; qtot -0.759
    18   ca     1   FMN    C8   18     0.082400     12.01000 ; qtot -0.676
    19   c3     1   FMN   C8M   19    -0.022900     12.01000 ; qtot -0.699
    20   hc     1   FMN  HM81   20     0.039100      1.00800 ; qtot -0.660
    21   hc     1   FMN  HM82   21     0.039100      1.00800 ; qtot -0.621
    22   hc     1   FMN  HM83   22     0.039100      1.00800 ; qtot -0.582
    23   ca     1   FMN    C9   23    -0.247200     12.01000 ; qtot -0.829
    24   ha     1   FMN    H9   24     0.219200      1.00800 ; qtot -0.610
    25   ca     1   FMN   C9A   25    -0.011000     12.01000 ; qtot -0.621
    26   na     1   FMN   N10   26     0.020800     14.01000 ; qtot -0.600
    27   cd     1   FMN   C10   27     0.304800     12.01000 ; qtot -0.295
    28   c3     1   FMN   C1'   28    -0.066300     12.01000 ; qtot -0.362
    29   h1     1   FMN  H1'1   29     0.073800      1.00800 ; qtot -0.288
    30   h1     1   FMN  H1'2   30     0.073800      1.00800 ; qtot -0.214
    31   c3     1   FMN   C2'   31     0.148400     12.01000 ; qtot -0.066
    32   h1     1   FMN   H2'   32     0.078700      1.00800 ; qtot 0.013
    33   oh     1   FMN   O2'   33    -0.605401     16.00000 ; qtot -0.592
    34   ho     1   FMN  HO2'   34     0.412500      1.00800 ; qtot -0.180
    35   c3     1   FMN   C3'   35     0.123400     12.01000 ; qtot -0.057
    36   h1     1   FMN   H3'   36     0.055300      1.00800 ; qtot -0.001
    37   oh     1   FMN   O3'   37    -0.685901     16.00000 ; qtot -0.687
    38   ho     1   FMN  HO3'   38     0.456100      1.00800 ; qtot -0.231
    39   c3     1   FMN   C4'   39     0.072100     12.01000 ; qtot -0.159
    40   h1     1   FMN   H4'   40     0.103400      1.00800 ; qtot -0.056
```

```
    41   oh      1   FMN   O4'   41    -0.706901     16.00000 ; qtot -0.762
    42   ho      1   FMN  HO4'   42     0.466000      1.00800 ; qtot -0.296
    43   c3      1   FMN   C5'   43     0.075900     12.01000 ; qtot -0.221
    44   h1      1   FMN  H5'1   44     0.041600      1.00800 ; qtot -0.179
    45   h1      1   FMN  H5'2   45     0.041600      1.00800 ; qtot -0.137
    46   os      1   FMN   O5'   46    -0.476000     16.00000 ; qtot -0.613
    47   p5      1   FMN     P   47     1.260803     30.97000 ; qtot 0.648
    48    o      1   FMN   O1P   48    -0.882501     16.00000 ; qtot -0.235
    49    o      1   FMN   O2P   49    -0.882501     16.00000 ; qtot -1.117
    50    o      1   FMN   O3P   50    -0.882501     16.00000 ; qtot -2.000

[ bonds ]
;   ai     aj funct   r             k
     1      2   1    1.3784e-01    3.5840e+05 ;     N1 - C2
     1     27   1    1.3350e-01    4.1388e+05 ;     N1 - C10
     2      3   1    1.2140e-01    5.4225e+05 ;     C2 - O2
     2      4   1    1.3450e-01    4.0016e+05 ;     C2 - N3
     4      5   1    1.0090e-01    3.4326e+05 ;     N3 - HN3
     4      6   1    1.3450e-01    4.0016e+05 ;     N3 - C4
     6      7   1    1.2140e-01    5.4225e+05 ;     C4 - O4
     6      8   1    1.4620e-01    3.1581e+05 ;     C4 - C4A
     8      9   1    1.3350e-01    4.1388e+05 ;    C4A - N5
     8     27   1    1.4290e-01    3.5003e+05 ;    C4A - C10
     9     10   1    1.3360e-01    4.1246e+05 ;     N5 - C5A
    10     11   1    1.3870e-01    4.0033e+05 ;    C5A - C6
    10     25   1    1.3870e-01    4.0033e+05 ;    C5A - C9A
    11     12   1    1.0870e-01    2.8811e+05 ;     C6 - H6
    11     13   1    1.3870e-01    4.0033e+05 ;     C6 - C7
    13     14   1    1.5130e-01    2.7070e+05 ;     C7 - C7M
    13     18   1    1.3870e-01    4.0033e+05 ;     C7 - C8
    14     15   1    1.0920e-01    2.8225e+05 ;    C7M - HM71
    14     16   1    1.0920e-01    2.8225e+05 ;    C7M - HM72
    14     17   1    1.0920e-01    2.8225e+05 ;    C7M - HM73
    18     19   1    1.5130e-01    2.7070e+05 ;     C8 - C8M
    18     23   1    1.3870e-01    4.0033e+05 ;     C8 - C9
    19     20   1    1.0920e-01    2.8225e+05 ;    C8M - HM81
    19     21   1    1.0920e-01    2.8225e+05 ;    C8M - HM82
    19     22   1    1.0920e-01    2.8225e+05 ;    C8M - HM83
    23     24   1    1.0870e-01    2.8811e+05 ;     C9 - H9
    23     25   1    1.3870e-01    4.0033e+05 ;     C9 - C9A
    25     26   1    1.3500e-01    3.9355e+05 ;    C9A - N10
    26     27   1    1.3710e-01    3.6719e+05 ;    N10 - C10
    26     28   1    1.4560e-01    2.8008e+05 ;    N10 - C1'
    28     29   1    1.0930e-01    2.8108e+05 ;    C1' - H1'1
    28     30   1    1.0930e-01    2.8108e+05 ;    C1' - H1'2
    28     31   1    1.5350e-01    2.5363e+05 ;    C1' - C2'
    31     32   1    1.0930e-01    2.8108e+05 ;    C2' - H2'
    31     33   1    1.4260e-01    2.6284e+05 ;    C2' - O2'
    31     35   1    1.5350e-01    2.5363e+05 ;    C2' - C3'
    33     34   1    9.7400e-02    3.0928e+05 ;    O2' - HO2'
    35     36   1    1.0930e-01    2.8108e+05 ;    C3' - H3'
    35     37   1    1.4260e-01    2.6284e+05 ;    C3' - O3'
    35     39   1    1.5350e-01    2.5363e+05 ;    C3' - C4'
    37     38   1    9.7400e-02    3.0928e+05 ;    O3' - HO3'
    39     40   1    1.0930e-01    2.8108e+05 ;    C4' - H4'
    39     41   1    1.4260e-01    2.6284e+05 ;    C4' - O4'
    39     43   1    1.5350e-01    2.5363e+05 ;    C4' - C5'
    41     42   1    9.7400e-02    3.0928e+05 ;    O4' - HO4'
    43     44   1    1.0930e-01    2.8108e+05 ;    C5' - H5'1
    43     45   1    1.0930e-01    2.8108e+05 ;    C5' - H5'2
    43     46   1    1.4390e-01    2.5230e+05 ;    C5' - O5'
    46     47   1    1.6020e-01    2.8660e+05 ;    O5' - P
    47     48   1    1.4810e-01    4.0811e+05 ;      P - O1P
    47     49   1    1.4810e-01    4.0811e+05 ;      P - O2P
    47     50   1    1.4810e-01    4.0811e+05 ;      P - O3P

[ pairs ]
;   ai     aj    funct
     1      5      1 ;     N1 - HN3
     1      6      1 ;     N1 - C4
     1      9      1 ;     N1 - N5
     1     25      1 ;     N1 - C9A
```

1	28	1 ;	N1 - C1'
2	7	1 ;	C2 - O4
2	8	1 ;	C2 - C4A
2	26	1 ;	C2 - N10
3	5	1 ;	O2 - HN3
3	6	1 ;	O2 - C4
4	9	1 ;	N3 - N5
5	7	1 ;	HN3 - O4
5	8	1 ;	HN3 - C4A
6	10	1 ;	C4 - C5A
6	26	1 ;	C4 - N10
7	9	1 ;	O4 - N5
7	27	1 ;	O4 - C10
8	11	1 ;	C4A - C6
8	25	1 ;	C4A - C9A
8	28	1 ;	C4A - C1'
9	12	1 ;	N5 - H6
9	13	1 ;	N5 - C7
9	23	1 ;	N5 - C9
9	26	1 ;	N5 - N10
10	14	1 ;	C5A - C7M
10	18	1 ;	C5A - C8
10	24	1 ;	C5A - H9
10	27	1 ;	C5A - C10
10	28	1 ;	C5A - C1'
11	15	1 ;	C6 - HM71
11	16	1 ;	C6 - HM72
11	17	1 ;	C6 - HM73
11	19	1 ;	C6 - C8M
11	23	1 ;	C6 - C9
11	26	1 ;	C6 - N10
12	14	1 ;	H6 - C7M
12	18	1 ;	H6 - C8
12	25	1 ;	H6 - C9A
13	20	1 ;	C7 - HM81
13	21	1 ;	C7 - HM82
13	22	1 ;	C7 - HM83
13	24	1 ;	C7 - H9
13	25	1 ;	C7 - C9A
14	19	1 ;	C7M - C8M
14	23	1 ;	C7M - C9
15	18	1 ;	HM71 - C8
16	18	1 ;	HM72 - C8
17	18	1 ;	HM73 - C8
18	26	1 ;	C8 - N10
19	24	1 ;	C8M - H9
19	25	1 ;	C8M - C9A
20	23	1 ;	HM81 - C9
21	23	1 ;	HM82 - C9
22	23	1 ;	HM83 - C9
23	27	1 ;	C9 - C10
23	28	1 ;	C9 - C1'
24	26	1 ;	H9 - N10
25	29	1 ;	C9A - H1'1
25	30	1 ;	C9A - H1'2
25	31	1 ;	C9A - C2'
26	32	1 ;	N10 - H2'
26	33	1 ;	N10 - O2'
26	35	1 ;	N10 - C3'
27	3	1 ;	C10 - O2
27	4	1 ;	C10 - N3
27	29	1 ;	C10 - H1'1
27	30	1 ;	C10 - H1'2
27	31	1 ;	C10 - C2'
28	34	1 ;	C1' - HO2'
28	36	1 ;	C1' - H3'
28	37	1 ;	C1' - O3'
28	39	1 ;	C1' - C4'
29	32	1 ;	H1'1 - H2'
29	33	1 ;	H1'1 - O2'
29	35	1 ;	H1'1 - C3'

```
     30      32       1 ;   H1'2 - H2'
     30      33       1 ;   H1'2 - O2'
     30      35       1 ;   H1'2 - C3'
     31      38       1 ;    C2' - HO3'
     31      40       1 ;    C2' - H4'
     31      41       1 ;    C2' - O4'
     31      43       1 ;    C2' - C5'
     32      34       1 ;    H2' - HO2'
     32      36       1 ;    H2' - H3'
     32      37       1 ;    H2' - O3'
     32      39       1 ;    H2' - C4'
     33      36       1 ;    O2' - H3'
     33      37       1 ;    O2' - O3'
     33      39       1 ;    O2' - C4'
     34      35       1 ;   HO2' - C3'
     35      42       1 ;    C3' - HO4'
     35      44       1 ;    C3' - H5'1
     35      45       1 ;    C3' - H5'2
     35      46       1 ;    C3' - O5'
     36      38       1 ;    H3' - HO3'
     36      40       1 ;    H3' - H4'
     36      41       1 ;    H3' - O4'
     36      43       1 ;    H3' - C5'
     37      40       1 ;    O3' - H4'
     37      41       1 ;    O3' - O4'
     37      43       1 ;    O3' - C5'
     38      39       1 ;   HO3' - C4'
     39      47       1 ;    C4' - P
     40      42       1 ;    H4' - HO4'
     40      44       1 ;    H4' - H5'1
     40      45       1 ;    H4' - H5'2
     40      46       1 ;    H4' - O5'
     41      44       1 ;    O4' - H5'1
     41      45       1 ;    O4' - H5'2
     41      46       1 ;    O4' - O5'
     42      43       1 ;   HO4' - C5'
     43      48       1 ;    C5' - O1P
     43      49       1 ;    C5' - O2P
     43      50       1 ;    C5' - O3P
     44      47       1 ;   H5'1 - P
     45      47       1 ;   H5'2 - P

[ angles ]
;   ai     aj     ak    funct   theta         cth
     1      2      3      1    1.2398e+02    6.1965e+02 ;     N1 - C2     - O2
     1      2      4      1    1.1705e+02    6.0827e+02 ;     N1 - C2     - N3
     1     27      8      1    1.1256e+02    5.9538e+02 ;     N1 - C10    - C4A
     1     27     26      1    1.1202e+02    6.2576e+02 ;     N1 - C10    - N10
     2      1     27      1    1.2032e+02    5.5689e+02 ;     C2 - N1     - C10
     2      4      5      1    1.1846e+02    4.1179e+02 ;     C2 - N3     - HN3
     2      4      6      1    1.2714e+02    5.4668e+02 ;     C2 - N3     - C4
     3      2      4      1    1.2203e+02    6.3455e+02 ;     O2 - C2     - N3
     4      6      7      1    1.2203e+02    6.3455e+02 ;     N3 - C4     - O4
     4      6      8      1    1.1186e+02    5.8735e+02 ;     N3 - C4     - C4A
     5      4      6      1    1.1846e+02    4.1179e+02 ;    HN3 - N3     - C4
     6      8      9      1    1.2186e+02    5.6442e+02 ;     C4 - C4A    - N5
     6      8     27      1    1.2269e+02    5.3321e+02 ;     C4 - C4A    - C10
     7      6      8      1    1.2571e+02    5.7664e+02 ;     O4 - C4     - C4A
     8      9     10      1    1.0424e+02    6.0810e+02 ;    C4A - N5     - C5A
     8     27     26      1    1.0680e+02    6.0425e+02 ;    C4A - C10    - N10
     9      8     27      1    1.1256e+02    5.9538e+02 ;     N5 - C4A    - C10
     9     10     11      1    1.1972e+02    5.8693e+02 ;     N5 - C5A    - C6
     9     10     25      1    1.1972e+02    5.8693e+02 ;     N5 - C5A    - C9A
    10     11     12      1    1.2001e+02    4.0551e+02 ;    C5A - C6     - H6
    10     11     13      1    1.1997e+02    5.6216e+02 ;    C5A - C6     - C7
    10     25     23      1    1.1997e+02    5.6216e+02 ;    C5A - C9A    - C9
    10     25     26      1    1.1834e+02    5.8752e+02 ;    C5A - C9A    - N10
    11     10     25      1    1.1997e+02    5.6216e+02 ;     C6 - C5A    - C9A
    11     13     14      1    1.2063e+02    5.3421e+02 ;     C6 - C7     - C7M
    11     13     18      1    1.1997e+02    5.6216e+02 ;     C6 - C7     - C8
    12     11     13      1    1.2001e+02    4.0551e+02 ;     H6 - C6     - C7
```

```
    13     14     15      1    1.1015e+02    3.9296e+02 ;      C7 - C7M    - HM71
    13     14     16      1    1.1015e+02    3.9296e+02 ;      C7 - C7M    - HM72
    13     14     17      1    1.1015e+02    3.9296e+02 ;      C7 - C7M    - HM73
    13     18     19      1    1.2063e+02    5.3421e+02 ;      C7 - C8     - C8M
    13     18     23      1    1.1997e+02    5.6216e+02 ;      C7 - C8     - C9
    14     13     18      1    1.2063e+02    5.3421e+02 ;     C7M - C7     - C8
    15     14     16      1    1.0835e+02    3.2995e+02 ;    HM71 - C7M    - HM72
    15     14     17      1    1.0835e+02    3.2995e+02 ;    HM71 - C7M    - HM73
    16     14     17      1    1.0835e+02    3.2995e+02 ;    HM72 - C7M    - HM73
    18     19     20      1    1.1015e+02    3.9296e+02 ;      C8 - C8M    - HM81
    18     19     21      1    1.1015e+02    3.9296e+02 ;      C8 - C8M    - HM82
    18     19     22      1    1.1015e+02    3.9296e+02 ;      C8 - C8M    - HM83
    18     23     24      1    1.2001e+02    4.0551e+02 ;      C8 - C9     - H9
    18     23     25      1    1.1997e+02    5.6216e+02 ;      C8 - C9     - C9A
    19     18     23      1    1.2063e+02    5.3421e+02 ;     C8M - C8     - C9
    20     19     21      1    1.0835e+02    3.2995e+02 ;    HM81 - C8M    - HM82
    20     19     22      1    1.0835e+02    3.2995e+02 ;    HM81 - C8M    - HM83
    21     19     22      1    1.0835e+02    3.2995e+02 ;    HM82 - C8M    - HM83
    23     25     26      1    1.1834e+02    5.8752e+02 ;      C9 - C9A    - N10
    24     23     25      1    1.2001e+02    4.0551e+02 ;      H9 - C9     - C9A
    25     26     27      1    1.1315e+02    5.7287e+02 ;     C9A - N10    - C10
    25     26     28      1    1.2436e+02    5.2844e+02 ;     C9A - N10    - C1'
    26     28     29      1    1.0945e+02    4.1756e+02 ;     N10 - C1'    - H1'1
    26     28     30      1    1.0945e+02    4.1756e+02 ;     N10 - C1'    - H1'2
    26     28     31      1    1.1281e+02    5.5003e+02 ;     N10 - C1'    - C2'
    27     26     28      1    1.2509e+02    5.2350e+02 ;     C10 - N10    - C1'
    28     31     32      1    1.1007e+02    3.8794e+02 ;     C1' - C2'    - H2'
    28     31     33      1    1.0943e+02    5.6668e+02 ;     C1' - C2'    - O2'
    28     31     35      1    1.1063e+02    5.2894e+02 ;     C1' - C2'    - C3'
    29     28     30      1    1.0955e+02    3.2786e+02 ;    H1'1 - C1'    - H1'2
    29     28     31      1    1.1007e+02    3.8794e+02 ;    H1'1 - C1'    - C2'
    30     28     31      1    1.1007e+02    3.8794e+02 ;    H1'2 - C1'    - C2'
    31     33     34      1    1.0816e+02    3.9405e+02 ;     C2' - O2'    - HO2'
    31     35     36      1    1.1007e+02    3.8794e+02 ;     C2' - C3'    - H3'
    31     35     37      1    1.0943e+02    5.6668e+02 ;     C2' - C3'    - O3'
    31     35     39      1    1.1063e+02    5.2894e+02 ;     C2' - C3'    - C4'
    32     31     33      1    1.0988e+02    4.2652e+02 ;     H2' - C2'    - O2'
    32     31     35      1    1.1007e+02    3.8794e+02 ;     H2' - C2'    - C3'
    33     31     35      1    1.0943e+02    5.6668e+02 ;     O2' - C2'    - C3'
    35     37     38      1    1.0816e+02    3.9405e+02 ;     C3' - O3'    - HO3'
    35     39     40      1    1.1007e+02    3.8794e+02 ;     C3' - C4'    - H4'
    35     39     41      1    1.0943e+02    5.6668e+02 ;     C3' - C4'    - O4'
    35     39     43      1    1.1063e+02    5.2894e+02 ;     C3' - C4'    - C5'
    36     35     37      1    1.0988e+02    4.2652e+02 ;     H3' - C3'    - O3'
    36     35     39      1    1.1007e+02    3.8794e+02 ;     H3' - C3'    - C4'
    37     35     39      1    1.0943e+02    5.6668e+02 ;     O3' - C3'    - C4'
    39     41     42      1    1.0816e+02    3.9405e+02 ;     C4' - O4'    - HO4'
    39     43     44      1    1.1007e+02    3.8794e+02 ;     C4' - C5'    - H5'1
    39     43     45      1    1.1007e+02    3.8794e+02 ;     C4' - C5'    - H5'2
    39     43     46      1    1.0842e+02    5.6718e+02 ;     C4' - C5'    - O5'
    40     39     41      1    1.0988e+02    4.2652e+02 ;     H4' - C4'    - O4'
    40     39     43      1    1.1007e+02    3.8794e+02 ;     H4' - C4'    - C5'
    41     39     43      1    1.0943e+02    5.6668e+02 ;     O4' - C4'    - C5'
    43     46     47      1    1.1800e+02    5.1882e+02 ;     C5' - O5'    - P
    44     43     45      1    1.0955e+02    3.2786e+02 ;    H5'1 - C5'    - H5'2
    44     43     46      1    1.0882e+02    4.2543e+02 ;    H5'1 - C5'    - O5'
    45     43     46      1    1.0882e+02    4.2543e+02 ;    H5'2 - C5'    - O5'
    46     47     48      1    1.1609e+02    5.8861e+02 ;     O5' - P      - O1P
    46     47     49      1    1.1609e+02    5.8861e+02 ;     O5' - P      - O2P
    46     47     50      1    1.1609e+02    5.8861e+02 ;     O5' - P      - O3P
    48     47     49      1    1.1580e+02    6.1530e+02 ;     O1P - P      - O2P
    48     47     50      1    1.1580e+02    6.1530e+02 ;     O1P - P      - O3P
    49     47     50      1    1.1580e+02    6.1530e+02 ;     O2P - P      - O3P

[ dihedrals ] ; propers
; treated as RBs in GROMACS to use combine multiple AMBER torsions per quartet
;    i      j      k      l   func     C0          C1          C2          C3          C4          C5
     1      2      4      5      3   20.92000    0.00000  -20.92000    0.00000    0.00000    0.00000 ;     N1-    C2-    N3-   HN3
     1      2      4      6      3   20.92000    0.00000  -20.92000    0.00000    0.00000    0.00000 ;     N1-    C2-    N3-    C4
     1     27      8      6      3   33.47200    0.00000  -33.47200    0.00000    0.00000    0.00000 ;     N1-   C10-   C4A-    C4
     1     27      8      9      3   33.47200    0.00000  -33.47200    0.00000    0.00000    0.00000 ;     N1-   C10-   C4A-    N5
     1     27     26     25      3   14.22560    0.00000  -14.22560    0.00000    0.00000    0.00000 ;     N1-   C10-   N10-   C9A
     1     27     26     28      3   14.22560    0.00000  -14.22560    0.00000    0.00000    0.00000 ;     N1-   C10-   N10-   C1'
     2      1     27      8      3   39.74800    0.00000  -39.74800    0.00000    0.00000    0.00000 ;     C2-    N1-   C10-   C4A
```

2	1	27	26	3	39.74800	0.00000	-39.74800	0.00000	0.00000	0.00000	;	C2-	N1-	C10-	N10
2	4	6	7	3	20.92000	0.00000	-20.92000	0.00000	0.00000	0.00000	;	C2-	N3-	C4-	O4
2	4	6	8	3	20.92000	0.00000	-20.92000	0.00000	0.00000	0.00000	;	C2-	N3-	C4-	C4A
3	2	4	5	3	29.28800	-8.36800	-20.92000	0.00000	0.00000	0.00000	;	O2-	C2-	N3-	HN3
3	2	4	6	3	20.92000	0.00000	-20.92000	0.00000	0.00000	0.00000	;	O2-	C2-	N3-	C4
4	6	8	9	3	24.05800	0.00000	-24.05800	0.00000	0.00000	0.00000	;	N3-	C4-	C4A-	N5
4	6	8	27	3	24.05800	0.00000	-24.05800	0.00000	0.00000	0.00000	;	N3-	C4-	C4A-	C10
5	4	6	7	3	29.28800	-8.36800	-20.92000	0.00000	0.00000	0.00000	;	HN3-	N3-	C4-	O4
5	4	6	8	3	20.92000	0.00000	-20.92000	0.00000	0.00000	0.00000	;	HN3-	N3-	C4-	C4A
6	8	9	10	3	39.74800	0.00000	-39.74800	0.00000	0.00000	0.00000	;	C4-	C4A-	N5-	C5A
6	8	27	26	3	33.47200	0.00000	-33.47200	0.00000	0.00000	0.00000	;	C4-	C4A-	C10-	N10
7	6	8	9	3	24.05800	0.00000	-24.05800	0.00000	0.00000	0.00000	;	O4-	C4-	C4A-	N5
7	6	8	27	3	24.05800	0.00000	-24.05800	0.00000	0.00000	0.00000	;	O4-	C4-	C4A-	C10
8	9	10	11	3	40.16640	0.00000	-40.16640	0.00000	0.00000	0.00000	;	C4A-	N5-	C5A-	C6
8	9	10	25	3	40.16640	0.00000	-40.16640	0.00000	0.00000	0.00000	;	C4A-	N5-	C5A-	C9A
8	27	26	25	3	14.22560	0.00000	-14.22560	0.00000	0.00000	0.00000	;	C4A-	C10-	N10-	C9A
8	27	26	28	3	14.22560	0.00000	-14.22560	0.00000	0.00000	0.00000	;	C4A-	C10-	N10-	C1'
9	8	27	26	3	33.47200	0.00000	-33.47200	0.00000	0.00000	0.00000	;	N5-	C4A-	C10-	N10
9	10	11	12	3	30.33400	0.00000	-30.33400	0.00000	0.00000	0.00000	;	N5-	C5A-	C6-	H6
9	10	11	13	3	30.33400	0.00000	-30.33400	0.00000	0.00000	0.00000	;	N5-	C5A-	C6-	C7
9	10	25	23	3	30.33400	0.00000	-30.33400	0.00000	0.00000	0.00000	;	N5-	C5A-	C9A-	C9
9	10	25	26	3	30.33400	0.00000	-30.33400	0.00000	0.00000	0.00000	;	N5-	C5A-	C9A-	N10
10	9	8	27	3	39.74800	0.00000	-39.74800	0.00000	0.00000	0.00000	;	C5A-	N5-	C4A-	C10
10	11	13	14	3	30.33400	0.00000	-30.33400	0.00000	0.00000	0.00000	;	C5A-	C6-	C7-	C7M
10	11	13	18	3	30.33400	0.00000	-30.33400	0.00000	0.00000	0.00000	;	C5A-	C6-	C7-	C8
10	25	23	18	3	30.33400	0.00000	-30.33400	0.00000	0.00000	0.00000	;	C5A-	C9A-	C9-	C8
10	25	23	24	3	30.33400	0.00000	-30.33400	0.00000	0.00000	0.00000	;	C5A-	C9A-	C9-	H9
10	25	26	27	3	2.51040	0.00000	-2.51040	0.00000	0.00000	0.00000	;	C5A-	C9A-	N10-	C10
10	25	26	28	3	2.51040	0.00000	-2.51040	0.00000	0.00000	0.00000	;	C5A-	C9A-	N10-	C1'
11	10	25	23	3	30.33400	0.00000	-30.33400	0.00000	0.00000	0.00000	;	C6-	C5A-	C9A-	C9
11	10	25	26	3	30.33400	0.00000	-30.33400	0.00000	0.00000	0.00000	;	C6-	C5A-	C9A-	N10
11	13	14	15	3	0.00000	0.00000	0.00000	0.00000	0.00000	0.00000	;	C6-	C7-	C7M-	HM71
11	13	14	16	3	0.00000	0.00000	0.00000	0.00000	0.00000	0.00000	;	C6-	C7-	C7M-	HM72
11	13	14	17	3	0.00000	0.00000	0.00000	0.00000	0.00000	0.00000	;	C6-	C7-	C7M-	HM73
11	13	18	19	3	30.33400	0.00000	-30.33400	0.00000	0.00000	0.00000	;	C6-	C7-	C8-	C8M
11	13	18	23	3	30.33400	0.00000	-30.33400	0.00000	0.00000	0.00000	;	C6-	C7-	C8-	C9
12	11	10	25	3	30.33400	0.00000	-30.33400	0.00000	0.00000	0.00000	;	H6-	C6-	C5A-	C9A
12	11	13	14	3	30.33400	0.00000	-30.33400	0.00000	0.00000	0.00000	;	H6-	C6-	C7-	C7M
12	11	13	18	3	30.33400	0.00000	-30.33400	0.00000	0.00000	0.00000	;	H6-	C6-	C7-	C8
13	11	10	25	3	30.33400	0.00000	-30.33400	0.00000	0.00000	0.00000	;	C7-	C6-	C5A-	C9A
13	18	19	20	3	0.00000	0.00000	0.00000	0.00000	0.00000	0.00000	;	C7-	C8-	C8M-	HM81
13	18	19	21	3	0.00000	0.00000	0.00000	0.00000	0.00000	0.00000	;	C7-	C8-	C8M-	HM82
13	18	19	22	3	0.00000	0.00000	0.00000	0.00000	0.00000	0.00000	;	C7-	C8-	C8M-	HM83
13	18	23	24	3	30.33400	0.00000	-30.33400	0.00000	0.00000	0.00000	;	C7-	C8-	C9-	H9
13	18	23	25	3	30.33400	0.00000	-30.33400	0.00000	0.00000	0.00000	;	C7-	C8-	C9-	C9A
14	13	18	19	3	30.33400	0.00000	-30.33400	0.00000	0.00000	0.00000	;	C7M-	C7-	C8-	C8M
14	13	18	23	3	30.33400	0.00000	-30.33400	0.00000	0.00000	0.00000	;	C7M-	C7-	C8-	C9
15	14	13	18	3	0.00000	0.00000	0.00000	0.00000	0.00000	0.00000	;	HM71-	C7M-	C7-	C8
16	14	13	18	3	0.00000	0.00000	0.00000	0.00000	0.00000	0.00000	;	HM72-	C7M-	C7-	C8
17	14	13	18	3	0.00000	0.00000	0.00000	0.00000	0.00000	0.00000	;	HM73-	C7M-	C7-	C8
18	23	25	26	3	30.33400	0.00000	-30.33400	0.00000	0.00000	0.00000	;	C8-	C9-	C9A-	N10
19	18	23	24	3	30.33400	0.00000	-30.33400	0.00000	0.00000	0.00000	;	C8M-	C8-	C9-	H9
19	18	23	25	3	30.33400	0.00000	-30.33400	0.00000	0.00000	0.00000	;	C8M-	C8-	C9-	C9A
20	19	18	23	3	0.00000	0.00000	0.00000	0.00000	0.00000	0.00000	;	HM81-	C8M-	C8-	C9
21	19	18	23	3	0.00000	0.00000	0.00000	0.00000	0.00000	0.00000	;	HM82-	C8M-	C8-	C9
22	19	18	23	3	0.00000	0.00000	0.00000	0.00000	0.00000	0.00000	;	HM83-	C8M-	C8-	C9
23	25	26	27	3	2.51040	0.00000	-2.51040	0.00000	0.00000	0.00000	;	C9-	C9A-	N10-	C10
23	25	26	28	3	2.51040	0.00000	-2.51040	0.00000	0.00000	0.00000	;	C9-	C9A-	N10-	C1'
24	23	25	26	3	30.33400	0.00000	-30.33400	0.00000	0.00000	0.00000	;	H9-	C9-	C9A-	N10
25	26	28	29	3	0.00000	0.00000	0.00000	0.00000	0.00000	0.00000	;	C9A-	N10-	C1'-	H1'1
25	26	28	30	3	0.00000	0.00000	0.00000	0.00000	0.00000	0.00000	;	C9A-	N10-	C1'-	H1'2
25	26	28	31	3	0.00000	0.00000	0.00000	0.00000	0.00000	0.00000	;	C9A-	N10-	C1'-	C2'
26	28	31	32	3	0.65084	1.95253	0.00000	-2.60338	0.00000	0.00000	;	N10-	C1'-	C2'-	H2'
26	28	31	33	3	0.65084	1.95253	0.00000	-2.60338	0.00000	0.00000	;	N10-	C1'-	C2'-	O2'
26	28	31	35	3	0.65084	1.95253	0.00000	-2.60338	0.00000	0.00000	;	N10-	C1'-	C2'-	C3'
27	1	2	3	3	33.47200	0.00000	-33.47200	0.00000	0.00000	0.00000	;	C10-	N1-	C2-	O2
27	1	2	4	3	33.47200	0.00000	-33.47200	0.00000	0.00000	0.00000	;	C10-	N1-	C2-	N3
27	26	28	29	3	0.00000	0.00000	0.00000	0.00000	0.00000	0.00000	;	C10-	N10-	C1'-	H1'1
27	26	28	30	3	0.00000	0.00000	0.00000	0.00000	0.00000	0.00000	;	C10-	N10-	C1'-	H1'2
27	26	28	31	3	0.00000	0.00000	0.00000	0.00000	0.00000	0.00000	;	C10-	N10-	C1'-	C2'
28	31	33	34	3	1.71544	0.96232	0.00000	-2.67776	0.00000	0.00000	;	C1'-	C2'-	O2'-	HO2'
28	31	35	36	3	0.65084	1.95253	0.00000	-2.60338	0.00000	0.00000	;	C1'-	C2'-	C3'-	H3'
28	31	35	37	3	0.65084	1.95253	0.00000	-2.60338	0.00000	0.00000	;	C1'-	C2'-	C3'-	O3'
28	31	35	39	3	3.68192	3.09616	-2.09200	-3.01248	0.00000	0.00000	;	C1'-	C2'-	C3'-	C4'
29	28	31	32	3	0.65084	1.95253	0.00000	-2.60338	0.00000	0.00000	;	H1'1-	C1'-	C2'-	H2'
29	28	31	33	3	1.04600	-1.04600	0.00000	0.00000	0.00000	0.00000	;	H1'1-	C1'-	C2'-	O2'
29	28	31	35	3	0.65084	1.95253	0.00000	-2.60338	0.00000	0.00000	;	H1'1-	C1'-	C2'-	C3'
30	28	31	32	3	0.65084	1.95253	0.00000	-2.60338	0.00000	0.00000	;	H1'2-	C1'-	C2'-	H2'
30	28	31	33	3	1.04600	-1.04600	0.00000	0.00000	0.00000	0.00000	;	H1'2-	C1'-	C2'-	O2'
30	28	31	35	3	0.65084	1.95253	0.00000	-2.60338	0.00000	0.00000	;	H1'2-	C1'-	C2'-	C3'
31	35	37	38	3	1.71544	0.96232	0.00000	-2.67776	0.00000	0.00000	;	C2'-	C3'-	O3'-	HO3'
31	35	39	40	3	0.65084	1.95253	0.00000	-2.60338	0.00000	0.00000	;	C2'-	C3'-	C4'-	H4'
31	35	39	41	3	0.65084	1.95253	0.00000	-2.60338	0.00000	0.00000	;	C2'-	C3'-	C4'-	O4'
31	35	39	43	3	3.68192	3.09616	-2.09200	-3.01248	0.00000	0.00000	;	C2'-	C3'-	C4'-	C5'
32	31	33	34	3	0.69733	2.09200	0.00000	-2.78933	0.00000	0.00000	;	H2'-	C2'-	O2'-	HO2'
32	31	35	36	3	0.65084	1.95253	0.00000	-2.60338	0.00000	0.00000	;	H2'-	C2'-	C3'-	H3'
32	31	35	37	3	1.04600	-1.04600	0.00000	0.00000	0.00000	0.00000	;	H2'-	C2'-	C3'-	O3'
32	31	35	39	3	0.65084	1.95253	0.00000	-2.60338	0.00000	0.00000	;	H2'-	C2'-	C3'-	C4'
33	31	35	36	3	1.04600	-1.04600	0.00000	0.00000	0.00000	0.00000	;	O2'-	C2'-	C3'-	H3'
33	31	35	37	3	0.60250	1.80749	9.83240	-2.40998	0.00000	0.00000	;	O2'-	C2'-	C3'-	O3'
33	31	35	39	3	0.65084	1.95253	0.00000	-2.60338	0.00000	0.00000	;	O2'-	C2'-	C3'-	C4'
34	33	31	35	3	1.71544	0.96232	0.00000	-2.67776	0.00000	0.00000	;	HO2'-	O2'-	C2'-	C3'
35	39	41	42	3	1.71544	0.96232	0.00000	-2.67776	0.00000	0.00000	;	C3'-	C4'-	O4'-	HO4'
35	39	43	44	3	0.65084	1.95253	0.00000	-2.60338	0.00000	0.00000	;	C3'-	C4'-	C5'-	H5'1
35	39	43	45	3	0.65084	1.95253	0.00000	-2.60338	0.00000	0.00000	;	C3'-	C4'-	C5'-	H5'2
35	39	43	46	3	0.65084	1.95253	0.00000	-2.60338	0.00000	0.00000	;	C3'-	C4'-	C5'-	O5'
36	35	37	38	3	0.69733	2.09200	0.00000	-2.78933	0.00000	0.00000	;	H3'-	C3'-	O3'-	HO3'
36	35	39	40	3	0.65084	1.95253	0.00000	-2.60338	0.00000	0.00000	;	H3'-	C3'-	C4'-	H4'
36	35	39	41	3	1.04600	-1.04600	0.00000	0.00000	0.00000	0.00000	;	H3'-	C3'-	C4'-	O4'
36	35	39	43	3	0.65084	1.95253	0.00000	-2.60338	0.00000	0.00000	;	H3'-	C3'-	C4'-	C5'
37	35	39	40	3	1.04600	-1.04600	0.00000	0.00000	0.00000	0.00000	;	O3'-	C3'-	C4'-	H4'
37	35	39	41	3	0.60250	1.80749	9.83240	-2.40998	0.00000	0.00000	;	O3'-	C3'-	C4'-	O4'
37	35	39	43	3	0.65084	1.95253	0.00000	-2.60338	0.00000	0.00000	;	O3'-	C3'-	C4'-	C5'
38	37	35	39	3	1.71544	0.96232	0.00000	-2.67776	0.00000	0.00000	;	HO3'-	O3'-	C3'-	C4'

```
   39     43     46     47      3   1.60387    4.81160    0.00000   -6.41547    0.00000    0.00000 ;    C4'-   C5'-   O5'-      P
   40     39     41     42      3   0.69733    2.09200    0.00000   -2.78933    0.00000    0.00000 ;    H4'-   C4'-   O4'-   HO4'
   40     39     43     44      3   0.65084    1.95253    0.00000   -2.60338    0.00000    0.00000 ;    H4'-   C4'-   C5'-   H5'1
   40     39     43     45      3   0.65084    1.95253    0.00000   -2.60338    0.00000    0.00000 ;    H4'-   C4'-   C5'-   H5'2
   40     39     43     46      3   1.04600   -1.04600    0.00000    0.00000    0.00000    0.00000 ;    H4'-   C4'-   C5'-    O5'
   41     39     43     44      3   1.04600   -1.04600    0.00000    0.00000    0.00000    0.00000 ;    O4'-   C4'-   C5'-   H5'1
   41     39     43     45      3   1.04600   -1.04600    0.00000    0.00000    0.00000    0.00000 ;    O4'-   C4'-   C5'-   H5'2
   41     39     43     46      3   0.60250    1.80749    9.83240   -2.40998    0.00000    0.00000 ;    O4'-   C4'-   C5'-    O5'
   42     41     39     43      3   1.71544    0.96232    0.00000   -2.67776    0.00000    0.00000 ;   HO4'-   O4'-   C4'-    C5'
   43     46     47     48      3   0.00000    0.00000    6.69440    0.00000    0.00000    0.00000 ;    C5'-   O5'-     P-    O1P
   43     46     47     49      3   0.00000    0.00000    6.69440    0.00000    0.00000    0.00000 ;    C5'-   O5'-     P-    O2P
   43     46     47     50      3   0.00000    0.00000    6.69440    0.00000    0.00000    0.00000 ;    C5'-   O5'-     P-    O3P
   44     43     46     47      3   1.60387    4.81160    0.00000   -6.41547    0.00000    0.00000 ;   H5'1-   C5'-   O5'-      P
   45     43     46     47      3   1.60387    4.81160    0.00000   -6.41547    0.00000    0.00000 ;   H5'2-   C5'-   O5'-      P

[ dihedrals ] ; impropers
; treated as propers in GROMACS to use correct AMBER analytical function
;    i      j      k      l   func   phase     kd      pn
     1     27     26      8      1   180.00   4.60240   2 ;     N1-   C10-   N10-   C4A
     2      6      4      5      1   180.00   4.60240   2 ;     C2-    C4-    N3-   HN3
     4      1      2      3      1   180.00  43.93200   2 ;     N3-    N1-    C2-    O2
     6     27      8      9      1   180.00   4.60240   2 ;     C4-   C10-   C4A-    N5
     8      4      6      7      1   180.00  43.93200   2 ;    C4A-    N3-    C4-    O4
    10     13     11     12      1   180.00   4.60240   2 ;    C5A-    C7-    C6-    H6
    10     23     25     26      1   180.00   4.60240   2 ;    C5A-    C9-   C9A-   N10
    11     18     13     14      1   180.00   4.60240   2 ;     C6-    C8-    C7-   C7M
    11     25     10      9      1   180.00   4.60240   2 ;     C6-   C9A-   C5A-    N5
    18     25     23     24      1   180.00   4.60240   2 ;     C8-   C9A-    C9-    H9
    19     13     18     23      1   180.00   4.60240   2 ;    C8M-    C7-    C8-    C9
    28     25     26     27      1   180.00   4.60240   2 ;    C1'-   C9A-   N10-   C10
```

PreQ1 Parameterization

```
[ atomtypes ]
;name   bond_type     mass     charge   ptype   sigma         epsilon       Amb
 n        n          0.00000  0.00000   A     3.25000e-01   7.11280e-01 ; 1.82  0.1700
 cc       cc         0.00000  0.00000   A     3.39967e-01   3.59824e-01 ; 1.91  0.0860
 nd       nd         0.00000  0.00000   A     3.25000e-01   7.11280e-01 ; 1.82  0.1700
 cd       cd         0.00000  0.00000   A     3.39967e-01   3.59824e-01 ; 1.91  0.0860
 c        c          0.00000  0.00000   A     3.39967e-01   3.59824e-01 ; 1.91  0.0860
 o        o          0.00000  0.00000   A     2.95992e-01   8.78640e-01 ; 1.66  0.2100
 c3       c3         0.00000  0.00000   A     3.39967e-01   4.57730e-01 ; 1.91  0.1094
 n4       n4         0.00000  0.00000   A     3.25000e-01   7.11280e-01 ; 1.82  0.1700
 na       na         0.00000  0.00000   A     3.25000e-01   7.11280e-01 ; 1.82  0.1700
 nh       nh         0.00000  0.00000   A     3.25000e-01   7.11280e-01 ; 1.82  0.1700
 hn       hn         0.00000  0.00000   A     1.06908e-01   6.56888e-02 ; 0.60  0.0157
 hx       hx         0.00000  0.00000   A     1.95998e-01   6.56888e-02 ; 1.10  0.0157
 h4       h4         0.00000  0.00000   A     2.51055e-01   6.27600e-02 ; 1.41  0.0150

[ moleculetype ]
;name            nrexcl
 PRF               3

[ atoms ]
;   nr  type  resi  res  atom  cgnr     charge      mass       ; qtot   bond_type
     1    n     1   PRF    N1    1    -0.812223     14.01000 ; qtot -0.812
     2   cc     1   PRF    C2    2     1.113785     12.01000 ; qtot 0.302
     3   nd     1   PRF    N3    3    -0.779199     14.01000 ; qtot -0.478
     4   cd     1   PRF    C4    4     0.507226     12.01000 ; qtot 0.030
     5   cc     1   PRF    C5    5    -0.310980     12.01000 ; qtot -0.281
     6    c     1   PRF    C6    6     0.771100     12.01000 ; qtot 0.490
     7    o     1   PRF    O6    7    -0.655883     16.00000 ; qtot -0.166
     8   cc     1   PRF    C7    8    -0.088930     12.01000 ; qtot -0.255
     9   c3     1   PRF   C10    9     0.165983     12.01000 ; qtot -0.089
    10   n4     1   PRF   N11   10    -0.619529     14.01000 ; qtot -0.709
    11   cd     1   PRF    C8   11    -0.277226     12.01000 ; qtot -0.986
    12   na     1   PRF    N9   12    -0.289640     14.01000 ; qtot -1.276
    13   nh     1   PRF    N2   13    -1.136486     14.01000 ; qtot -2.412
    14   hn     1   PRF    H1   14     0.451407      1.00800 ; qtot -1.961
    15   hx     1   PRF  H101   15     0.092254      1.00800 ; qtot -1.868
    16   hx     1   PRF  H102   16     0.092254      1.00800 ; qtot -1.776
    17   hn     1   PRF  H111   17     0.359643      1.00800 ; qtot -1.416
    18   hn     1   PRF  H112   18     0.359643      1.00800 ; qtot -1.057
    19   hn     1   PRF  H113   19     0.415401      1.00800 ; qtot -0.641
    20   h4     1   PRF    H8   20     0.253938      1.00800 ; qtot -0.387
    21   hn     1   PRF    H9   21     0.389385      1.00800 ; qtot 0.002
    22   hn     1   PRF   H21   22     0.499037      1.00800 ; qtot 0.501
    23   hn     1   PRF   H22   23     0.499037      1.00800 ; qtot 1.000

[ bonds ]
;   ai     aj funct   r             k
     1      2   1    1.3807e-01    3.5572e+05 ;     N1 - C2
     1      6   1    1.3789e-01    3.5782e+05 ;     N1 - C6
     1     14   1    1.0129e-01    3.3740e+05 ;     N1 - H1
     2      3   1    1.3172e-01    4.3965e+05 ;     C2 - N3
     2     13   1    1.3735e-01    3.6418e+05 ;     C2 - N2
     3      4   1    1.3694e-01    3.6911e+05 ;     N3 - C4
     4      5   1    1.3729e-01    4.1915e+05 ;     C4 - C5
     4     12   1    1.3802e-01    3.5631e+05 ;     C4 - N9
     5      6   1    1.4676e-01    3.1045e+05 ;     C5 - C6
     5      8   1    1.4278e-01    3.5129e+05 ;     C5 - C7
     6      7   1    1.2183e-01    5.3363e+05 ;     C6 - O6
     8      9   1    1.5015e-01    2.8016e+05 ;     C7 - C10
     8     11   1    1.3729e-01    4.1915e+05 ;     C7 - C8
     9     10   1    1.5110e-01    2.3707e+05 ;    C10 - N11
     9     15   1    1.0910e-01    2.8342e+05 ;    C10 - H101
     9     16   1    1.0910e-01    2.8342e+05 ;    C10 - H102
    10     17   1    1.0304e-01    3.1229e+05 ;    N11 - H111
    10     18   1    1.0304e-01    3.1229e+05 ;    N11 - H112
    10     19   1    1.0304e-01    3.1229e+05 ;    N11 - H113
    11     12   1    1.3802e-01    3.5631e+05 ;     C8 - N9
    11     20   1    1.0817e-01    2.9455e+05 ;     C8 - H8
```

```
    12     21   1    1.0100e-01    3.4175e+05 ;      N9 - H9
    13     22   1    1.0121e-01    3.3857e+05 ;      N2 - H21
    13     23   1    1.0121e-01    3.3857e+05 ;      N2 - H22

[ pairs ]
;   ai     aj    funct
     1      4      1 ;     N1 - C4
     1      8      1 ;     N1 - C7
     1     22      1 ;     N1 - H21
     1     23      1 ;     N1 - H22
     2      5      1 ;     C2 - C5
     2      7      1 ;     C2 - O6
     2     12      1 ;     C2 - N9
     3      8      1 ;     N3 - C7
     3     11      1 ;     N3 - C8
     3     21      1 ;     N3 - H9
     3     22      1 ;     N3 - H21
     3     23      1 ;     N3 - H22
     4      7      1 ;     C4 - O6
     4      9      1 ;     C4 - C10
     4     13      1 ;     C4 - N2
     4     20      1 ;     C4 - H8
     5     10      1 ;     C5 - N11
     5     15      1 ;     C5 - H101
     5     16      1 ;     C5 - H102
     5     20      1 ;     C5 - H8
     5     21      1 ;     C5 - H9
     6      3      1 ;     C6 - N3
     6      9      1 ;     C6 - C10
     6     11      1 ;     C6 - C8
     6     12      1 ;     C6 - N9
     6     13      1 ;     C6 - N2
     7      8      1 ;     O6 - C7
     8     17      1 ;     C7 - H111
     8     18      1 ;     C7 - H112
     8     19      1 ;     C7 - H113
     8     21      1 ;     C7 - H9
     9     12      1 ;    C10 - N9
     9     20      1 ;    C10 - H8
    10     11      1 ;    N11 - C8
    11     15      1 ;     C8 - H101
    11     16      1 ;     C8 - H102
    14      3      1 ;     H1 - N3
    14      5      1 ;     H1 - C5
    14      7      1 ;     H1 - O6
    14     13      1 ;     H1 - N2
    15     17      1 ;   H101 - H111
    15     18      1 ;   H101 - H112
    15     19      1 ;   H101 - H113
    16     17      1 ;   H102 - H111
    16     18      1 ;   H102 - H112
    16     19      1 ;   H102 - H113
    20     21      1 ;     H8 - H9

[ angles ]
;   ai     aj     ak    funct   theta         cth
     1      2      3      1    1.2300e+02    5.9831e+02 ;     N1 - C2     - N3
     1      2     13      1    1.1694e+02    6.0166e+02 ;     N1 - C2     - N2
     1      6      5      1    1.1270e+02    5.7823e+02 ;     N1 - C6     - C5
     1      6      7      1    1.2305e+02    6.2091e+02 ;     N1 - C6     - O6
     2      1      6      1    1.2327e+02    5.4141e+02 ;     C2 - N1     - C6
     2      1     14      1    1.1926e+02    4.0083e+02 ;     C2 - N1     - H1
     2      3      4      1    1.0549e+02    6.0082e+02 ;     C2 - N3     - C4
     2     13     22      1    1.1563e+02    4.0920e+02 ;     C2 - N2     - H21
     2     13     23      1    1.1563e+02    4.0920e+02 ;     C2 - N2     - H22
     3      2     13      1    1.2065e+02    6.0584e+02 ;     N3 - C2     - N2
     3      4      5      1    1.1165e+02    6.0417e+02 ;     N3 - C4     - C5
     3      4     12      1    1.2195e+02    5.9078e+02 ;     N3 - C4     - N9
     4      5      6      1    1.2135e+02    5.4476e+02 ;     C4 - C5     - C6
     4      5      8      1    1.1419e+02    5.7070e+02 ;     C4 - C5     - C7
     4     12     11      1    1.0990e+02    5.7321e+02 ;     C4 - N9     - C8
```

```
     4     12     21      1     1.2550e+02    3.9162e+02 ;      C4 - N9     - H9
     5      4     12      1     1.0699e+02    6.1421e+02 ;      C5 - C4     - N9
     5      6      7      1     1.2393e+02    5.7823e+02 ;      C5 - C6     - O6
     5      8      9      1     1.1597e+02    5.4057e+02 ;      C5 - C7     - C10
     5      8     11      1     1.1419e+02    5.7070e+02 ;      C5 - C7     - C8
     6      1     14      1     1.1755e+02    4.0417e+02 ;      C6 - N1     - H1
     6      5      8      1     1.2269e+02    5.3220e+02 ;      C6 - C5     - C7
     8      9     10      1     1.1558e+02    5.4057e+02 ;      C7 - C10    - N11
     8      9     15      1     1.1101e+02    3.9413e+02 ;      C7 - C10    - H101
     8      9     16      1     1.1101e+02    3.9413e+02 ;      C7 - C10    - H102
     8     11     12      1     1.0699e+02    6.1421e+02 ;      C7 - C8     - N9
     8     11     20      1     1.2848e+02    3.9581e+02 ;      C7 - C8     - H8
     9      8     11      1     1.1945e+02    5.4141e+02 ;     C10 - C7     - C8
     9     10     17      1     1.1011e+02    3.8409e+02 ;     C10 - N11    - H111
     9     10     18      1     1.1011e+02    3.8409e+02 ;     C10 - N11    - H112
     9     10     19      1     1.1011e+02    3.8409e+02 ;     C10 - N11    - H113
    10      9     15      1     1.0801e+02    4.0668e+02 ;     N11 - C10    - H101
    10      9     16      1     1.0801e+02    4.0668e+02 ;     N11 - C10    - H102
    11     12     21      1     1.2550e+02    3.9162e+02 ;      C8 - N9     - H9
    12     11     20      1     1.2053e+02    4.1673e+02 ;      N9 - C8     - H8
    15      9     16      1     1.0975e+02    3.2803e+02 ;    H101 - C10    - H102
    17     10     18      1     1.0830e+02    3.3974e+02 ;    H111 - N11    - H112
    17     10     19      1     1.0830e+02    3.3974e+02 ;    H111 - N11    - H113
    18     10     19      1     1.0830e+02    3.3974e+02 ;    H112 - N11    - H113
    22     13     23      1     1.1512e+02    3.3556e+02 ;     H21 - N2     - H22

[ dihedrals ] ; propers
; treated as RBs in GROMACS to use combine multiple AMBER torsions per quartet
;    i      j      k      l   func     C0          C1          C2          C3          C4          C5
     1      2      3      4      3   39.74800    0.00000  -39.74800    0.00000    0.00000    0.00000 ;    N1-   C2-   N3-    C4
     1      2     13     22      3    8.78640    0.00000   -8.78640    0.00000    0.00000    0.00000 ;    N1-   C2-   N2-   H21
     1      2     13     23      3    8.78640    0.00000   -8.78640    0.00000    0.00000    0.00000 ;    N1-   C2-   N2-   H22
     1      6      5      4      3   24.05800    0.00000  -24.05800    0.00000    0.00000    0.00000 ;    N1-   C6-   C5-    C4
     1      6      5      8      3   24.05800    0.00000  -24.05800    0.00000    0.00000    0.00000 ;    N1-   C6-   C5-    C7
     2      1      6      5      3   20.92000    0.00000  -20.92000    0.00000    0.00000    0.00000 ;    C2-   N1-   C6-    C5
     2      1      6      7      3   20.92000    0.00000  -20.92000    0.00000    0.00000    0.00000 ;    C2-   N1-   C6-    O6
     2      3      4      5      3   39.74800    0.00000  -39.74800    0.00000    0.00000    0.00000 ;    C2-   N3-   C4-    C5
     2      3      4     12      3   39.74800    0.00000  -39.74800    0.00000    0.00000    0.00000 ;    C2-   N3-   C4-    N9
     3      2     13     22      3    8.78640    0.00000   -8.78640    0.00000    0.00000    0.00000 ;    N3-   C2-   N2-   H21
     3      2     13     23      3    8.78640    0.00000   -8.78640    0.00000    0.00000    0.00000 ;    N3-   C2-   N2-   H22
     3      4      5      6      3   33.47200    0.00000  -33.47200    0.00000    0.00000    0.00000 ;    N3-   C4-   C5-    C6
     3      4      5      8      3   33.47200    0.00000  -33.47200    0.00000    0.00000    0.00000 ;    N3-   C4-   C5-    C7
     3      4     12     11      3   14.22560    0.00000  -14.22560    0.00000    0.00000    0.00000 ;    N3-   C4-   N9-    C8
     3      4     12     21      3   14.22560    0.00000  -14.22560    0.00000    0.00000    0.00000 ;    N3-   C4-   N9-    H9
     4      3      2     13      3   39.74800    0.00000  -39.74800    0.00000    0.00000    0.00000 ;    C4-   N3-   C2-    N2
     4      5      6      7      3   24.05800    0.00000  -24.05800    0.00000    0.00000    0.00000 ;    C4-   C5-   C6-    O6
     4      5      8      9      3   33.47200    0.00000  -33.47200    0.00000    0.00000    0.00000 ;    C4-   C5-   C7-   C10
     4      5      8     11      3   33.47200    0.00000  -33.47200    0.00000    0.00000    0.00000 ;    C4-   C5-   C7-    C8
     4     12     11      8      3   14.22560    0.00000  -14.22560    0.00000    0.00000    0.00000 ;    C4-   N9-   C8-    C7
     4     12     11     20      3   14.22560    0.00000  -14.22560    0.00000    0.00000    0.00000 ;    C4-   N9-   C8-    H8
     5      4     12     11      3   14.22560    0.00000  -14.22560    0.00000    0.00000    0.00000 ;    C5-   C4-   N9-    C8
     5      4     12     21      3   14.22560    0.00000  -14.22560    0.00000    0.00000    0.00000 ;    C5-   C4-   N9-    H9
     5      8      9     10      3    0.00000    0.00000    0.00000    0.00000    0.00000    0.00000 ;    C5-   C7-  C10-   N11
     5      8      9     15      3    0.00000    0.00000    0.00000    0.00000    0.00000    0.00000 ;    C5-   C7-  C10-  H101
     5      8      9     16      3    0.00000    0.00000    0.00000    0.00000    0.00000    0.00000 ;    C5-   C7-  C10-  H102
     5      8     11     12      3   33.47200    0.00000  -33.47200    0.00000    0.00000    0.00000 ;    C5-   C7-   C8-    N9
     5      8     11     20      3   33.47200    0.00000  -33.47200    0.00000    0.00000    0.00000 ;    C5-   C7-   C8-    H8
     6      1      2      3      3   13.80720    0.00000  -13.80720    0.00000    0.00000    0.00000 ;    C6-   N1-   C2-    N3
     6      1      2     13      3   13.80720    0.00000  -13.80720    0.00000    0.00000    0.00000 ;    C6-   N1-   C2-    N2
     6      5      4     12      3   33.47200    0.00000  -33.47200    0.00000    0.00000    0.00000 ;    C6-   C5-   C4-    N9
     6      5      8      9      3   33.47200    0.00000  -33.47200    0.00000    0.00000    0.00000 ;    C6-   C5-   C7-   C10
     6      5      8     11      3   33.47200    0.00000  -33.47200    0.00000    0.00000    0.00000 ;    C6-   C5-   C7-    C8
     7      6      5      8      3   24.05800    0.00000  -24.05800    0.00000    0.00000    0.00000 ;    O6-   C6-   C5-    C7
     8      5      4     12      3   33.47200    0.00000  -33.47200    0.00000    0.00000    0.00000 ;    C7-   C5-   C4-    N9
     8      9     10     17      3    0.65084    1.95253    0.00000   -2.60338    0.00000    0.00000 ;    C7-  C10-  N11-  H111
     8      9     10     18      3    0.65084    1.95253    0.00000   -2.60338    0.00000    0.00000 ;    C7-  C10-  N11-  H112
     8      9     10     19      3    0.65084    1.95253    0.00000   -2.60338    0.00000    0.00000 ;    C7-  C10-  N11-  H113
     8     11     12     21      3   14.22560    0.00000  -14.22560    0.00000    0.00000    0.00000 ;    C7-   C8-   N9-    H9
     9      8     11     12      3   33.47200    0.00000  -33.47200    0.00000    0.00000    0.00000 ;   C10-   C7-   C8-    N9
     9      8     11     20      3   33.47200    0.00000  -33.47200    0.00000    0.00000    0.00000 ;   C10-   C7-   C8-    H8
    10      9      8     11      3    0.00000    0.00000    0.00000    0.00000    0.00000    0.00000 ;   N11-  C10-   C7-    C8
    11      8      9     15      3    0.00000    0.00000    0.00000    0.00000    0.00000    0.00000 ;    C8-   C7-  C10-  H101
    11      8      9     16      3    0.00000    0.00000    0.00000    0.00000    0.00000    0.00000 ;    C8-   C7-  C10-  H102
    14      1      2      3      3   13.80720    0.00000  -13.80720    0.00000    0.00000    0.00000 ;    H1-   N1-   C2-    N3
    14      1      2     13      3   13.80720    0.00000  -13.80720    0.00000    0.00000    0.00000 ;    H1-   N1-   C2-    N2
    14      1      6      5      3   20.92000    0.00000  -20.92000    0.00000    0.00000    0.00000 ;    H1-   N1-   C6-    C5
    14      1      6      7      3   29.28800   -8.36800  -20.92000    0.00000    0.00000    0.00000 ;    H1-   N1-   C6-    O6
    15      9     10     17      3    0.65084    1.95253    0.00000   -2.60338    0.00000    0.00000 ;  H101-  C10-  N11-  H111
    15      9     10     18      3    0.65084    1.95253    0.00000   -2.60338    0.00000    0.00000 ;  H101-  C10-  N11-  H112
    15      9     10     19      3    0.65084    1.95253    0.00000   -2.60338    0.00000    0.00000 ;  H101-  C10-  N11-  H113
    16      9     10     17      3    0.65084    1.95253    0.00000   -2.60338    0.00000    0.00000 ;  H102-  C10-  N11-  H111
    16      9     10     18      3    0.65084    1.95253    0.00000   -2.60338    0.00000    0.00000 ;  H102-  C10-  N11-  H112
    16      9     10     19      3    0.65084    1.95253    0.00000   -2.60338    0.00000    0.00000 ;  H102-  C10-  N11-  H113
    20     11     12     21      3   14.22560    0.00000  -14.22560    0.00000    0.00000    0.00000 ;    H8-   C8-   N9-    H9

[ dihedrals ] ; impropers
; treated as propers in GROMACS to use correct AMBER analytical function
;    i      j      k      l   func   phase     kd      pn
     1      3      2     13      1   180.00   4.60240   2 ;     N1-    N3-    C2-    N2
```

2	22	13	23	1	180.00	4.60240	2	;	C2-	H21-	N2-	H22
4	11	12	21	1	180.00	4.60240	2	;	C4-	C8-	N9-	H9
5	1	6	7	1	180.00	43.93200	2	;	C5-	N1-	C6-	O6
5	12	4	3	1	180.00	4.60240	2	;	C5-	N9-	C4-	N3
6	8	5	4	1	180.00	4.60240	2	;	C6-	C7-	C5-	C4
8	20	11	12	1	180.00	4.60240	2	;	C7-	H8-	C8-	N9
9	5	8	11	1	180.00	4.60240	2	;	C10-	C5-	C7-	C8
14	1	2	6	1	180.00	4.60240	2	;	H1-	N1-	C2-	C6

Adenine Parameterization

```
[ atomtypes ]
;name   bond_type     mass     charge   ptype   sigma         epsilon       Amb
 na       na          0.00000  0.00000   A     3.25000e-01   7.11280e-01 ; 1.82  0.1700
 cc       cc          0.00000  0.00000   A     3.39967e-01   3.59824e-01 ; 1.91  0.0860
 nd       nd          0.00000  0.00000   A     3.25000e-01   7.11280e-01 ; 1.82  0.1700
 ca       ca          0.00000  0.00000   A     3.39967e-01   3.59824e-01 ; 1.91  0.0860
 nh       nh          0.00000  0.00000   A     3.25000e-01   7.11280e-01 ; 1.82  0.1700
 nb       nb          0.00000  0.00000   A     3.25000e-01   7.11280e-01 ; 1.82  0.1700
 hn       hn          0.00000  0.00000   A     1.06908e-01   6.56888e-02 ; 0.60  0.0157
 h5       h5          0.00000  0.00000   A     2.42146e-01   6.27600e-02 ; 1.36  0.0150

[ moleculetype ]
;name            nrexcl
 ADE              3

[ atoms ]
;   nr  type  resi  res  atom  cgnr     charge      mass       ; qtot   bond_type
     1   na     1   ADE    N9    1    -0.680395     14.01000 ; qtot -0.680
     2   cc     1   ADE    C8    2     0.320807     12.01000 ; qtot -0.360
     3   nd     1   ADE    N7    3    -0.601205     14.01000 ; qtot -0.961
     4   ca     1   ADE    C5    4    -0.072394     12.01000 ; qtot -1.033
     5   ca     1   ADE    C6    5     0.858578     12.01000 ; qtot -0.175
     6   nh     1   ADE    N6    6    -1.070396     14.01000 ; qtot -1.245
     7   nb     1   ADE    N1    7    -0.805178     14.01000 ; qtot -2.050
     8   ca     1   ADE    C2    8     0.506323     12.01000 ; qtot -1.544
     9   nb     1   ADE    N3    9    -0.788317     14.01000 ; qtot -2.332
    10   ca     1   ADE    C4   10     0.731612     12.01000 ; qtot -1.601
    11   hn     1   ADE    H9   11     0.435831      1.00800 ; qtot -1.165
    12   h5     1   ADE    H8   12     0.136268      1.00800 ; qtot -1.028
    13   hn     1   ADE   H61   13     0.463833      1.00800 ; qtot -0.565
    14   hn     1   ADE   H62   14     0.463833      1.00800 ; qtot -0.101
    15   h5     1   ADE    H2   15     0.100798      1.00800 ; qtot 0.000

[ bonds ]
;   ai     aj funct   r             k
     1      2   1    1.3802e-01    3.5631e+05 ;     N9 - C8
     1     10   1    1.3840e-01    3.5187e+05 ;     N9 - C4
     1     11   1    1.0100e-01    3.4175e+05 ;     N9 - H9
     2      3   1    1.3172e-01    4.3965e+05 ;     C8 - N7
     2     12   1    1.0819e-01    2.9430e+05 ;     C8 - H8
     3      4   1    1.3517e-01    3.9137e+05 ;     N7 - C5
     4      5   1    1.3984e-01    3.8585e+05 ;     C5 - C6
     4     10   1    1.3984e-01    3.8585e+05 ;     C5 - C4
     5      6   1    1.3859e-01    3.4970e+05 ;     C6 - N6
     5      7   1    1.3390e-01    4.0836e+05 ;     C6 - N1
     6     13   1    1.0121e-01    3.3857e+05 ;     N6 - H61
     6     14   1    1.0121e-01    3.3857e+05 ;     N6 - H62
     7      8   1    1.3390e-01    4.0836e+05 ;     N1 - C2
     8      9   1    1.3390e-01    4.0836e+05 ;     C2 - N3
     8     15   1    1.0878e-01    2.8719e+05 ;     C2 - H2
     9     10   1    1.3390e-01    4.0836e+05 ;     N3 - C4

[ pairs ]
;   ai     aj    funct
     1      5      1 ;     N9 - C6
     1      8      1 ;     N9 - C2
     2      5      1 ;     C8 - C6
     2      9      1 ;     C8 - N3
     3      6      1 ;     N7 - N6
     3      7      1 ;     N7 - N1
     3      9      1 ;     N7 - N3
     4      8      1 ;     C5 - C2
     4     12      1 ;     C5 - H8
     4     13      1 ;     C5 - H61
     4     14      1 ;     C5 - H62
     5      9      1 ;     C6 - N3
     5     15      1 ;     C6 - H2
     6      8      1 ;     N6 - C2
     6     10      1 ;     N6 - C4
```

```
     7     10      1 ;     N1 - C4
     7     13      1 ;     N1 - H61
     7     14      1 ;     N1 - H62
    10     12      1 ;     C4 - H8
    10     15      1 ;     C4 - H2
    11      3      1 ;     H9 - N7
    11      4      1 ;     H9 - C5
    11      9      1 ;     H9 - N3
    11     12      1 ;     H9 - H8
[ angles ]
;   ai     aj     ak    funct   theta         cth
     1      2      3      1    1.1222e+02    6.2676e+02 ;     N9 - C8     - N7
     1      2     12      1    1.2155e+02    4.1505e+02 ;     N9 - C8     - H8
     1     10      4      1    1.1834e+02    5.7823e+02 ;     N9 - C4     - C5
     1     10      9      1    1.2709e+02    5.8409e+02 ;     N9 - C4     - N3
     2      1     10      1    1.1315e+02    5.6400e+02 ;     C8 - N9     - C4
     2      1     11      1    1.2550e+02    3.9162e+02 ;     C8 - N9     - H9
     2      3      4      1    1.0488e+02    6.0668e+02 ;     C8 - N7     - C5
     3      2     12      1    1.2552e+02    4.2342e+02 ;     N7 - C8     - H8
     3      4      5      1    1.1972e+02    5.8158e+02 ;     N7 - C5     - C6
     3      4     10      1    1.1972e+02    5.8158e+02 ;     N7 - C5     - C4
     4      5      6      1    1.2095e+02    5.7153e+02 ;     C5 - C6     - N6
     4      5      7      1    1.2294e+02    5.7572e+02 ;     C5 - C6     - N1
     4     10      9      1    1.2294e+02    5.7572e+02 ;     C5 - C4     - N3
     5      4     10      1    1.2002e+02    5.5731e+02 ;     C6 - C5     - C4
     5      6     13      1    1.1607e+02    4.0501e+02 ;     C6 - N6     - H61
     5      6     14      1    1.1607e+02    4.0501e+02 ;     C6 - N6     - H62
     5      7      8      1    1.1722e+02    5.7153e+02 ;     C6 - N1     - C2
     6      5      7      1    1.1694e+02    6.0835e+02 ;     N6 - C6     - N1
     7      8      9      1    1.2726e+02    5.9329e+02 ;     N1 - C2     - N3
     7      8     15      1    1.1582e+02    4.3430e+02 ;     N1 - C2     - H2
     8      9     10      1    1.1722e+02    5.7153e+02 ;     C2 - N3     - C4
     9      8     15      1    1.1582e+02    4.3430e+02 ;     N3 - C2     - H2
    10      1     11      1    1.2554e+02    3.8995e+02 ;     C4 - N9     - H9
    13      6     14      1    1.1512e+02    3.3556e+02 ;    H61 - N6     - H62
[ dihedrals ] ; propers
; treated as RBs in GROMACS to use combine multiple AMBER torsions per quartet
;    i      j      k      l   func     C0         C1         C2         C3         C4         C5
     1      2      3      4      3   39.74800    0.00000  -39.74800    0.00000    0.00000    0.00000 ;     N9-    C8-    N7-    C5
     1     10      4      3      3   30.33400    0.00000  -30.33400    0.00000    0.00000    0.00000 ;     N9-    C4-    C5-    N7
     1     10      4      5      3   30.33400    0.00000  -30.33400    0.00000    0.00000    0.00000 ;     N9-    C4-    C5-    C6
     1     10      9      8      3   40.16640    0.00000  -40.16640    0.00000    0.00000    0.00000 ;     N9-    C4-    N3-    C2
     2      1     10      4      3    2.51040    0.00000   -2.51040    0.00000    0.00000    0.00000 ;     C8-    N9-    C4-    C5
     2      1     10      9      3    2.51040    0.00000   -2.51040    0.00000    0.00000    0.00000 ;     C8-    N9-    C4-    N3
     2      3      4      5      3   40.16640    0.00000  -40.16640    0.00000    0.00000    0.00000 ;     C8-    N7-    C5-    C6
     2      3      4     10      3   40.16640    0.00000  -40.16640    0.00000    0.00000    0.00000 ;     C8-    N7-    C5-    C4
     3      4      5      6      3   30.33400    0.00000  -30.33400    0.00000    0.00000    0.00000 ;     N7-    C5-    C6-    N6
     3      4      5      7      3   30.33400    0.00000  -30.33400    0.00000    0.00000    0.00000 ;     N7-    C5-    C6-    N1
     3      4     10      9      3   30.33400    0.00000  -30.33400    0.00000    0.00000    0.00000 ;     N7-    C5-    C4-    N3
     4      3      2     12      3   39.74800    0.00000  -39.74800    0.00000    0.00000    0.00000 ;     C5-    N7-    C8-    H8
     4      5      6     13      3    8.78640    0.00000   -8.78640    0.00000    0.00000    0.00000 ;     C5-    C6-    N6-   H61
     4      5      6     14      3    8.78640    0.00000   -8.78640    0.00000    0.00000    0.00000 ;     C5-    C6-    N6-   H62
     4      5      7      8      3   40.16640    0.00000  -40.16640    0.00000    0.00000    0.00000 ;     C5-    C6-    N1-    C2
     4     10      9      8      3   40.16640    0.00000  -40.16640    0.00000    0.00000    0.00000 ;     C5-    C4-    N3-    C2
     5      4     10      9      3   30.33400    0.00000  -30.33400    0.00000    0.00000    0.00000 ;     C6-    C5-    C4-    N3
     5      7      8      9      3   40.16640    0.00000  -40.16640    0.00000    0.00000    0.00000 ;     C6-    N1-    C2-    N3
     5      7      8     15      3   40.16640    0.00000  -40.16640    0.00000    0.00000    0.00000 ;     C6-    N1-    C2-    H2
     6      5      4     10      3   30.33400    0.00000  -30.33400    0.00000    0.00000    0.00000 ;     N6-    C6-    C5-    C4
     6      5      7      8      3   40.16640    0.00000  -40.16640    0.00000    0.00000    0.00000 ;     N6-    C6-    N1-    C2
     7      5      4     10      3   30.33400    0.00000  -30.33400    0.00000    0.00000    0.00000 ;     N1-    C6-    C5-    C4
     7      5      6     13      3    8.78640    0.00000   -8.78640    0.00000    0.00000    0.00000 ;     N1-    C6-    N6-   H61
     7      5      6     14      3    8.78640    0.00000   -8.78640    0.00000    0.00000    0.00000 ;     N1-    C6-    N6-   H62
     7      8      9     10      3   40.16640    0.00000  -40.16640    0.00000    0.00000    0.00000 ;     N1-    C2-    N3-    C4
    10      1      2      3      3   14.22560    0.00000  -14.22560    0.00000    0.00000    0.00000 ;     C4-    N9-    C8-    N7
    10      1      2     12      3   14.22560    0.00000  -14.22560    0.00000    0.00000    0.00000 ;     C4-    N9-    C8-    H8
    10      9      8     15      3   40.16640    0.00000  -40.16640    0.00000    0.00000    0.00000 ;     C4-    N3-    C2-    H2
    11      1      2      3      3   14.22560    0.00000  -14.22560    0.00000    0.00000    0.00000 ;     H9-    N9-    C8-    N7
    11      1      2     12      3   14.22560    0.00000  -14.22560    0.00000    0.00000    0.00000 ;     H9-    N9-    C8-    H8
    11      1     10      4      3    2.51040    0.00000   -2.51040    0.00000    0.00000    0.00000 ;     H9-    N9-    C4-    C5
    11      1     10      9      3    2.51040    0.00000   -2.51040    0.00000    0.00000    0.00000 ;     H9-    N9-    C4-    N3

[ dihedrals ] ; impropers
; treated as propers in GROMACS to use correct AMBER analytical function
;    i      j      k      l   func   phase     kd      pn
     4      1     10      9      1   180.00   4.60240   2 ;     C5-    N9-    C4-    N3
     4      7      5      6      1   180.00   4.60240   2 ;     C5-    N1-    C6-    N6
     5     10      4      3      1   180.00   4.60240   2 ;     C6-    C4-    C5-    N7
     5     13      6     14      1   180.00   4.60240   2 ;     C6-   H61-    N6-   H62
    11      1      2     10      1   180.00   4.60240   2 ;     H9-    N9-    C8-    C4
    12      1      2      3      1   180.00   4.60240   2 ;     H8-    N9-    C8-    N7
    15      7      8      9      1   180.00   4.60240   2 ;     H2-    N1-    C2-    N3
```

Citrulline Parameterization

```
[ atomtypes ]
;name   bond_type     mass     charge   ptype   sigma         epsilon       Amb
 c2       c2          0.00000  0.00000   A     3.39967e-01   3.59824e-01 ; 1.91  0.0860
 o        o           0.00000  0.00000   A     2.95992e-01   8.78640e-01 ; 1.66  0.2100
 c3       c3          0.00000  0.00000   A     3.39967e-01   4.57730e-01 ; 1.91  0.1094
 n4       n4          0.00000  0.00000   A     3.25000e-01   7.11280e-01 ; 1.82  0.1700
 n        n           0.00000  0.00000   A     3.25000e-01   7.11280e-01 ; 1.82  0.1700
 c        c           0.00000  0.00000   A     3.39967e-01   3.59824e-01 ; 1.91  0.0860
 hx       hx          0.00000  0.00000   A     1.95998e-01   6.56888e-02 ; 1.10  0.0157
 hn       hn          0.00000  0.00000   A     1.06908e-01   6.56888e-02 ; 0.60  0.0157
 hc       hc          0.00000  0.00000   A     2.64953e-01   6.56888e-02 ; 1.49  0.0157
 h1       h1          0.00000  0.00000   A     2.47135e-01   6.56888e-02 ; 1.39  0.0157

[ moleculetype ]
;name            nrexcl
 CIR              3

[ atoms ]
;   nr  type  resi  res  atom  cgnr     charge      mass       ; qtot   bond_type
     1   c2     1   CIR    C1    1     0.744910     12.01000 ; qtot 0.745
     2    o     1   CIR    O1    2    -0.591837     16.00000 ; qtot 0.153
     3   c3     1   CIR    C2    3     0.387409     12.01000 ; qtot 0.540
     4   n4     1   CIR    N2    4    -1.051487     14.01000 ; qtot -0.511
     5    o     1   CIR    O2    5    -0.613828     16.00000 ; qtot -1.125
     6   c3     1   CIR    C3    6    -0.268176     12.01000 ; qtot -1.393
     7   c3     1   CIR    C4    7    -0.125750     12.01000 ; qtot -1.519
     8   c3     1   CIR    C5    8     0.496207     12.01000 ; qtot -1.023
     9    n     1   CIR    N6    9    -0.884485     14.01000 ; qtot -1.907
    10    c     1   CIR    C7   10     0.970314     12.01000 ; qtot -0.937
    11    o     1   CIR    O7   11    -0.663805     16.00000 ; qtot -1.601
    12    n     1   CIR    N8   12    -0.913355     14.01000 ; qtot -2.514
    13   hx     1   CIR    H2   13    -0.039685      1.00800 ; qtot -2.554
    14   hn     1   CIR    H6   14     0.382044      1.00800 ; qtot -2.172
    15   hn     1   CIR   H21   15     0.425394      1.00800 ; qtot -1.746
    16   hn     1   CIR   H22   16     0.390053      1.00800 ; qtot -1.356
    17   hn     1   CIR   H23   17     0.390053      1.00800 ; qtot -0.966
    18   hc     1   CIR   H31   18     0.089581      1.00800 ; qtot -0.876
    19   hc     1   CIR   H32   19     0.089581      1.00800 ; qtot -0.787
    20   hc     1   CIR   H41   20     0.066823      1.00800 ; qtot -0.720
    21   hc     1   CIR   H42   21     0.066823      1.00800 ; qtot -0.653
    22   h1     1   CIR   H51   22    -0.045110      1.00800 ; qtot -0.698
    23   h1     1   CIR   H52   23    -0.045110      1.00800 ; qtot -0.743
    24   hn     1   CIR   H81   24     0.371716      1.00800 ; qtot -0.372
    25   hn     1   CIR   H82   25     0.371716      1.00800 ; qtot 0.000

[ bonds ]
;   ai     aj funct   r             k
     1      2   1    1.2247e-01    5.2124e+05 ;     C1 - O1
     1      3   1    1.5095e-01    2.7347e+05 ;     C1 - C2
     1      5   1    1.2247e-01    5.2124e+05 ;     C1 - O2
     3      4   1    1.5110e-01    2.3707e+05 ;     C2 - N2
     3      6   1    1.5375e-01    2.5179e+05 ;     C2 - C3
     3     13   1    1.0910e-01    2.8342e+05 ;     C2 - H2
     4     15   1    1.0304e-01    3.1229e+05 ;     N2 - H21
     4     16   1    1.0304e-01    3.1229e+05 ;     N2 - H22
     4     17   1    1.0304e-01    3.1229e+05 ;     N2 - H23
     6      7   1    1.5375e-01    2.5179e+05 ;     C3 - C4
     6     18   1    1.0969e-01    2.7665e+05 ;     C3 - H31
     6     19   1    1.0969e-01    2.7665e+05 ;     C3 - H32
     7      8   1    1.5375e-01    2.5179e+05 ;     C4 - C5
     7     20   1    1.0969e-01    2.7665e+05 ;     C4 - H41
     7     21   1    1.0969e-01    2.7665e+05 ;     C4 - H42
     8      9   1    1.4619e-01    2.7506e+05 ;     C5 - N6
     8     22   1    1.0969e-01    2.7665e+05 ;     C5 - H51
     8     23   1    1.0969e-01    2.7665e+05 ;     C5 - H52
     9     10   1    1.3789e-01    3.5782e+05 ;     N6 - C7
     9     14   1    1.0129e-01    3.3740e+05 ;     N6 - H6
    10     11   1    1.2183e-01    5.3363e+05 ;     C7 - O7
    10     12   1    1.3789e-01    3.5782e+05 ;     C7 - N8
```

```
    12     24   1    1.0129e-01    3.3740e+05 ;     N8 - H81
    12     25   1    1.0129e-01    3.3740e+05 ;     N8 - H82

[ pairs ]
;   ai     aj    funct
     1      7      1 ;     C1 - C4
     1     15      1 ;     C1 - H21
     1     16      1 ;     C1 - H22
     1     17      1 ;     C1 - H23
     1     18      1 ;     C1 - H31
     1     19      1 ;     C1 - H32
     2      4      1 ;     O1 - N2
     2      6      1 ;     O1 - C3
     2     13      1 ;     O1 - H2
     3      8      1 ;     C2 - C5
     3     20      1 ;     C2 - H41
     3     21      1 ;     C2 - H42
     4      7      1 ;     N2 - C4
     4     18      1 ;     N2 - H31
     4     19      1 ;     N2 - H32
     5      4      1 ;     O2 - N2
     5      6      1 ;     O2 - C3
     5     13      1 ;     O2 - H2
     6      9      1 ;     C3 - N6
     6     15      1 ;     C3 - H21
     6     16      1 ;     C3 - H22
     6     17      1 ;     C3 - H23
     6     22      1 ;     C3 - H51
     6     23      1 ;     C3 - H52
     7     10      1 ;     C4 - C7
     7     13      1 ;     C4 - H2
     7     14      1 ;     C4 - H6
     8     11      1 ;     C5 - O7
     8     12      1 ;     C5 - N8
     8     18      1 ;     C5 - H31
     8     19      1 ;     C5 - H32
     9     20      1 ;     N6 - H41
     9     21      1 ;     N6 - H42
     9     24      1 ;     N6 - H81
     9     25      1 ;     N6 - H82
    10     22      1 ;     C7 - H51
    10     23      1 ;     C7 - H52
    11     14      1 ;     O7 - H6
    11     24      1 ;     O7 - H81
    11     25      1 ;     O7 - H82
    12     14      1 ;     N8 - H6
    13     15      1 ;     H2 - H21
    13     16      1 ;     H2 - H22
    13     17      1 ;     H2 - H23
    13     18      1 ;     H2 - H31
    13     19      1 ;     H2 - H32
    14     22      1 ;     H6 - H51
    14     23      1 ;     H6 - H52
    18     20      1 ;    H31 - H41
    18     21      1 ;    H31 - H42
    19     20      1 ;    H32 - H41
    19     21      1 ;    H32 - H42
    20     22      1 ;    H41 - H51
    20     23      1 ;    H41 - H52
    21     22      1 ;    H42 - H51
    21     23      1 ;    H42 - H52

[ angles ]
;   ai     aj     ak    funct   theta         cth
     1      3      4      1    1.1364e+02    5.4308e+02 ;     C1 - C2     - N2
     1      3      6      1    1.1156e+02    5.3053e+02 ;     C1 - C2     - C3
     1      3     13      1    1.1134e+02    3.9162e+02 ;     C1 - C2     - H2
     2      1      3      1    1.2282e+02    5.6819e+02 ;     O1 - C1     - C2
     2      1      5      1    1.2169e+02    6.7111e+02 ;     O1 - C1     - O2
     3      1      5      1    1.2282e+02    5.6819e+02 ;     C2 - C1     - O2
     3      4     15      1    1.1011e+02    3.8409e+02 ;     C2 - N2     - H21
```

```
    3      4     16      1     1.1011e+02    3.8409e+02 ;     C2 - N2     - H22
    3      4     17      1     1.1011e+02    3.8409e+02 ;     C2 - N2     - H23
    3      6      7      1     1.1151e+02    5.2635e+02 ;     C2 - C3     - C4
    3      6     18      1     1.0980e+02    3.8744e+02 ;     C2 - C3     - H31
    3      6     19      1     1.0980e+02    3.8744e+02 ;     C2 - C3     - H32
    4      3      6      1     1.1421e+02    5.3723e+02 ;     N2 - C2     - C3
    4      3     13      1     1.0801e+02    4.0668e+02 ;     N2 - C2     - H2
    6      3     13      1     1.1056e+02    3.8660e+02 ;     C3 - C2     - H2
    6      7      8      1     1.1151e+02    5.2635e+02 ;     C3 - C4     - C5
    6      7     20      1     1.0980e+02    3.8744e+02 ;     C3 - C4     - H41
    6      7     21      1     1.0980e+02    3.8744e+02 ;     C3 - C4     - H42
    7      6     18      1     1.0980e+02    3.8744e+02 ;     C4 - C3     - H31
    7      6     19      1     1.0980e+02    3.8744e+02 ;     C4 - C3     - H32
    7      8      9      1     1.1161e+02    5.5145e+02 ;     C4 - C5     - N6
    7      8     22      1     1.0956e+02    3.8828e+02 ;     C4 - C5     - H51
    7      8     23      1     1.0956e+02    3.8828e+02 ;     C4 - C5     - H52
    8      7     20      1     1.0980e+02    3.8744e+02 ;     C5 - C4     - H41
    8      7     21      1     1.0980e+02    3.8744e+02 ;     C5 - C4     - H42
    8      9     10      1     1.2069e+02    5.3053e+02 ;     C5 - N6     - C7
    8      9     14      1     1.1768e+02    3.8325e+02 ;     C5 - N6     - H6
    9      8     22      1     1.0888e+02    4.1673e+02 ;     N6 - C5     - H51
    9      8     23      1     1.0888e+02    4.1673e+02 ;     N6 - C5     - H52
    9     10     11      1     1.2305e+02    6.2091e+02 ;     N6 - C7     - O7
    9     10     12      1     1.1356e+02    6.1003e+02 ;     N6 - C7     - N8
   10      9     14      1     1.1755e+02    4.0417e+02 ;     C7 - N6     - H6
   10     12     24      1     1.1755e+02    4.0417e+02 ;     C7 - N8     - H81
   10     12     25      1     1.1755e+02    4.0417e+02 ;     C7 - N8     - H82
   11     10     12      1     1.2305e+02    6.2091e+02 ;     O7 - C7     - N8
   15      4     16      1     1.0830e+02    3.3974e+02 ;    H21 - N2     - H22
   15      4     17      1     1.0830e+02    3.3974e+02 ;    H21 - N2     - H23
   16      4     17      1     1.0830e+02    3.3974e+02 ;    H22 - N2     - H23
   18      6     19      1     1.0758e+02    3.2970e+02 ;    H31 - C3     - H32
   20      7     21      1     1.0758e+02    3.2970e+02 ;    H41 - C4     - H42
   22      8     23      1     1.0846e+02    3.2803e+02 ;    H51 - C5     - H52
   24     12     25      1     1.1795e+02    3.3137e+02 ;    H81 - N8     - H82

[ dihedrals ] ; propers
; treated as RBs in GROMACS to use combine multiple AMBER torsions per quartet
;    i      j      k      l   func     C0         C1         C2         C3         C4         C5
     1      3      4     15      3     0.65084    1.95253    0.00000   -2.60338    0.00000    0.00000 ;     C1-    C2-    N2-   H21
     1      3      4     16      3     0.65084    1.95253    0.00000   -2.60338    0.00000    0.00000 ;     C1-    C2-    N2-   H22
     1      3      4     17      3     0.65084    1.95253    0.00000   -2.60338    0.00000    0.00000 ;     C1-    C2-    N2-   H23
     1      3      6      7      3     0.65084    1.95253    0.00000   -2.60338    0.00000    0.00000 ;     C1-    C2-    C3-    C4
     1      3      6     18      3     0.65084    1.95253    0.00000   -2.60338    0.00000    0.00000 ;     C1-    C2-    C3-   H31
     1      3      6     19      3     0.65084    1.95253    0.00000   -2.60338    0.00000    0.00000 ;     C1-    C2-    C3-   H32
     2      1      3      4      3     0.00000    0.00000    0.00000    0.00000    0.00000    0.00000 ;     O1-    C1-    C2-    N2
     2      1      3      6      3     0.00000    0.00000    0.00000    0.00000    0.00000    0.00000 ;     O1-    C1-    C2-    C3
     2      1      3     13      3     0.00000    0.00000    0.00000    0.00000    0.00000    0.00000 ;     O1-    C1-    C2-    H2
     3      6      7      8      3     3.68192    3.09616   -2.09200   -3.01248    0.00000    0.00000 ;     C2-    C3-    C4-    C5
     3      6      7     20      3     0.66944    2.00832    0.00000   -2.67776    0.00000    0.00000 ;     C2-    C3-    C4-   H41
     3      6      7     21      3     0.66944    2.00832    0.00000   -2.67776    0.00000    0.00000 ;     C2-    C3-    C4-   H42
     4      3      6      7      3     0.65084    1.95253    0.00000   -2.60338    0.00000    0.00000 ;     N2-    C2-    C3-    C4
     4      3      6     18      3     0.65084    1.95253    0.00000   -2.60338    0.00000    0.00000 ;     N2-    C2-    C3-   H31
     4      3      6     19      3     0.65084    1.95253    0.00000   -2.60338    0.00000    0.00000 ;     N2-    C2-    C3-   H32
     5      1      3      4      3     0.00000    0.00000    0.00000    0.00000    0.00000    0.00000 ;     O2-    C1-    C2-    N2
     5      1      3      6      3     0.00000    0.00000    0.00000    0.00000    0.00000    0.00000 ;     O2-    C1-    C2-    C3
     5      1      3     13      3     0.00000    0.00000    0.00000    0.00000    0.00000    0.00000 ;     O2-    C1-    C2-    H2
     6      3      4     15      3     0.65084    1.95253    0.00000   -2.60338    0.00000    0.00000 ;     C3-    C2-    N2-   H21
     6      3      4     16      3     0.65084    1.95253    0.00000   -2.60338    0.00000    0.00000 ;     C3-    C2-    N2-   H22
     6      3      4     17      3     0.65084    1.95253    0.00000   -2.60338    0.00000    0.00000 ;     C3-    C2-    N2-   H23
     6      7      8      9      3     0.65084    1.95253    0.00000   -2.60338    0.00000    0.00000 ;     C3-    C4-    C5-    N6
     6      7      8     22      3     0.65084    1.95253    0.00000   -2.60338    0.00000    0.00000 ;     C3-    C4-    C5-   H51
     6      7      8     23      3     0.65084    1.95253    0.00000   -2.60338    0.00000    0.00000 ;     C3-    C4-    C5-   H52
     7      6      3     13      3     0.65084    1.95253    0.00000   -2.60338    0.00000    0.00000 ;     C4-    C3-    C2-    H2
     7      8      9     10      3     2.84512   -4.10032   16.73600    2.51040  -16.73600    0.00000 ;     C4-    C5-    N6-    C7
     7      8      9     14      3     0.00000    0.00000    0.00000    0.00000    0.00000    0.00000 ;     C4-    C5-    N6-    H6
     8      7      6     18      3     0.66944    2.00832    0.00000   -2.67776    0.00000    0.00000 ;     C5-    C4-    C3-   H31
     8      7      6     19      3     0.66944    2.00832    0.00000   -2.67776    0.00000    0.00000 ;     C5-    C4-    C3-   H32
     8      9     10     11      3    20.92000    0.00000  -20.92000    0.00000    0.00000    0.00000 ;     C5-    N6-    C7-    O7
     8      9     10     12      3    20.92000    0.00000  -20.92000    0.00000    0.00000    0.00000 ;     C5-    N6-    C7-    N8
     9      8      7     20      3     0.65084    1.95253    0.00000   -2.60338    0.00000    0.00000 ;     N6-    C5-    C4-   H41
     9      8      7     21      3     0.65084    1.95253    0.00000   -2.60338    0.00000    0.00000 ;     N6-    C5-    C4-   H42
     9     10     12     24      3    20.92000    0.00000  -20.92000    0.00000    0.00000    0.00000 ;     N6-    C7-    N8-   H81
     9     10     12     25      3    20.92000    0.00000  -20.92000    0.00000    0.00000    0.00000 ;     N6-    C7-    N8-   H82
    10      9      8     22      3     0.00000    0.00000    0.00000    0.00000    0.00000    0.00000 ;     C7-    N6-    C5-   H51
    10      9      8     23      3     0.00000    0.00000    0.00000    0.00000    0.00000    0.00000 ;     C7-    N6-    C5-   H52
    11     10      9     14      3    29.28800   -8.36800  -20.92000    0.00000    0.00000    0.00000 ;     O7-    C7-    N6-    H6
    11     10     12     24      3    29.28800   -8.36800  -20.92000    0.00000    0.00000    0.00000 ;     O7-    C7-    N8-   H81
    11     10     12     25      3    29.28800   -8.36800  -20.92000    0.00000    0.00000    0.00000 ;     O7-    C7-    N8-   H82
    12     10      9     14      3    20.92000    0.00000  -20.92000    0.00000    0.00000    0.00000 ;     N8-    C7-    N6-    H6
    13      3      4     15      3     0.65084    1.95253    0.00000   -2.60338    0.00000    0.00000 ;     H2-    C2-    N2-   H21
    13      3      4     16      3     0.65084    1.95253    0.00000   -2.60338    0.00000    0.00000 ;     H2-    C2-    N2-   H22
    13      3      4     17      3     0.65084    1.95253    0.00000   -2.60338    0.00000    0.00000 ;     H2-    C2-    N2-   H23
    13      3      6     18      3     0.65084    1.95253    0.00000   -2.60338    0.00000    0.00000 ;     H2-    C2-    C3-   H31
    13      3      6     19      3     0.65084    1.95253    0.00000   -2.60338    0.00000    0.00000 ;     H2-    C2-    C3-   H32
    14      9      8     22      3     0.00000    0.00000    0.00000    0.00000    0.00000    0.00000 ;     H6-    N6-    C5-   H51
```

```
   14      9      8     23      3   0.00000   0.00000   0.00000   0.00000   0.00000   0.00000 ;    H6-   N6-   C5-  H52
   18      6      7     20      3   0.62760   1.88280   0.00000  -2.51040   0.00000   0.00000 ;   H31-   C3-   C4-  H41
   18      6      7     21      3   0.62760   1.88280   0.00000  -2.51040   0.00000   0.00000 ;   H31-   C3-   C4-  H42
   19      6      7     20      3   0.62760   1.88280   0.00000  -2.51040   0.00000   0.00000 ;   H32-   C3-   C4-  H41
   19      6      7     21      3   0.62760   1.88280   0.00000  -2.51040   0.00000   0.00000 ;   H32-   C3-   C4-  H42
   20      7      8     22      3   0.65084   1.95253   0.00000  -2.60338   0.00000   0.00000 ;   H41-   C4-   C5-  H51
   20      7      8     23      3   0.65084   1.95253   0.00000  -2.60338   0.00000   0.00000 ;   H41-   C4-   C5-  H52
   21      7      8     22      3   0.65084   1.95253   0.00000  -2.60338   0.00000   0.00000 ;   H42-   C4-   C5-  H51
   21      7      8     23      3   0.65084   1.95253   0.00000  -2.60338   0.00000   0.00000 ;   H42-   C4-   C5-  H52

[ dihedrals ] ; impropers
; treated as propers in GROMACS to use correct AMBER analytical function
;    i      j      k      l   func   phase     kd      pn
     5      1      2      3      1  180.00   4.60240   2 ;     O2-    C1-    O1-    C2
     9     12     10     11      1  180.00  43.93200   2 ;     N6-    N8-    C7-    O7
    10      8      9     14      1  180.00   4.60240   2 ;     C7-    C5-    N6-    H6
    10     24     12     25      1  180.00   4.60240   2 ;     C7-   H81-    N8-   H82
```

Arginine Parameterization

```
[ atomtypes ]
;name   bond_type     mass     charge   ptype   sigma         epsilon       Amb
 n4       n4          0.00000  0.00000   A     3.25000e-01   7.11280e-01 ; 1.82  0.1700
 c3       c3          0.00000  0.00000   A     3.39967e-01   4.57730e-01 ; 1.91  0.1094
 c2       c2          0.00000  0.00000   A     3.39967e-01   3.59824e-01 ; 1.91  0.0860
 o        o           0.00000  0.00000   A     2.95992e-01   8.78640e-01 ; 1.66  0.2100
 n3       n3          0.00000  0.00000   A     3.25000e-01   7.11280e-01 ; 1.82  0.1700
 cz       cz          0.00000  0.00000   A     3.39967e-01   3.59824e-01 ; 1.91  0.0860
 hn       hn          0.00000  0.00000   A     1.06908e-01   6.56888e-02 ; 0.60  0.0157
 hx       hx          0.00000  0.00000   A     1.95998e-01   6.56888e-02 ; 1.10  0.0157
 hc       hc          0.00000  0.00000   A     2.64953e-01   6.56888e-02 ; 1.49  0.0157
 h1       h1          0.00000  0.00000   A     2.47135e-01   6.56888e-02 ; 1.39  0.0157

[ moleculetype ]
;name            nrexcl
 ARG              3

[ atoms ]
;   nr  type  resi  res  atom  cgnr     charge      mass       ; qtot   bond_type
     1   n4     1   ARG     N    1    -1.031055     14.01000 ; qtot -1.031
     2   c3     1   ARG    CA    2     0.413143     12.01000 ; qtot -0.618
     3   c2     1   ARG     C    3     0.726624     12.01000 ; qtot 0.109
     4    o     1   ARG     O    4    -0.589459     16.00000 ; qtot -0.481
     5   c3     1   ARG    CB    5    -0.379750     12.01000 ; qtot -0.860
     6   c3     1   ARG    CG    6    -0.195282     12.01000 ; qtot -1.056
     7   c3     1   ARG    CD    7     0.403002     12.01000 ; qtot -0.653
     8   n3     1   ARG    NE    8    -0.914270     14.01000 ; qtot -1.567
     9   cz     1   ARG    CZ    9     1.112648     12.01000 ; qtot -0.454
    10   n3     1   ARG   NH1   10    -1.040737     14.01000 ; qtot -1.495
    11   n3     1   ARG   NH2   11    -1.040737     14.01000 ; qtot -2.536
    12    o     1   ARG   OXT   12    -0.577815     16.00000 ; qtot -3.114
    13   hn     1   ARG    H1   13     0.444131      1.00800 ; qtot -2.670
    14   hn     1   ARG    H2   14     0.400624      1.00800 ; qtot -2.269
    15   hn     1   ARG    H3   15     0.400624      1.00800 ; qtot -1.868
    16   hx     1   ARG    HA   16    -0.023402      1.00800 ; qtot -1.892
    17   hc     1   ARG   HB2   17     0.135922      1.00800 ; qtot -1.756
    18   hc     1   ARG   HB3   18     0.135922      1.00800 ; qtot -1.620
    19   hc     1   ARG   HG2   19     0.102761      1.00800 ; qtot -1.517
    20   hc     1   ARG   HG3   20     0.102761      1.00800 ; qtot -1.414
    21   h1     1   ARG   HD2   21     0.026011      1.00800 ; qtot -1.388
    22   h1     1   ARG   HD3   22     0.026011      1.00800 ; qtot -1.362
    23   hn     1   ARG    HE   23     0.450098      1.00800 ; qtot -0.912
    24   hn     1   ARG  HH11   24     0.478055      1.00800 ; qtot -0.434
    25   hn     1   ARG  HH12   25     0.478055      1.00800 ; qtot 0.044
    26   hn     1   ARG  HH21   26     0.478055      1.00800 ; qtot 0.522
    27   hn     1   ARG  HH22   27     0.478055      1.00800 ; qtot 1.000

[ bonds ]
;   ai     aj funct   r             k
     1      2   1    1.5110e-01    2.3707e+05 ;      N - CA
     1     13   1    1.0304e-01    3.1229e+05 ;      N - H1
     1     14   1    1.0304e-01    3.1229e+05 ;      N - H2
     1     15   1    1.0304e-01    3.1229e+05 ;      N - H3
     2      3   1    1.5095e-01    2.7347e+05 ;     CA - C
     2      5   1    1.5375e-01    2.5179e+05 ;     CA - CB
     2     16   1    1.0910e-01    2.8342e+05 ;     CA - HA
     3      4   1    1.2247e-01    5.2124e+05 ;      C - O
     3     12   1    1.2247e-01    5.2124e+05 ;      C - OXT
     5      6   1    1.5375e-01    2.5179e+05 ;     CB - CG
     5     17   1    1.0969e-01    2.7665e+05 ;     CB - HB2
     5     18   1    1.0969e-01    2.7665e+05 ;     CB - HB3
     6      7   1    1.5375e-01    2.5179e+05 ;     CG - CD
     6     19   1    1.0969e-01    2.7665e+05 ;     CG - HG2
     6     20   1    1.0969e-01    2.7665e+05 ;     CG - HG3
     7      8   1    1.4647e-01    2.7271e+05 ;     CD - NE
     7     21   1    1.0969e-01    2.7665e+05 ;     CD - HD2
     7     22   1    1.0969e-01    2.7665e+05 ;     CD - HD3
     8      9   1    1.3400e-01    4.0694e+05 ;     NE - CZ
     8     23   1    1.0190e-01    3.2836e+05 ;     NE - HE
```

```
     9     10   1    1.3400e-01    4.0694e+05 ;      CZ - NH1
     9     11   1    1.3400e-01    4.0694e+05 ;      CZ - NH2
    10     24   1    1.0190e-01    3.2836e+05 ;    NH1 - HH11
    10     25   1    1.0190e-01    3.2836e+05 ;    NH1 - HH12
    11     26   1    1.0190e-01    3.2836e+05 ;    NH2 - HH21
    11     27   1    1.0190e-01    3.2836e+05 ;    NH2 - HH22

[ pairs ]
;   ai     aj    funct
     1      4      1 ;      N - O
     1      6      1 ;      N - CG
     1     12      1 ;      N - OXT
     1     17      1 ;      N - HB2
     1     18      1 ;      N - HB3
     2      7      1 ;     CA - CD
     2     19      1 ;     CA - HG2
     2     20      1 ;     CA - HG3
     3      6      1 ;      C - CG
     3     17      1 ;      C - HB2
     3     18      1 ;      C - HB3
     4      5      1 ;      O - CB
     4     16      1 ;      O - HA
     5      8      1 ;     CB - NE
     5     12      1 ;     CB - OXT
     5     21      1 ;     CB - HD2
     5     22      1 ;     CB - HD3
     6      9      1 ;     CG - CZ
     6     16      1 ;     CG - HA
     6     23      1 ;     CG - HE
     7     10      1 ;     CD - NH1
     7     11      1 ;     CD - NH2
     7     17      1 ;     CD - HB2
     7     18      1 ;     CD - HB3
     8     19      1 ;     NE - HG2
     8     20      1 ;     NE - HG3
     8     24      1 ;     NE - HH11
     8     25      1 ;     NE - HH12
     8     26      1 ;     NE - HH21
     8     27      1 ;     NE - HH22
     9     21      1 ;     CZ - HD2
     9     22      1 ;     CZ - HD3
    10     23      1 ;    NH1 - HE
    10     26      1 ;    NH1 - HH21
    10     27      1 ;    NH1 - HH22
    11     23      1 ;    NH2 - HE
    11     24      1 ;    NH2 - HH11
    11     25      1 ;    NH2 - HH12
    12     16      1 ;    OXT - HA
    13      3      1 ;     H1 - C
    13      5      1 ;     H1 - CB
    13     16      1 ;     H1 - HA
    14      3      1 ;     H2 - C
    14      5      1 ;     H2 - CB
    14     16      1 ;     H2 - HA
    15      3      1 ;     H3 - C
    15      5      1 ;     H3 - CB
    15     16      1 ;     H3 - HA
    16     17      1 ;     HA - HB2
    16     18      1 ;     HA - HB3
    17     19      1 ;    HB2 - HG2
    17     20      1 ;    HB2 - HG3
    18     19      1 ;    HB3 - HG2
    18     20      1 ;    HB3 - HG3
    19     21      1 ;    HG2 - HD2
    19     22      1 ;    HG2 - HD3
    20     21      1 ;    HG3 - HD2
    20     22      1 ;    HG3 - HD3
    21     23      1 ;    HD2 - HE
    22     23      1 ;    HD3 - HE

[ angles ]
```

```
;   ai     aj     ak    funct   theta         cth
     1      2      3      1    1.1364e+02    5.4308e+02 ;      N - CA     - C
     1      2      5      1    1.1421e+02    5.3723e+02 ;      N - CA     - CB
     1      2     16      1    1.0801e+02    4.0668e+02 ;      N - CA     - HA
     2      1     13      1    1.1011e+02    3.8409e+02 ;     CA - N      - H1
     2      1     14      1    1.1011e+02    3.8409e+02 ;     CA - N      - H2
     2      1     15      1    1.1011e+02    3.8409e+02 ;     CA - N      - H3
     2      3      4      1    1.2282e+02    5.6819e+02 ;     CA - C      - O
     2      3     12      1    1.2282e+02    5.6819e+02 ;     CA - C      - OXT
     2      5      6      1    1.1151e+02    5.2635e+02 ;     CA - CB     - CG
     2      5     17      1    1.0980e+02    3.8744e+02 ;     CA - CB     - HB2
     2      5     18      1    1.0980e+02    3.8744e+02 ;     CA - CB     - HB3
     3      2      5      1    1.1156e+02    5.3053e+02 ;      C - CA     - CB
     3      2     16      1    1.1134e+02    3.9162e+02 ;      C - CA     - HA
     4      3     12      1    1.2169e+02    6.7111e+02 ;      O - C      - OXT
     5      2     16      1    1.1056e+02    3.8660e+02 ;     CB - CA     - HA
     5      6      7      1    1.1151e+02    5.2635e+02 ;     CB - CG     - CD
     5      6     19      1    1.0980e+02    3.8744e+02 ;     CB - CG     - HG2
     5      6     20      1    1.0980e+02    3.8744e+02 ;     CB - CG     - HG3
     6      5     17      1    1.0980e+02    3.8744e+02 ;     CG - CB     - HB2
     6      5     18      1    1.0980e+02    3.8744e+02 ;     CG - CB     - HB3
     6      7      8      1    1.1104e+02    5.5229e+02 ;     CG - CD     - NE
     6      7     21      1    1.0956e+02    3.8828e+02 ;     CG - CD     - HD2
     6      7     22      1    1.0956e+02    3.8828e+02 ;     CG - CD     - HD3
     7      6     19      1    1.0980e+02    3.8744e+02 ;     CD - CG     - HG2
     7      6     20      1    1.0980e+02    3.8744e+02 ;     CD - CG     - HG3
     7      8      9      1    1.1852e+02    5.4096e+02 ;     CD - NE     - CZ
     7      8     23      1    1.0929e+02    3.9664e+02 ;     CD - NE     - HE
     8      7     21      1    1.0988e+02    4.1422e+02 ;     NE - CD     - HD2
     8      7     22      1    1.0988e+02    4.1422e+02 ;     NE - CD     - HD3
     8      9     10      1    1.2014e+02    6.1086e+02 ;     NE - CZ     - NH1
     8      9     11      1    1.2014e+02    6.1086e+02 ;     NE - CZ     - NH2
     9      8     23      1    1.1938e+02    4.1087e+02 ;     CZ - NE     - HE
     9     10     24      1    1.1938e+02    4.1087e+02 ;     CZ - NH1    - HH11
     9     10     25      1    1.1938e+02    4.1087e+02 ;     CZ - NH1    - HH12
     9     11     26      1    1.1938e+02    4.1087e+02 ;     CZ - NH2    - HH21
     9     11     27      1    1.1938e+02    4.1087e+02 ;     CZ - NH2    - HH22
    10      9     11      1    1.2014e+02    6.1086e+02 ;    NH1 - CZ     - NH2
    13      1     14      1    1.0830e+02    3.3974e+02 ;     H1 - N      - H2
    13      1     15      1    1.0830e+02    3.3974e+02 ;     H1 - N      - H3
    14      1     15      1    1.0830e+02    3.3974e+02 ;     H2 - N      - H3
    17      5     18      1    1.0758e+02    3.2970e+02 ;    HB2 - CB     - HB3
    19      6     20      1    1.0758e+02    3.2970e+02 ;    HG2 - CG     - HG3
    21      7     22      1    1.0846e+02    3.2803e+02 ;    HD2 - CD     - HD3
    24     10     25      1    1.0640e+02    3.4644e+02 ;   HH11 - NH1    - HH12
    26     11     27      1    1.0640e+02    3.4644e+02 ;   HH21 - NH2    - HH22

[ dihedrals ] ; propers
; treated as RBs in GROMACS to use combine multiple AMBER torsions per quartet
;    i      j      k      l   func     C0          C1          C2          C3          C4          C5
     1      2      3      4      3   0.00000     0.00000     0.00000     0.00000     0.00000     0.00000 ;     N-    CA-     C-     O
     1      2      3     12      3   0.00000     0.00000     0.00000     0.00000     0.00000     0.00000 ;     N-    CA-     C-   OXT
     1      2      5      6      3   0.65084     1.95253     0.00000    -2.60338     0.00000     0.00000 ;     N-    CA-    CB-    CG
     1      2      5     17      3   0.65084     1.95253     0.00000    -2.60338     0.00000     0.00000 ;     N-    CA-    CB-   HB2
     1      2      5     18      3   0.65084     1.95253     0.00000    -2.60338     0.00000     0.00000 ;     N-    CA-    CB-   HB3
     2      5      6      7      3   3.68192     3.09616    -2.09200    -3.01248     0.00000     0.00000 ;    CA-    CB-    CG-    CD
     2      5      6     19      3   0.66944     2.00832     0.00000    -2.67776     0.00000     0.00000 ;    CA-    CB-    CG-   HG2
     2      5      6     20      3   0.66944     2.00832     0.00000    -2.67776     0.00000     0.00000 ;    CA-    CB-    CG-   HG3
     3      2      5      6      3   0.65084     1.95253     0.00000    -2.60338     0.00000     0.00000 ;     C-    CA-    CB-    CG
     3      2      5     17      3   0.65084     1.95253     0.00000    -2.60338     0.00000     0.00000 ;     C-    CA-    CB-   HB2
     3      2      5     18      3   0.65084     1.95253     0.00000    -2.60338     0.00000     0.00000 ;     C-    CA-    CB-   HB3
     4      3      2      5      3   0.00000     0.00000     0.00000     0.00000     0.00000     0.00000 ;     O-     C-    CA-    CB
     4      3      2     16      3   0.00000     0.00000     0.00000     0.00000     0.00000     0.00000 ;     O-     C-    CA-    HA
     5      2      3     12      3   0.00000     0.00000     0.00000     0.00000     0.00000     0.00000 ;    CB-    CA-     C-   OXT
     5      6      7      8      3   0.65084     1.95253     0.00000    -2.60338     0.00000     0.00000 ;    CB-    CG-    CD-    NE
     5      6      7     21      3   0.65084     1.95253     0.00000    -2.60338     0.00000     0.00000 ;    CB-    CG-    CD-   HD2
     5      6      7     22      3   0.65084     1.95253     0.00000    -2.60338     0.00000     0.00000 ;    CB-    CG-    CD-   HD3
     6      5      2     16      3   0.65084     1.95253     0.00000    -2.60338     0.00000     0.00000 ;    CG-    CB-    CA-    HA
     6      7      8      9      3   1.25520     3.76560     0.00000    -5.02080     0.00000     0.00000 ;    CG-    CD-    NE-    CZ
     6      7      8     23      3   1.25520     3.76560     0.00000    -5.02080     0.00000     0.00000 ;    CG-    CD-    NE-    HE
     7      6      5     17      3   0.66944     2.00832     0.00000    -2.67776     0.00000     0.00000 ;    CD-    CG-    CB-   HB2
     7      6      5     18      3   0.66944     2.00832     0.00000    -2.67776     0.00000     0.00000 ;    CD-    CG-    CB-   HB3
     7      8      9     10      3   2.51040     0.00000    -2.51040     0.00000     0.00000     0.00000 ;    CD-    NE-    CZ-   NH1
     7      8      9     11      3   2.51040     0.00000    -2.51040     0.00000     0.00000     0.00000 ;    CD-    NE-    CZ-   NH2
     8      7      6     19      3   0.65084     1.95253     0.00000    -2.60338     0.00000     0.00000 ;    NE-    CD-    CG-   HG2
     8      7      6     20      3   0.65084     1.95253     0.00000    -2.60338     0.00000     0.00000 ;    NE-    CD-    CG-   HG3
     8      9     10     24      3   2.51040     0.00000    -2.51040     0.00000     0.00000     0.00000 ;    NE-    CZ-   NH1-  HH11
     8      9     10     25      3   2.51040     0.00000    -2.51040     0.00000     0.00000     0.00000 ;    NE-    CZ-   NH1-  HH12
     8      9     11     26      3   2.51040     0.00000    -2.51040     0.00000     0.00000     0.00000 ;    NE-    CZ-   NH2-  HH21
     8      9     11     27      3   2.51040     0.00000    -2.51040     0.00000     0.00000     0.00000 ;    NE-    CZ-   NH2-  HH22
     9      8      7     21      3   1.25520     3.76560     0.00000    -5.02080     0.00000     0.00000 ;    CZ-    NE-    CD-   HD2
```

```
     9      8      7     22      3    1.25520    3.76560    0.00000   -5.02080    0.00000    0.00000 ;     CZ-    NE-    CD-   HD3
    10      9      8     23      3    2.51040    0.00000   -2.51040    0.00000    0.00000    0.00000 ;    NH1-    CZ-    NE-    HE
    10      9     11     26      3    2.51040    0.00000   -2.51040    0.00000    0.00000    0.00000 ;    NH1-    CZ-   NH2-  HH21
    10      9     11     27      3    2.51040    0.00000   -2.51040    0.00000    0.00000    0.00000 ;    NH1-    CZ-   NH2-  HH22
    11      9      8     23      3    2.51040    0.00000   -2.51040    0.00000    0.00000    0.00000 ;    NH2-    CZ-    NE-    HE
    11      9     10     24      3    2.51040    0.00000   -2.51040    0.00000    0.00000    0.00000 ;    NH2-    CZ-   NH1-  HH11
    11      9     10     25      3    2.51040    0.00000   -2.51040    0.00000    0.00000    0.00000 ;    NH2-    CZ-   NH1-  HH12
    12      3      2     16      3    0.00000    0.00000    0.00000    0.00000    0.00000    0.00000 ;    OXT-     C-    CA-    HA
    13      1      2      3      3    0.65084    1.95253    0.00000   -2.60338    0.00000    0.00000 ;     H1-     N-    CA-     C
    13      1      2      5      3    0.65084    1.95253    0.00000   -2.60338    0.00000    0.00000 ;     H1-     N-    CA-    CB
    13      1      2     16      3    0.65084    1.95253    0.00000   -2.60338    0.00000    0.00000 ;     H1-     N-    CA-    HA
    14      1      2      3      3    0.65084    1.95253    0.00000   -2.60338    0.00000    0.00000 ;     H2-     N-    CA-     C
    14      1      2      5      3    0.65084    1.95253    0.00000   -2.60338    0.00000    0.00000 ;     H2-     N-    CA-    CB
    14      1      2     16      3    0.65084    1.95253    0.00000   -2.60338    0.00000    0.00000 ;     H2-     N-    CA-    HA
    15      1      2      3      3    0.65084    1.95253    0.00000   -2.60338    0.00000    0.00000 ;     H3-     N-    CA-     C
    15      1      2      5      3    0.65084    1.95253    0.00000   -2.60338    0.00000    0.00000 ;     H3-     N-    CA-    CB
    15      1      2     16      3    0.65084    1.95253    0.00000   -2.60338    0.00000    0.00000 ;     H3-     N-    CA-    HA
    16      2      5     17      3    0.65084    1.95253    0.00000   -2.60338    0.00000    0.00000 ;     HA-    CA-    CB-   HB2
    16      2      5     18      3    0.65084    1.95253    0.00000   -2.60338    0.00000    0.00000 ;     HA-    CA-    CB-   HB3
    17      5      6     19      3    0.62760    1.88280    0.00000   -2.51040    0.00000    0.00000 ;    HB2-    CB-    CG-   HG2
    17      5      6     20      3    0.62760    1.88280    0.00000   -2.51040    0.00000    0.00000 ;    HB2-    CB-    CG-   HG3
    18      5      6     19      3    0.62760    1.88280    0.00000   -2.51040    0.00000    0.00000 ;    HB3-    CB-    CG-   HG2
    18      5      6     20      3    0.62760    1.88280    0.00000   -2.51040    0.00000    0.00000 ;    HB3-    CB-    CG-   HG3
    19      6      7     21      3    0.65084    1.95253    0.00000   -2.60338    0.00000    0.00000 ;    HG2-    CG-    CD-   HD2
    19      6      7     22      3    0.65084    1.95253    0.00000   -2.60338    0.00000    0.00000 ;    HG2-    CG-    CD-   HD3
    20      6      7     21      3    0.65084    1.95253    0.00000   -2.60338    0.00000    0.00000 ;    HG3-    CG-    CD-   HD2
    20      6      7     22      3    0.65084    1.95253    0.00000   -2.60338    0.00000    0.00000 ;    HG3-    CG-    CD-   HD3
    21      7      8     23      3    1.25520    3.76560    0.00000   -5.02080    0.00000    0.00000 ;    HD2-    CD-    NE-    HE
    22      7      8     23      3    1.25520    3.76560    0.00000   -5.02080    0.00000    0.00000 ;    HD3-    CD-    NE-    HE

[ dihedrals ] ; impropers
; treated as propers in GROMACS to use correct AMBER analytical function
;    i      j      k      l   func   phase     kd      pn
     2      4      3     12      1   180.00   4.60240   2 ;      CA-     O-      C-   OXT
     8     10      9     11      1   180.00   4.60240   2 ;      NE-   NH1-     CZ-   NH2
```
